# Effectiveness and active ingredients of digital behaviour change interventions for MASLD: A systematic review and meta-analysis

**DOI:** 10.1016/j.jhepr.2025.101507

**Published:** 2025-07-02

**Authors:** Hollie Smith, Rebecca Livingston, Kirsten Ashley, Matthew Cooper, Stuart McPherson, Alison Innerd, Kate Hallsworth, Leah Avery

**Affiliations:** 1 School of Health and Life Sciences, Teesside University, Middlesbrough, UK; 2 School of Social Sciences, Humanities and Law, Teesside University, Middlesborough, UK; 3 NIHR Newcastle Patient Safety Research Collaborative, Newcastle University, Newcastle upon Tyne, UK; 4 NIHR Newcastle Biomedical Research Centre, Newcastle upon Tyne Hospitals NHS Foundation Trust, Newcastle upon Tyne, UK; 5 Liver Unit, Newcastle upon Tyne Hospitals NHS Foundation Trust, Newcastle upon Tyne, UK; 6 Translational and Clinical Research Institute, Faculty of Medical Sciences, Newcastle University, Newcastle upon Tyne, UK

**Keywords:** Steatotic liver disease, NAFLD, MASH, Diet, Physical activity

## Abstract

**Background & Aims:**

Metabolic dysfunction-associated steatotic liver disease (MASLD) is the most prevalent liver condition worldwide. Successful management relies on targeting changes in lifestyle behaviours. Digital behaviour change interventions present a scalable approach to lifestyle change. The aim of this systematic review was to determine the effectiveness and active ingredients of digital behavior change interventions for improving weight and liver-related outcome measures in patients with MASLD.

**Methods:**

Five databases were searched up to 31 January 2025 for studies reporting on digital lifestyle behaviour change interventions for patients with MASLD. Data were meta-analysed or narratively synthesised depending on study design. Intervention content and features positively associated with changes in outcomes of interest were identified using promise analysis.

**Results:**

Eleven studies involving 1,288 participants fulfilled the review criteria. Digital behavior change interventions were not effective for reducing weight (weighted mean difference [WMD] -2.07 kg [-6.08 to 1.94 kg]). Likewise, they did not lead to statistically significant improvements in alanine transaminase and aspartate transaminase (WMD -9.14 [-20.33 to 2.05] and WMD -5.81 [-12.96 to 1.35], respectively). Interventions varied in terms of mode of delivery (*e.g.* app and SMS), duration (1–11 months), and frequency of delivery (three times/week to continuous access). Promising intervention features/content included app-based delivery, ≥6-month duration, and self-monitoring of behaviour, feedback on outcomes, and social support.

**Conclusions:**

Digital behaviour change interventions did not improve weight and liver-related outcomes measures in patients with MASLD. However, the inclusion of proposed specific intervention ingredients is likely to improve effectiveness.

**Impact and implications:**

This review is the first of its kind to report on the effectiveness and active ingredients of digital behaviour change interventions for the management of MASLD. Although the interventions reviewed were not effective overall, specific features and content of those interventions were associated with effectiveness. These insights can be used to inform the development of new interventions or to optimise existing interventions that could improve effectiveness. Findings also suggest that digital behaviour change interventions are beneficial for a proportion of individuals, and future research should focus on identifying who those individuals are. Significant heterogeneity between interventions was evident in terms of mode of delivery, behavioural change content, duration, and frequency of delivery. To truly determine the effectiveness of digital behaviour change interventions for patients with MASLD, they should be systematically developed using behaviour change theory and in accordance with a recognised intervention development framework.

## Highlights

• Eleven digital behaviour change interventions to support patients with MASLD were identified by the review.
• Successful management of MASLD relies on targeting change in lifestyle behaviours, but behaviours were rarely measured.
• Digital interventions were not effective for improving weight, ALT, or AST.
• App-based delivery, ≥6-month duration, and self-regulation content were associated with intervention effectiveness.
• Inclusion of specific intervention ingredients increases the likelihood of effectiveness.

## Graphical abstract

**Figure:**
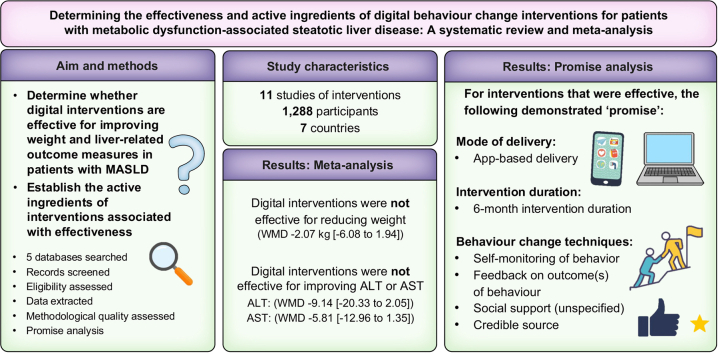

## Introduction

Metabolic dysfunction-associated steatotic liver disease (MASLD) is defined as the presence of hepatic steatosis on imaging or histology in conjunction with at least one cardiometabolic risk factor and no other discernible cause.^1^ MASLD is the most common liver condition, affecting upwards of 30% of adults globally,^2^ and is related to lifestyle factors such as regular consumption of energy-dense, ultra-processed foods and low levels of physical activity that contribute towards overweight and obesity.^3^

Despite the prevalence of MASLD, management of the condition is variable.^4^ Clinical practice guidelines recommend lifestyle modification (*i.e*. changes to diet and physical activity behaviours) to facilitate weight loss for all patients with MASLD.^3^ A reduction in body weight of ≥5% is associated with a reduction in liver fat, a reduction of 7–10% is associated with an improvement in liver inflammation, and a reduction of ≥10% is associated with an improvement in fibrosis.^5^

However, despite the dose–response relationship between the amount of weight lost and the extent of improvement in liver disease biomarkers,^6^ changing lifestyle behaviours remains a challenge, and many patients do not achieve guideline-recommended weight loss goals.^7^^,^^8^ Barriers to changing lifestyle behaviours can be attributed to many factors, including patients not receiving appropriate support, stigma, comorbidities, constraints on time preventing attendance at appointments (*e.g.* long working hours and caring responsibilities), and access to healthcare and other facilities (*e.g.* distance to clinic and appointment availability) that are not conducive to facilitating, enacting, and sustaining lifestyle behaviour change.[9], [10], [11] In the context of MASLD, healthcare professionals (HCPs) have raised concerns about supporting patients to make changes to their lifestyle behaviours because of a lack of specific training, appropriate interventions, and referral pathways.[12], [13], [14] Furthermore, the prevalence of MASLD globally highlights the necessity to scale up clinical services/interventions to meet growing demands in a complex patient population.

Digital technology allows health services and interventions to be delivered or enriched via the internet and mobile applications at scale.^15^ Digital healthcare delivery is an innovative and cost-effective method to meet the demand for long-term support for those living with chronic conditions.^16^ Several meta-analyses have highlighted the positive effect of digital interventions on measures of health,^17^^,^^18^ and digital lifestyle interventions are currently being used to support patients at risk of other long-term conditions. An example of such an intervention is the Diabetes Prevention Programme (DPP). The DPP was created by the UK National Health Service (NHS) and Diabetes UK and provides a 9-month, evidence-based digital lifestyle behaviour change programme^19^ for individuals at risk of type 2 diabetes. It includes personalised support using digital tools, such as apps and wearable technologies, and provides health coaches and online peer support groups that aim to support patients to change and maintain changes in lifestyle behaviours. The DPP has proven to be effective, evidenced by clinically significant reductions in weight and HbA_1c_, and acceptable to patients.^20^

A recent systematic review reported on the effectiveness of digital behaviour change interventions for facilitating weight loss in patients with MASLD.^21^ Although the findings concluded that the interventions were effective overall, the analysis included only the intervention arms of eight studies with small sample sizes in the meta-analysis. They were single-group studies (n = 4), randomised controlled trials (RCTs) (n = 3), and non-RCTs (n = 1). Furthermore, the specific features and active ingredients of the digital behaviour change interventions (*i.e.* those that contribute to intervention effectiveness) were not identified or reported. As such, the aim of this systematic review was to determine the effectiveness of digital behaviour change interventions for improving weight and liver-related outcomes in patients diagnosed with MASLD and to identify the active ingredients of these interventions, namely, the specific features and content that lead to changes in the outcomes of interest. The specific research questions were as follows:

1. Are digital behaviour change interventions effective for improving weight in patients with MASLD?
2. Are digital behaviour change interventions effective for improving liver-related outcome measures in patients with MASLD?
3. What are the active ingredients of digital behaviour change interventions for patients with MASLD that lead to improvements in weight, metabolic outcomes, and/or liver-related outcome measures?

## Materials and methods

### Search strategy and study selection

This systematic review was conducted with reference to a registered protocol (CRD42023406827),^22^ and adhered to the Preferred Reporting Items for Systematic Reviews and Meta-Analysis (PRISMA) guidelines^23^ (Supplementary file 1).

Five electronic databases (MEDLINE, CINAHL, Web of Science, PsycINFO, and SCOPUS) were searched from inception to 31 January 2025 using a combination of MeSH headings and keywords (Supplementary file 2). No limits or restrictions were applied. Before conducting the search, scoping searches were performed to refine the search strategy. Manual searches of end reference lists and citation searches of included studies were conducted to identify potentially relevant studies not captured by the electronic search.

Search results underwent a process of electronic deduplication that was checked for accuracy by one reviewer (HS). The same reviewer screened the titles and abstracts of all references retrieved by the search, and a second reviewer (RL) independently screened a 20% random sample (Stage 1 screening). Articles retained following screening of titles and abstracts were retrieved in full text and independently screened by HS and one of four reviewers (RL, LA, SMc, or KH) against eligibility criteria using a study selection form (Stage 2 screening) (Supplementary file 3). Disagreements between reviewers were resolved by discussion.

### Eligibility criteria

Studies reporting on a digital (*e.g.* web, app, SMS, instant messaging, e-mail, and video conferencing) behaviour change interventions designed to facilitate improvements in weight and liver-related outcome measures in patients aged ≥18 years with MASLD or metabolic dysfunction-associated steatohepatitis (MASH) were included in the review. Studies reporting on participants with non-alcoholic fatty liver disease were also eligible for inclusion. It was a requirement for studies to report the impact of the digital intervention on weight, metabolic outcomes (*e.g.* HbA_1c_, high-density lipoprotein [HDL], low-density lipoprotein [LDL], triglycerides [TGs], and total cholesterol), and liver-related measures (*e.g.* hepatic fibrosis using liver stiffness measurement [LSM], hepatic steatosis using controlled attenuation parameter [CAP], liver fat % using magnetic resonance imaging proton density fat fraction [MRI-PDFF], and liver enzymes, including alanine transaminase [ALT], aspartate aminotransferase [AST], alkaline phosphatase [ALP], gamma-glutamyl transferase [GGT], and albumin). In instances where studies reported on the impact of the digital intervention on lifestyle behaviours (*e.g.* physical activity, diet, and alcohol), data were extracted and reported. No limits were applied to year or country of publication.

### Data extraction

A standardised data extraction form (Supplementary file 4) was developed to capture the following information: study characteristics (country of origin, aims, design, eligibility criteria, sampling method, sample size, follow-up period, and loss to follow-up), patient characteristics (age, sex/gender, ethnicity, BMI, and comorbidities), intervention characteristics (name, mode of digital delivery, intervention frequency, duration, content, and use of theory in intervention design), outcomes assessed, including findings, and theory-linked behaviour change techniques (BCTs), that is, theory-linked strategies for changing one or more mechanism of action impacting determinants of behaviour (*e.g.* attitudes and self-efficacy), and subsequently behaviour itself.^24^ The data extraction form was piloted by two reviewers (HS and KA) using one included study and subsequently refined.

Data were extracted from studies by one reviewer (HS) and checked by a second reviewer (RL, LA, SMc, KH, or AI). One reviewer (HS) independently coded the presence of BCTs within interventions using the Behaviour Change Technique Taxonomy version 1 (BCTTv1).^24^ BCTTv1 is an extensive taxonomy of 93 consensually agreed, distinct BCTs that offers a method for specifying interventions. A second reviewer (KA) checked 50% of the extracted information for accuracy. Both reviewers had previously completed the BCTTv1 online training, and one reviewer was an experienced coder (KA). Only BCTs included in the interventions that were over and above the standard care/control/comparator group were coded; that is, BCTs present in both the intervention and standard care/control/comparator were not coded.

### Methodological quality assessment

The methodological quality of all included studies was independently assessed by HS and one other reviewer (KA, MC, or LA) using the revised experimental Risk of Bias tool for Randomised Trials (ROB-2),^25^ the Risk of Bias In Non-randomised Studies of Interventions (ROBINS-I) tool,^26^ or the National Heart, Lung, and Blood Institute (NHLBI) Quality Assessment Tool for Before–After (Pre–Post) Studies with No Control Group.^27^ A third reviewer was consulted to resolve any discrepancies.

### Data synthesis

The *Cochrane Handbook for Systematic Reviews of Interventions*^28^ suggests that the results from different study designs should be expected to differ systematically, resulting in increased heterogeneity. As such, the handbook recommends that RCTs and non-randomised experimental studies (*e.g.* single-group pre–post studies) should not be combined with RCTs in a meta-analysis. Therefore, data on changes in weight, ALT, and AST from RCTs only were synthesised using a random-effects meta-analysis in Review Manager (RevMan 8.14.0, available at revman.cochrane.org). Outcomes of single-group pre–post studies and non-randomised studies were synthesised narratively using the Synthesis Without Meta-Analysis Guidelines (SWiM; Supplementary files 5 and 6).

### Promise of intervention content and features

To facilitate the design of effective interventions, it is important to understand the specific content and features that are associated with effectiveness, that is, their active ingredients^29^ (*e.g.* mode of delivery, duration, intensity and frequency of delivery, and specific BCTs). To achieve this, we calculated promise ratios.^30^^,^^31^ Promise analysis is an accepted method for identifying the features/content of interventions associated with positive outcomes/effectiveness.

Firstly, interventions were classified as very promising (statistically significant between-group improvements in weight/metabolic outcomes and/or liver-related outcome measures in favour of the intervention group); quite promising (intervention groups with statistically significant within-group improvements in weight/metabolic outcomes and/or liver-related outcome measures, or improvements greater than those seen in standard care/control/comparator); or non-promising (no statistically significant within- or between-group improvements in weight/metabolic outcomes and/or liver-related measures).

To calculate a ‘promise ratio’, very or quite promising interventions that contained a specific active ingredient were summed and subsequently divided by the number of non-promising interventions that contained the same active ingredient. Active ingredients found in at least twice as many very or quite promising interventions compared with non-promising interventions were classified as promising (promise ratio of ≥2).^30^

## Results

**Fig. 1:**
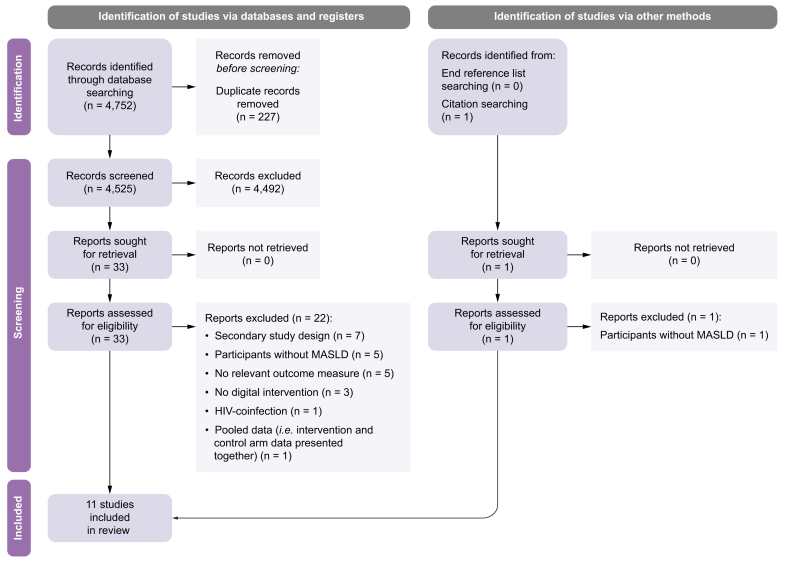
PRISMA flow diagram. MASLD, metabolic dysfunction-associated steatotic liver disease; PRISMA, Preferred Reporting Items for Systematic reviews and Meta-Analyses.

The study selection process is presented in Fig. 1. Eleven articles reporting on 11 digital behaviour change interventions involving 1,288 participants met the eligibility criteria and were retained for review.[32], [33], [34], [35], [36], [37], [38], [39], [40], [41], [42] Six studies were RCTs,^32^^,^[34], [35], [36], [37]^,^^41^ four were single-group pre–post studies,^33^^,^^39^^,^^40^^,^^42^ and one was a non-RCT^38^.

**Fig. 2:**
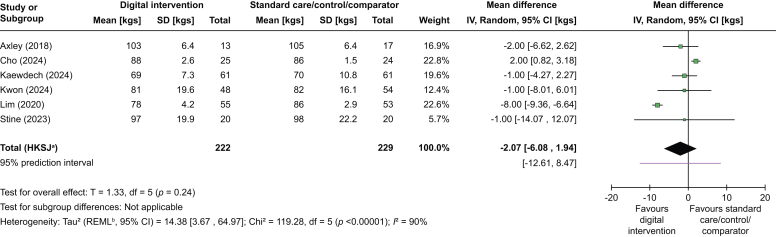
Random-effects meta-analysis for weight (kg).

### Study characteristics

A summary of all included studies is reported in Table 1. Studies were published between 2018 and 2024. They were conducted in the USA,^32^^,^^39^^,^^41^^,^^42^ South Korea,^34^^,^^36^ Italy,^38^ Iceland,^33^ Japan,^40^ Singapore,^37^ and Thailand.^35^ Five interventions were delivered via app,^33^^,^^34^^,^^37^^,^^40^^,^^41^ one of which was delivered alongside the provision of a daily partial meal replacement product.^34^ Two interventions were delivered via SMS/instant messaging,^32^^,^^35^ two interventions were delivered via a combination of modes of delivery (*i.e.* app and SMS^36^ and app and e-mail^42^), one intervention was delivered via a website,^38^ and one intervention was delivered via video software.^39^ The duration of intervention delivery varied from 1 month,^34^ 3 months,^33^ 4 months,^41^ 5 months,^3^ 6 months,^32^^,^[35], [36], [37], [38]^,^^42^ to 11 months^40^.

**Table 1:** Summary of study characteristics.

Study ID, country of origin, study design	Sample details and demographics	Intervention group	Intervention mode of delivery, duration, and frequency of access	Standard care/control/comparator group	BCTs coded in intervention group over and above standard care/control/comparator group	Change in weight and metabolic outcomes from baseline to all follow-up points∗	Change in liver-related outcome measures from baseline to all follow-up points∗
**Axley *et al.* (2018)**^32^ USA Pilot RCT	Total sample: 30 • Intervention: 13 enrolled; 13 analysed • Control: 17 enrolled; 17 analysed Age (years), mean (SD): • Intervention: 54 (2.7) • Control: 52 (2.3) Female, n (%): • Intervention: 11 (85) • Control: 8 (47) BMI (kg/m^2^), mean (SD): • Intervention: 39 (2.4) • Control: 36 (1.9)	Intervention name: No name reported Intervention content: education on nutrition, exercise, stress, and weight management; prompts to set goals with actionable tips provided; information about overcoming barriers with actionable tips Theoretical underpinning: not reported	Mode of delivery: SMS/instant messaging Duration: 6 months Frequency: 3 uni- and bi-directional SMS/week for 6 months	Standard care/control/comparator content: standard of care for liver disease with detailed instructions on healthy diet and daily exercise for weight loss	**1.2** Problem solving **1.3** Goal setting (outcome) **3.1** Social support (unspecified) **7.1** Prompts/cues	6-month follow-up: Mean weight (lbs) • **Intervention: -6** • Control: +2 • **Between groups** Mean HDL (mg/dl) • Intervention: -2 • Control: 0 • Between groups Mean TGs (g/dl) • **Intervention: -17** • Control: -41 • Between groups	6-month follow-up: Mean ALT (IU/L) • **Intervention: -12** • Control: -6 • Between groups Mean AST (IU/L) • **Intervention: -9** • Control: 0 • Between groups
**Björnsdottir *et al.* (2024)**^33^ Iceland Single-group pre–post study	Total sample: 38 • Intervention: 38 enrolled; 38 analysed Age (years), median (IQR): • Intervention: 59.5 (46.3–68.8) Female, n (%): • Intervention: 23 (61) BMI (kg/m^2^), mean (SD): • Intervention: 37.6 (5.8)	Intervention name: SK-241 Intervention content: education videos; self-regulation tools (food journal, step counter, and quality of life rating scale); mindfulness and meditation; health coaching Theoretical underpinning: not reported	Mode of delivery: app Duration: 3 months Frequency: continuous access	Standard care/control/comparator content: N/A	**2.2** Feedback on behaviour **2.3** Self-monitoring of behaviour **3.1** Social support (unspecified)	3-month follow-up: Mean weight (kg) • **Intervention: -3.5** Median HbA_1c_ (mmol/L) • **Intervention: 0.5** Mean HDL (mmol/L) • Intervention: +0.01 Mean LDL (mmol/L) • Intervention: 0 Median TGs (mmol/L) • **Intervention: -0.20** Mean total cholesterol (mmol/L) • Intervention: -0.1	3-month follow-up: Median ALT (IU/L) • Intervention: +1.8 Median AST (IU/L) • Intervention: +1.5 Mean fat %: MRI-PDFF (%) • **Intervention: -2.2** Median fibrosis: LSM (kPa) • Intervention: +0.2 Mean steatosis: CAP (dB/m) **-33.3**
**Cho *et al.* (2024)**^34^ South Korea Pilot RCT	Total sample: 60 • Intervention: 30 enrolled; 25 analysed • Control: 30 enrolled; 24 analysed Age (years), mean (SD): • Intervention: 41.3 (6.7) • Control: 43.5 (6.7) Female (n, %): • Intervention: 0 (0) • Control: 3 (12.5) BMI (kg/m^2^), mean (SD): • Intervention: 30 (4.5) • Control: 30.2 (3.7)	Intervention name: Dr. Coach Intervention content: personalised nutrition prescription, self-monitoring of weight diet, activity levels, emotions, and sleep; health coaching to offer knowledge education, goal setting and planning support, monitoring and feedback, and helping actions (i.e. reminders, alternatives for obstacles); 1/day partial meal replacement Theoretical underpinning: not reported	Mode of delivery: app Duration: 1 month Frequency: continuous access to app; 1 evening meal partial meal replacement/day	Standard care/control/comparator content: Standard care consisting of brief education provided by healthcare providers	**1.1** Goal setting (behaviour) **1.4** Action planning **2.2** Feedback on behaviour **2.3** Self-monitoring of behaviour **2.4** Self-monitoring of outcome(s) of behaviour **2.7** Feedback on outcome(s) of behaviour **3.1** Social support (unspecified) **7.1** Prompts/cues **8.3** Habit formation **8.4** Habit reversal **12.5** Adding objects to the environment	1-month follow-up: Mean weight (kg) • Intervention: -2.93 • Control: -2.74 • Between groups Mean HDL (mmol/L) • Intervention: -1.2 • Control: -1.04 • Between groups Mean LDL (mmol/L) • Intervention: -2.95 • Control: -1.39 • Between groups Mean TGs (mmol/L) • Intervention: -43.84 • Control: -25.96 • Between groups Mean total cholesterol (mmol/L) • Intervention: -12.92 • Control: -5.54 • Between groups	1-month follow-up: Mean ALT (IU/L) • **Intervention: -28.32** • Control: -10.67 • **Between groups** Mean AST (IU/L) • **Intervention: -13.28** • Control: -4.25 • Between groups Mean ALP (IU/L) • Intervention: -0.96 • Control: +1.46 • Between groups Mean GGT (IU/L) • **Intervention: -27.76** • Control: +2.79 • **Between groups** Mean albumin (IU/L) • Intervention: -0.06 • Control: +0.07 • **Between groups**
**Kaewdech *et al.* (2024)**^35^ Thailand RCT	Total sample: 122 • Intervention: 61 enrolled; 61 analysed • Control: 61 enrolled; 61 analysed Age (years), median (IQR): • Intervention: 53.8 (46.6–57.4) • Control: 52.9 (43.2– 59.7) Female, n (%): • Intervention: 38 (62.3) • Control: 41 (67.2) BMI (kg/m^2^), median (IQR): • Intervention: 28 (24.8–30.1) • Control: 27.1 (24.9–29.3)	Intervention name: LINE Intervention content: standard briefing video clip; knowledge broadcast about diet, physical activity, and exercise via video, infographics, and text; reminders to carry out lifestyle changes that were broadcast via messages Theoretical underpinning: Not reported	Mode of delivery: SMS/instant messaging Duration: 6 months Frequency: 3–7 instant messages/week	Standard care/control/comparator content: access to standard briefing video clip (broadcast to all patients at enrolment)	**7.1** Prompts/cues	6-month follow-up: Median weight (kg) • **Intervention: -1** • **Control: -2.2** • Between groups	6-month follow-up: Median ALT (IU/L) • **Intervention: -5** • **Control: -14** • Between groups Median fibrosis: LSM (kPa) • **Intervention: -0.4** • Control: -0.2 • **Between groups** Mean steatosis: CAP (dB/m) • **Intervention: -20.2** • **Control: -25** • Between group
**Kwon *et al.* (2024)**^36^ South Korea RCT	Total sample: 111 • Intervention: 51 enrolled; 48 analysed • Control: 60 enrolled; 54 analysed Age (years), mean (SD): • Intervention: 51 (13.4) • Control: 47.1 (13.9) Female, n (%): • Intervention: 17 (29) • Control: 31 (46) BMI (kg/m^2^), mean (SD): • Intervention: 29.9 (4.1) • Control: 29.6 (4.6)	Intervention name: SMART-Liver Intervention content: self-monitoring of nutritional intake, exercise, sleep, alcohol intake, and smoking; educational slides for MASLD treatment, nutrition, and exercise; health coaching to set goals, identify barriers/facilitators to behaviour change, and provide feedback; Daily SMS to remind participants about daily ‘missions’; real-time chat channel Theoretical underpinning: not reported	Mode of delivery: app and SMS Duration: 6 months Frequency: continuous access to app; 1 SMS/day	Standard care/control/comparator content: phone call after enrolment to obtain weight and inform on the importance of weight loss, diet, and exercise for MASLD management; standard clinical care conducted for 6 months, with contact at 3 and 6 months to obtain weight data	**1.1** Goal setting (behaviour) **1.3** Goal setting (outcomes) **1.4** Action planning **1.5** Review behaviour goal(s) **1.7** Review outcome goal(s) **2.2** Feedback on behaviour **2.3** Self-monitoring of behaviour **3.1** Social support (unspecified) **4.1** Instruction on how to perform the behaviour **6.1** Demonstration of the behaviour **7.1** Prompts/cue **9.1** Credible source **10.4** Social reward	6-month follow-up: Mean weight (kg) • **Intervention: -2.5** • Control: -2.1 • Between groups	6-month follow-up: Mean ALT (IU/L) • **Intervention: -8.1** • Control: -5.6 • Between groups Mean AST (IU/L) • **Intervention: -4.1** • Control: -0.9 • Between groups Mean GGT (IU/L) • **Intervention: -12.2** • Control: -4 • Between groups
**Lim *et al.* (2020)**^37^ Singapore RCT	Total sample: 108 • Intervention: 55 enrolled; 55 analysed • Control: 53 enrolled; 53 analysed Age (years), mean (SD): • Intervention: 46.8 (11.1) • Control: 46.2 (10.1) Female, n (%): • Intervention: 23 (42) • Control: 17 (32) BMI (kg/m^2^), mean (SD): • Intervention: 30.1 (4) • Control: 30.8 (4.8)	Intervention name: Nutritionist Buddy (nBuddy) Intervention content: Self-regulation tools (food diary, step recorder and goal setting, weight logging); feedback from dietitians; peer support chat channel; information (video education, dietary recommendations); daily prompts to log meals Theoretical underpinning: Obesity-Related Behavioural Intervention Trials	Mode of delivery: app Duration: 6 months Frequency: continuous access	Standard care/control/comparator content: standard care advice on dietary and physical activity modification as per guidelines at a single face-to-face session	**2.2** Feedback on behaviour **2.3** Self-monitoring of behaviour **2.7** Feedback on outcome(s) of behaviour **3.1** Social support (unspecified) **7.1** Prompts/cues **9.1** Credible source **10.4** Social reward	3-month follow-up: Mean weight (kg) • Intervention: -3.2 • Control: -0.8 • **Between groups** 6-month follow-up: Mean weight (kg) • Intervention: -3.2 • Control: -0.5 • **Between groups**	3-month follow-up: Mean ALT (IU/L) • Intervention: -37.2 • Control: -20.7 • **Between groups** Mean AST (IU/L) • Intervention: -20.2 • Control: -11 • Between groups 6-month follow-up: Mean ALT (IU/L) • Intervention: -33.5 • Control: -11.5 • **Between groups** Mean AST (IU/L) • Intervention: -17.4IU/L • Control: -7.4IU/L **Between groups**
**Mazzotti *et al.* (2018)**^38^ Italy Two-group non-RCT	Total sample: 716 • Intervention: 278 enrolled; 211 analysed at 6 months; 160 analysed at 12 months; 118 analysed at 24 months • Control: 438 enrolled; 383 analysed at 6 months; 352 analysed at 12 months; 301 analysed at 24 months Age (years), mean (SD): • Intervention: 46.0 (11.5) • Control: 55.1 (12.3) Female (%): • Intervention: 33.1 • Control: 55 BMI (kg/m^2^), mean (SD): • Intervention: 33.7 (6) • Control: 33.2 (5.2)	Intervention name: No name reported Intervention content: educational slides (energy balance, nutrients and weight monitoring; alimentary pyramid and portion size; food shopping, food labels; physical activity, when and how much); tests of information; gamification; ability to send food diaries to clinical centre Theoretical underpinning: not reported	Mode of delivery: Web Duration: 5-weeks Frequency: 1 session/week with content accessible continuously thereafter	Standard care/control/comparator content: 1 120 min group counselling session/week for 5-weeks chaired by physicians and dietitians. Educational slides (energy balance, nutrients and weight monitoring; alimentary pyramid and portion size; food shopping, food labels; physical activity, when and how much); behavioural strategies for weight loss maintenance	**2.1** Monitoring of behaviour by others without feedback	6-month follow-up: Mean weight (%) • Intervention: -3.4 • Control: -3.1 • **Between groups** Mean TGs (mg/dl) • Intervention: -15.6 • Control: -23.5 • Between groups 12-month follow-up: Mean weight (%) • Intervention: -4.9 • Control: -4 • **Between groups** Mean TGs (mg/dl) • Intervention: -24.9 • Control: -27.9 • Between groups 24-month follow-up: Mean weight (%) • Intervention: -5.5 • Control: -4.2 • **Between groups** Mean TGs (mg/dl) • Intervention: -23.5 • Control: -26.6 • Between groups	6-month follow-up: Mean ALT (IU/L) • Intervention: -14.3 • Control: -17.1 • Between groups Mean GGT(IU/L) • Intervention: -7.4 • Control: -12.6 • **Between groups** 12-month follow-up: Mean ALT (IU/L) • Intervention: -18.5 • Control: -18.5 • Between groups Mean GGT(IU/L) • Intervention: -16.4 • Control: -15.2 • Between groups 24-month follow-up: Mean ALT (IU/L) • Intervention: -22 • Control: -19.4 • Between groups Mean GGT(IU/L) • Intervention: -23.5 • Control: -16.1 • **Between groups**
**Motz *et al.* (2021)**^39^ USA Single-group pre–post study	Total sample: 3 • Intervention: 3 enrolled; 3 analysed Age (years), mean (SD): • Intervention: 52 (14) Female, n (%): • Intervention: 3 (100) BMI (kg/m^2^), mean (SD): • Intervention: 31.9 (5.1)	Intervention name: NASHFit Intervention content: supervised aerobic exercise; provision of fitness trackers; feedback from exercise physiologist; dietary counselling and nutritional feedback Theoretical underpinning: not reported	Mode of delivery: video software Duration: 5 months Frequency: five 30-min supervised exercise sessions/week	Standard care/control/comparator content: N/A	**2.2** Feedback on behaviour **2.6** Biofeedback **3.1** Social support (unspecified) **9.1** Credible source **12.5** Adding objects to the environment	5-month follow-up: Mean weight (%) • Intervention: -5.1 Mean HbA_1c_ (%) • Intervention: -0.5	5-month follow-up: Mean ALT (IU/L) • Intervention: -12.5 Mean AST (IU/L) • Intervention: -8.5 Mean fat %: MRI-PDFF (%) • Intervention: -35.1
**Sato *et al.*, (2023)**^40^ Japan Single-group pre–post study	Total sample: 20 • Intervention: 20 enrolled; 19 analysed Age (years), mean (SD): • Intervention: 52.16 (10.77) Female, n (%): • Intervention: 9 (47.4) BMI (kg/m^2^), mean (SD): • Intervention: 32.04 (4.19)	Intervention name: NASH App Intervention content: education; app-based support and instruction to implement lifestyle advice; self-planning and evaluation to encourage maintenance of behavioural change Theoretical underpinning: not reported	Mode of delivery: app Duration: 11 months Frequency: continuous access	Standard care/control/comparator content: N/A	**2.4** Self-monitoring of outcome(s) of behaviour **3.1** Social support (unspecified) **4.1** Instruction on how to perform the behaviour	6-month follow-up: Mean weight (kg) • **Intervention: -6** Mean HbA_1c_ (%) • Intervention: -0.26 Mean LDL (mg/dl) • Intervention: +0.37 Mean TGs (mg/dl) • Intervention: -1.32 11-month follow-up: Mean weight (kg) • **Intervention: -7.3** Mean HbA_1c_ (%) • Intervention: -0.27 Mean LDL (mg/dl) • Intervention: -6 Mean TGs (mg/dl) **Intervention: -19.89**	6-month follow-up: Mean ALT (IU/L) • **Intervention: -40.84** Mean AST (IU/L) • **Intervention: -18.84** Mean ALP (IU/L) • **Intervention: -8.84** Mean GGT (IU/L) • **Intervention: -23.89** 11-month follow-up: Mean ALT (IU/L) • **Intervention: -49.53** Mean AST (IU/L) • **Intervention: -23.05** Mean ALP (IU/L) • **Intervention: -10.63** Mean GGT (IU/L) • **Intervention: -28.63**
**Stine *et al.* (2023)**^41^ USA Pilot RCT	Total sample: 40 • Intervention: 20 enrolled; 20 analysed • Control: 20 enrolled; 20 analysed Age (years), mean (SD): • Intervention: 53.3 (13.3) • Control: 50.4 (12.1) Female, n (%): • Intervention: 12 (60%) • Control: 17 (85%) BMI (kg/m^2^), mean (SD): • Intervention: 36.1 (6.4) • Control: 36.3 (6.5)	Intervention name: Noom Weight Intervention content: self-monitoring and feedback on food, exercise, weight; access to coach and support group; education on nutrition, activity, and behavioural change; provision of electronic scales and FitBit Theoretical underpinning: Cognitive Behavioural Therapy; Acceptance and Commitment Therapy; Dialectical Behaviour Therapy	Mode of delivery: app Duration: 4 months Frequency: continuous access	Standard care/control/comparator content: standard in-person counselling; provision of electronic scales and FitBit	**2.2** Feedback on behaviour **2.3** Self-monitoring of behaviour **2.4** Self-monitoring of outcome(s) of behaviour **2.7** Feedback on outcome(s) of behaviour **11.2** Reduce negative emotions	4-month follow-up: Mean weight (kg) • **Intervention: -8.5** • Control: +0.4 • **Between groups**	4-month follow-up: Mean ALT (IU/L) • Intervention: -9.2 • Control: -5.2 • Between groups Mean AST (IU/L) • Intervention: -4.3 • Control: -3.9 • Between groups Mean ALP (IU/L) • Intervention: +5.5 • Control: +11.3 • Between groups
**Tincopa *et al.* (2022)**^42^ USA Single-group pre–post study	Total sample: 40 • Intervention: 40 enrolled; 33 analysed Age (years), median (IQR): • Intervention: 53 (43–61) Female, n (%): • Intervention: 16 (48.5%) BMI (kg/m^2^), median (IQR): • Intervention: 33.6 (29.6–35.3)	Intervention name: No name reported Intervention content: Provision of FitBit; self-management of physical activity; feedback on physical activity with personalised step goals; motivational messages via email Theoretical underpinning	Mode of delivery: app and email Duration: 6 months Frequency: continuous access to app; email communication no more than once weekly for first 3 months and then bi-weekly for the remaining 3 months	Standard care/control/comparator content: N/A	**2.2** Feedback on behaviour **2.3** Self-monitoring of behaviour **3.1** Social support (unspecified) **7.1** Prompts/cues **12.5** Adding objects to the environment	6-month follow-up: Median weight (lbs) • Intervention: +7 Median HbA_1c_ (mmol/L) • **Intervention: -0.1** Median HDL (mg/dl) • **Intervention: +5.5** Median LDL (mg/dl) • **Intervention: -19.5** Median TGs (mg/dl) **Intervention: -10.5**	6-month follow-up: Median ALT (IU/L) • Intervention: -11 Median fibrosis: LSM (kPa) • Intervention: +0.6 Mean steatosis: CAP (dB/m) • Intervention: -22.5

∗ Change calculated by subtracting the mean/median value at follow-up from the mean/median value at baseline. Statistically significant (*p* <0.05) results are highlighted in bold. ALP, alkaline phosphatase; ALT, alanine transaminase; AST, aspartate transaminase; BCT, behaviour change technique; CAP, controlled attenuation parameter; GGT, gamma-glutamyl transaminase; HDL, high-density lipoprotein cholesterol; LDL, low-density lipoprotein cholesterol; LSM, liver stiffness measurement; MRI-PDFF, magnetic resonance imaging proton density fat fraction; RCT, randomised controlled trial; TG, triglyceride.

**Fig. 3:**
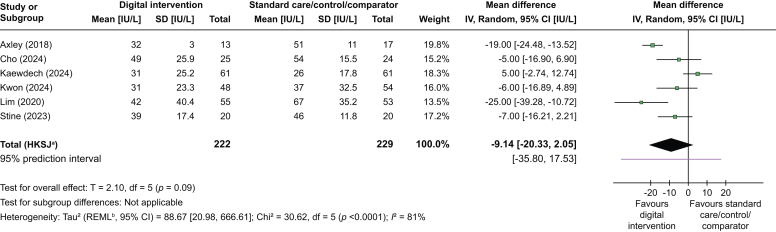
Random-effects meta-analysis for ALT (IU/L). ALT, alanine transaminase.

### Change in weight (kg, lbs, or %)

Digital interventions (compared with standard care/control/comparator) showed no statistically significant improvements in weight (weighted mean difference [WMD] -2.07 kg, 95% CI -6.08 to +1.94 kg, *I*^2^ = 90%) based on data from six RCTs^32^^,^[34], [35], [36], [37]^,^^41^ (Fig. 2).

**Fig. 4:**
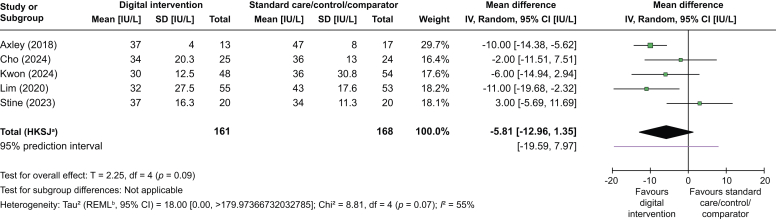
Random-effects meta-analysis for AST (IU/L). AST, aspartate aminotransferase.

Of the remaining five intervention studies (four single-group pre–post studies^33^^,^^39^^,^^40^^,^^42^ and one non-RCT^38^), two studies reported statistically significant post-intervention weight loss of -7.3 kg (*p* <0.001)^40^ and -3.5 kg (*p* <0.001).^33^ One study^38^ reported statistically significant reductions in body weight of -3.4% at 6-month follow-up, -4.9% at 12-month follow-up, and -5.5% at 24-month follow-up (all *p* <0.001). The remaining two studies reported changes in body weight of -5.1%^39^ and +7 lbs,^42^ but neither at a statistically significant level.

### Change in liver-related measures

#### ALT

Digital behavior change interventions, when compared with standard care/control/comparator, showed no statistically significant improvements in ALT (WMD -9.14 IU/L, 95% CI -20.33 to +2.05 IU/L, *I*^2^ = 81%) based on data from six RCTs^32^^,^[34], [35], [36], [37]^,^^41^ (Fig. 3).

The five intervention studies not included in the meta-analysis (four single-group pre–post studies^33^^,^^39^^,^^40^^,^^42^ and one non-RCT^38^) also assessed ALT as an outcome. Changes in ALT ranging from -49.5 to +1.8 IU/L were observed across studies (mean -20.9 IU/L, SD 15.62 IU/L). However, statistically significant changes were reported in only one study^40^ (reduction of -49.5 IU/L at post-intervention follow-up; *p* <0.001).

#### AST

Digital interventions (compared with standard care/control/comparator) showed no statistically significant improvements in AST (WMD -5.81 IU/L, 95% CI -12.96 to +1.35 IU/L, *I*^2^ = 55%) based on data from five RCTs.^32^^,^^34^^,^^36^^,^^37^^,^^41^ (Fig. 4).

Three single-group pre–post studies^33^^,^^39^^,^^40^ assessed AST as an outcome. Changes ranged from -23.1 to +1.5 IU/L (mean -12.2 IU/L, SD 9.53 IU/L), although these changes were only statistically significant for one study^40^ (reduction of -18.8 IU/L at post-intervention follow-up; *p* = 0.003).

#### Other liver function tests (GGT, ALP, and albumin)

Four studies (two RCTs,^34^^,^^36^ one single-group pre–post study,^40^ and one non-RCT^38^) assessed GGT as an outcome. Three studies reported a statistically significant improvement in GGT within intervention groups, with a mean improvement of -21.28 IU/L (SD 6.61 IU/L). One RCT^34^ reported a statistically significant reduction of -27.76 IU/L (*p* = 0.007) from baseline to post-intervention follow-up in the intervention group and a statistically significant difference between groups (*p* = 0.014). The second RCT^36^ reported a statistically significant reduction of -12.2 IU/L within the intervention group (*p* = 0.04), but this did not significantly differ from the control group. The single-group pre–post study^40^ reported a statistically significant reduction of -28.6 IU/L (*p* = 0.02) from baseline to post-intervention follow-up. The non-RCT^38^ narratively reported significant improvements in GGT from baseline to post-intervention follow-up and between groups.

Three studies (two RCTs^34^^,^^41^ and one single-group pre–post study^40^) assessed ALP as an outcome. The single-group pre–post study^40^ reported statistically significant reductions of -10.6 IU/L (*p* <0.001) from baseline to post-intervention follow-up. Of the two RCTs,^34^^,^^41^ neither reported statistically significant within- or between-group differences.

One RCT^34^ assessed albumin as an outcome. No statistically significant within-group improvements were observed in the intervention group from baseline to post-intervention follow-up, although there was a statistically significant difference between groups (*p* = 0.017).

#### Fibrosis (LSM; kPa), steatosis (CAP; dB/m), and liver fat percentage (MRI-PDFF)

Three studies (two single-group pre–post studies^33^^,^^42^ and one RCT^35^) assessed fibrosis (LSM; kPa) and steatosis (CAP; dB/m) as outcomes. The RCT^35^ reported a statistically significant reduction in LSM (-0.4 kPa) from baseline to post-intervention follow-up in the intervention group (*p* = 0.003), and when compared with the standard care group (*p* = 0.035). Both single-group pre–post studies^33^^,^^42^ reported no statistically significant changes in LSM from baseline to post-intervention follow-up. The RCT also reported a statistically significant reduction in CAP from baseline to post-intervention follow-up of -20.2 dB/m (*p* <0.001) in the intervention group, but no between-group differences were reported (*p* = 0.655).^35^ One single-group pre–post study^33^ reported a statistically significant reduction in CAP of -33.3 dB/m (*p* <0.001) from baseline to post-intervention follow-up. The second pre–post study^42^ reported no statistically significant changes.

Two single-group pre–post studies assessed fat percentage (MRI-PDFF) as an outcome.^33^^,^^39^ The first study reported a statistically significant reduction of -2.2% in fat percentage (*p* <0.001).^33^ No statistically significant differences from baseline to post-intervention follow-up were reported for the second study.^39^

### Change in metabolic outcomes

#### HbA_1c_ (mmol/L or %)

Four single-group pre–post studies assessed HbA_1c_ as an outcome.^33^^,^^39^^,^^40^^,^^42^ Of these studies, two reported statistically significant reductions of -0.5 mmol/L (*p* = 0.03)^33^ and -0.1 mmol/L (*p* <0.01)^42^ from baseline to post-intervention follow-up. Two studies reported no statistically significant changes.^39^^,^^40^

#### Lipid profile (TGs, LDL, HDL, and total cholesterol)

Six studies (three single-group pre–post studies,^33^^,^^40^^,^^42^ two RCTs,^32^^,^^34^ and one two-group non-RCT^38^) assessed TGs as an outcome. Four studies reported a statistically significant improvement in TGs within intervention groups, with a mean reduction of -12.7 mg/dl (SD 6.28 mg/dl).^32^^,^^33^^,^^40^^,^^42^ Of these four studies, the three single-group pre–post studies showed statistically significant reductions of -0.2 mmol/L (*p* = 0.003),^33^ -10.5 mg/dl (*p* = 0.03),^42^ and -19.9 mg/dl (*p* = 0.01)^40^ from baseline to post-intervention follow-up, whereas the RCT^32^ showed a statistically significant reduction of -17 mg/dl (*p* = 0.04) in the intervention group but found no differences between groups. The second RCT^34^ reported no statistically significant differences within or between groups. The two-group non-RCT^38^ showed no statistically significant differences between groups at any time point.

Four studies (three single-group pre–post studies^33^^,^^40^^,^^42^ and one RCT^34^) reported LDL as an outcome. One study^42^ reported a statistically significant reduction of -19.5 mg/dl (*p* <0.01), whereas the remaining three studies^33^^,^^34^^,^^40^ showed no statistically significant changes from baseline to post-intervention follow-up, or between intervention and control groups.

Similarly, four studies (two single-group pre–post studies^33^^,^^42^ and two RCTs^32^^,^^34^) assessed HDL as an outcome. One study showed a statistically significant increase of +5.5 mg/dl from baseline to post-intervention follow-up (*p* <0.01),^42^ whereas no statistically significant changes were observed in the remaining three studies [32], [33], [34].

Two studies (one single-group pre–post study^33^ and one RCT^34^) assessed total cholesterol (mmol/L) as an outcome. Neither reported statistically significant changes from baseline to post-intervention follow-up or between intervention and control groups.

### Change in lifestyle behaviours

Five studies (three single-group pre–post studies,^33^^,^^39^^,^^42^ one RCT,^35^ and one non-RCT^38^) assessed whether changes in physical activity/physical fitness were observed following engagement with the digital intervention. Two studies (one RCT,^35^ and one non-RCT^38^) assessed metabolic equivalents (METs) as an outcome. Both studies reported no statistically significant changes in METs from baseline to post-intervention follow-up or between groups. Two single-group studies^33^^,^^42^ assessed steps/day as an outcome. One study reported a statistically significant increase of +1,579 steps/day from pre-to post-intervention (*p* = 0.02),^33^ whereas the other reported a decrease of -751 steps/day that was not statistically significant.^42^ One single-group study^39^ reported an increase of +9.9 ml/kg/min in peak oxygen uptake (VO_2_ peak), although this was not assessed for statistical significance. Another single-group study^42^ assessed the 6-minute walk test as an outcome and reported a statistically significant increase of 140 feet from baseline to post-intervention follow-up (*p* <0.01). Only one study (a non-RCT^38^) assessed whether changes in dietary behaviours were observed after engagement with the intervention. This study assessed caloric intake (kcal/day) and reported a decrease in kcal/day in the intervention group (-273 kcal/day) and the comparator group (-193 kcal/day), but these were not statistically significant.

### Active intervention ingredients and intervention promise

Overall, 22 distinct BCTs were identified across all digital interventions (see Table 2). Each intervention included at least one BCT, and the mean number of BCTs used within interventions was 5 (SD 3.6; median 5; IQR 3-7; range 1–13). The most frequently coded BCTs were ‘social support (unspecified)’ (n = 8), ‘feedback on behaviour’ (n = 7), ‘self-monitoring of behaviour’ (n = 6), and ‘prompts/cues’ (n = 6). Table 2 presents the specific BCTs coded within each intervention in accordance with a valid and reliable behaviour change taxonomy.^24^

**Table 2:** BCTs coded within each digital intervention using BCTTv1.

BCTs (BCT taxonomy identifiers and name)	Axley *et al.* (2018)^32^	Björnddottir *et al.* (2024)^33^	Cho *et al.* (2024)^34^	Kaewdech *et al.* (2024)^35^	Kwon *et al.* (2024)^36^	Lim *et al.* (2020)^37^	Mazzotti *et al.* (2018)^38^	Motz *et al.* (2021)^39^	Sato *et al.* (2023)^40^	Stine *et al.* (2023)^41^	Tincopa *et al.* (2022)^42^
1.1 Goal setting (behaviour)			X		X						
1.2 Problem solving	X										
1.3 Goal setting (outcome)	X				X						
1.4 Action planning			X		X						
1.5 Review behaviour goal(s)					X						
1.7 Review outcome goal(s)					X						
2.1 Monitoring of behaviour by others without feedback							X				
2.2 Feedback on behaviour		X	X		X	X		X		X	X
2.3 Self-monitoring of behaviour		X	X		X	X				X	X
2.4 Self-monitoring of outcome(s) of behaviour			X						X	X	
2.6 Biofeedback								X			
2.7 Feedback on outcome(s) of behaviour			X			X				X	
3.1 Social support (unspecified)	X	X	X		X	X		X	X		X
4.1 Instruction on how to perform the behaviour					X				X		
6.1 Demonstration of the behaviour					X						
7.1 Prompts/cues	X		X	X	X	X					X
8.3 Habit formation			X								
8.4 Habit reversal			X								
9.1 Credible source					X	X		X			
10.4 Social reward					X	X					
11.2 Reduce negative emotions										X	
12.5 Adding objects to the environment			X					X			X

BCT, behaviour change techniques; BCTTv1, Behaviour Change Techniques Taxonomy version 1.

Table 3 presents promise ratios for the active ingredients (intervention features and BCTs) of the included interventions that are associated with improvements in weight/metabolic outcomes and/or liver-related outcome measures.

**Table 3:** Promise ratios for active ingredients of digital behavioural interventions.

Active ingredient of digital behavioural interventions	Associated with improvement in weight/metabolic outcomes				Associated with improvement in liver-related outcome measures			
	n	Presence in very/quite promising interventions	Presence in non-promising interventions	Promise ratio	n	Presence in very/quite promising interventions	Presence in non-promising interventions	Promise ratio
**Mode of delivery**								
App	5	**4**	**1**	**4.00**	5	**4**	**1**	**4.00**
SMS/instant messaging	2	1	1	1.00	2	**2**	-	–
App and additional communication (*i.e.* email/SMS)	2	**2**	-	–	2	1	1	1.00

**Duration of intervention (months)**								
<6	5	3	2	1.50	5	3	2	1.50
≥6	6	**5**	**1**	**5.00**	6	**5**	**1**	**5.00**

**Intervention frequency**								
Continuous access	4	**4**	-	–	4	**3**	**1**	**3.00**
3–7 times/week	3	1	2	0.50	3	**2**	**1**	**2.00**
Continuous access with additional communication 1–7 times/week	2	**2**	-	-	2	1	1	1.00
Continuous access with provision of exercise session/meal replacement 1–7 times/week	2	1	1	1.00	2	**2**	-	–

**BCTs∗**								
1.1 Goal setting (behaviour)	2	1	1	1.00	2	**2**	-	–
1.3 Goal setting (outcome)	2	**2**	-	–	2	**2**	-	–
1.4 Action planning	2	1	1	1.00	2	**2**	-	–
2.2 Feedback on behaviour	7	**5**	**2**	**2.50**	7	4	3	1.33
2.3 Self-monitoring of behaviour	6	**5**	**1**	**5.00**	6	**4**	**2**	**2.00**
2.4 Self-monitoring of outcome(s) of behaviour	3	**2**	**1**	**2.00**	3	**2**	**1**	**2.00**
2.7 Feedback on outcome(s) of behaviour	3	**2**	**1**	**2.00**	3	**2**	**1**	**2.00**
3.1 Social support (unspecified)	8	**6**	**2**	**3.00**	8	**6**	**2**	**3.00**
4.1 Instruction on how to perform the behaviour	2	**2**	-	–	2	**2**	-	–
7.1 Prompts/cues	6	**4**	**2**	**2.00**	6	**5**	**1**	**5.00**
9.1 Credible source	3	**2**	**1**	**2.00**	3	**2**	**1**	**2.00**
10.4 Social reward	2	**2**	-	–	2	**2**	-	–
12.5 Adding objects to the environment	3	1	2	0.50	3	1	2	0.50

Promise ratio denotes the number of very or quite promising interventions in which an intervention ingredient featured, divided by the number of non-promising interventions in which it featured. Promise ratios are only calculable for ingredients used in both promising and non-promising interventions. Where functions or techniques were used only in (two or more) promising interventions (promise ratio = ∞), the number of interventions in which they were used was reported instead of the ratio. Ratios in bold denote ingredients associated with a promise ratio of 2 or above, or used exclusively in promising interventions and featuring in at least two interventions.^43^ ∗Number reported with BCT corresponds to the identifying number reported in the BCTTv1.^24^ BCT, behaviour change technique; BCTTv1, Behaviour Change Techniques Taxonomy version 1.

App-based delivery was the only mode of delivery that was associated with improvements in both weight/metabolic outcomes and liver-related outcome measures (promise ratios 4.00). In relation to improvements in liver-related outcome measures, SMS/instant messaging was used in promising interventions only (n = 2). However, SMS/instant messaging was not associated with improvements in weight/metabolic outcomes. Similarly, in relation to improvements in weight/metabolic outcomes, app-based delivery in conjunction with another mode of communication (*i.e.* e-mail or SMS) was used in promising interventions only (n = 2) but was not associated with improvements in liver-related outcome measures.

An intervention duration of ≥6 months was associated with improvements in both weight/metabolic outcomes and liver-related outcome measures (promise ratios 5.00), whereas an intervention duration of <6 months was not associated with improvements in either outcome. Continuous access was associated with improvements in liver-related outcomes (promise ratio 3.00). Similarly, in relation to improvements in weight/metabolic outcomes, continuous access to intervention content was used in promising interventions only (n = 4). A frequency of access three to seven times per week was also associated with improvements in liver-related outcome measures (promise ratio 2.00). Continuous access with provision of an exercise session and/or meal replacement one to seven times per week was not associated with improvements in weight/metabolic outcomes. In relation to improvements in liver-related outcome measures, continuous access with provision of an exercise session and/or meal replacement one to seven times per week was present in promising interventions only (n = 2).

Of the 22 BCTs identified across all interventions, 13 were used by at least two interventions. Seven BCTs were associated with improvements in weight/metabolic outcomes, and six BCTs were associated with improvements in liver-related outcomes. Examples of BCTs associated with improvements in weight/metabolic outcomes include ‘self-monitoring of behaviour’ (promise ratio 5.00), ‘social support (unspecified)’ (promise ratio 3.00), and ‘feedback on behaviour’ (promise ratio 2.50). Examples of BCTs associated with improvements in liver-related outcome measures were ‘prompts/cues’ (promise ratio 5.00) and ‘social support (unspecified)’ (promise ratio 3.00). Overall, six BCTs were associated with improvements in both weight/metabolic outcomes and liver-related outcome measures. They were ‘self-monitoring of behaviour’, 'self-monitoring of outcome(s) of behaviour', ‘feedback on outcome(s) of behaviour', ‘social support (unspecified)’, ‘prompts/cues’, and ‘credible source’.

### Methodological quality assessment

A summary of methodological quality assessment for all 11 included studies is presented in Supplementary file 7 in accordance with the assessment tool used.

Six studies[32], [34], [35], [36], [37], [41] were assessed using the ROB-2 tool.^25^ These studies were rated as having ‘some concerns’ because of a lack of information about pre-specified analysis plans. One study^38^ was assessed using the ROBINS-I tool^26^ and was rated as ‘low’ risk of bias. Four studies^33^^,^^39^^,^^40^^,^^42^ were assessed using the NHLBI pre–post tool.^27^ Of these, one study^40^ was rated as ‘good’ quality. Two studies^33^^,^^42^ were rated as ‘fair’ because of a lack of reporting concerning whether the sample size was sufficiently large to provide confidence in the findings. One study^39^ was rated as ‘poor’ quality because of a lack of clarity about the reporting of study objectives and a small sample size.

## Discussion

This is the first systematic review and meta-analysis to determine the effectiveness and active ingredients of digital behaviour change interventions for improving weight and liver-related outcome measures in patients diagnosed with MASLD.

A total of 11 studies reporting on digital behaviour change interventions met the eligibility criteria. Findings of the meta-analysis involving five RCTs for AST and six RCTs for ALT and weight indicate that, overall, digital behavior change interventions are not effective for improving weight or liver-related outcome measures. Findings from pre–post studies and one non-RCT showed statistically significant improvements in weight (n = 3 studies) and improvements in weight that were not statistically significant (n = 1 study). Findings from pre–post and non-RCT studies showed statistically significant improvements in ALT (n = 1 study), improvements in ALT that were not statistically significant (n = 3 studies), and worsening of ALT that was not statistically significant (n = 1 study). Similarly, findings from pre–post and non-RCT studies showed statistically significant improvements in AST (n = 1 study), improvements in AST that were not statistically significant (n = 1 study), and worsening of AST that was not statistically significant (n = 1 study). Promise analysis conducted on data from studies that reported statistically significant findings in relation to weight/metabolic outcomes and/or liver-related outcome measures identified specific features and content of digital interventions that likely moderated the effectiveness of the interventions. The findings of this current review do not support the findings of a previous review published in 2024 that reported digital interventions as effective for weight loss.^21^ However, the meta-analysis presented in the 2024 systematic review included data from single-group studies and intervention arms of RCTs only. This likely contributed to the larger effect sizes reported compared with the current review.

All studies included in the current review differed in terms of their mode of delivery, duration, frequency of intervention delivery, and specific BCTs included within them. App-based delivery and an intervention duration of ≥6 months were both associated with improvements in weight/metabolic outcomes and liver-related outcome measures. Similarly, six distinct BCTs were identified as promising for improving weight/metabolic outcomes and liver-related outcome measures. Of the six BCTs that showed promise, many are associated with self-regulation of behaviours, including diet and physical activity (*i.e.* ‘self-monitoring of behaviour’ and ‘prompts/cues’). The identification of these BCTs emphasises the important role that self-regulation plays in improving MASLD-related outcomes. This finding supports several health behaviour change theories that position self-regulation as a central mechanism of action in health behaviour change.^44^^,^^45^ Of the 11 studies included in this review, only two reported the use of theory in intervention development.^37^^,^^41^ Theory provides a structured and explicit framework for designing, evaluating, and optimising interventions. This can inform intervention content, enhance effectiveness by ensuring interventions target appropriate behavioural determinants, provide a framework for evaluation, and facilitate replication. A substantial body of evidence has highlighted the effectiveness of theory-based interventions for targeting change in behaviour^46^^,^^47^ The Medical Research Council framework for the Development and Evaluation of Complex Interventions suggests that complex interventions should be developed with reference to theory and systematically following phases of iterative development (*i.e.* considering core elements of the intervention, assessment of feasibility, consideration of implementation, and evaluation).^48^ Digital behaviour change interventions are often complex and multi-faceted; therefore, it is recommended that they follow a systematic, theory-informed development process, taking into account the complexity of the intended intervention.^49^

Several systematic reviews have been conducted to determine the efficacy of digital behaviour change interventions for other long-term health conditions (*e.g.* type 2 diabetes) and have reported BCTs or combinations of BCTs (*i.e.* social support and feedback on behaviour) positively associated with improvements in clinical outcome measures, similar to those reported by this review.^50^^,^^51^ The findings of the current review are important as they highlight the specific active ingredients that could be central to the management of MASLD and for improving MASLD-related outcomes in future research or clinical practice. The success of specific BCTs (and other intervention features, including mode of digital intervention delivery, frequency of delivery, and duration of provision) for improving clinical outcome measures in other conditions further emphasises the utility of incorporating promising active ingredients into interventions to facilitate improvements in MASLD-related outcomes.

Importantly, only five of the studies included in this systematic review explicitly measured and reported data on changes in lifestyle behaviours.^33^^,^^35^^,^^38^^,^^39^^,^^42^ This is despite most interventions aiming to change lifestyle behaviours (*i.e.* diet and physical activity) to positively impact weight or liver-related outcome measures. Therefore, it cannot be definitively concluded that changes in weight and liver-related outcome measures were a consequence of any changes in lifestyle behaviours targeted by the intervention. Future studies should explicitly measure whether the intervention leads to changes in lifestyle behaviours that precede changes in weight and liver-related outcome measures.

### Strengths and limitations

A strength of this review is the use of robust methodology and data analysis to determine the effectiveness of digital behaviour change interventions for improving weight and liver-related outcome measures and to identify promising intervention features and content. This review is the first of its kind to conduct these analyses in the context of MASLD and provides important information to inform the development and optimisation of future digital interventions. Although it was not possible to conduct a meta-analysis of all included studies owing to study design and heterogeneity of outcomes reported, an alternative approach was used (i.e., SWiM) to ensure that data of single-group studies and non-RCTs were examined consistently, robustly, and transparently.

A potential limitation of this review is the low number of RCTs included, and those were largely feasibility and pilot studies with small sample sizes. This indicates the relative infancy of this field, which demonstrates the need for large, controlled studies to establish a more robust evidence base. Similarly, several studies included were rated as having ‘some concerns’ following risk of bias assessment. This may have implications for the reliability of the conclusions drawn.

### Conclusions

The findings of this systematic review indicate that digital behaviour change interventions, overall, are not effective for improving weight and liver-related outcome measures in patients with MASLD. However, of those individual studies that did report statistically significant improvements in weight/metabolic outcomes and liver-related outcome measures, we were able to identify a range of promising active intervention ingredients, which provide some guidance for the development of future digital behaviour change interventions or the optimisation of existing interventions. It is important to refer to intervention features and content that have been shown to be associated with effectiveness in other intervention studies, in related clinical fields, and in this review. In conclusion, although the findings of this review indicate that digital behaviour change interventions are not effective for improving weight and liver-related outcomes measures overall, it is clear that these interventions do work for some people, and it is important to identify who those people are. Moreover, findings indicate that those interventions that do lead to effective changes in weight and/or liver-related outcome measures share some common features and content. These findings can inform future digital behaviour change interventions for MASLD.

## Abbreviations

ALP, alkaline phosphatase; ALT, alanine transaminase; AST, aspartate transaminase; BCT, behaviour change technique; BCTTv1, Behaviour Change Techniques Taxonomy version 1; CAP, controlled attenuation parameter; DPP, Diabetes Prevention Programme; GGT, gamma-glutamyl transaminase; HCP, healthcare professional; HDL, high-density lipoprotein cholesterol; LDL, low-density lipoprotein cholesterol; LSM, liver stiffness measurement; MASH, metabolic dysfunction-associated steatohepatitis; MASLD, metabolic dysfunction-associated steatotic liver disease; METS, metabolic equivalents; MRI-PDFF, magnetic resonance imaging proton density fat fraction; NHLBI, National Heart, Lung and Blood Institute; NHS, National Health Service; PRISMA, Preferred Reporting Items for Systematic reviews and Meta-Analyses; RCT, randomised controlled trial; ROB-2, Revised Cochrane Risk of Bias Tool; ROBINS-I, Risk Of Bias In Non-randomised Studies of Interventions; SWiM, Synthesis Without Meta-Analysis; TG, triglyceride; WMD, weighted mean difference.

## Financial support

This systematic review was conducted as part of a Teesside University fully funded PhD studentship undertaken by HS.

## Authors’ contributions

Conceived the idea for this review: HS, LA, SMc, KH. Developed the review protocol: HS, LA, SMc, KH, AI. Developed the search strategy and conducted database searching: HS. Conducted stage 1 and stage 2 screening: HS, RL. Conducted data extraction, with input from all authors when required: HS. Coded the presence of behaviour change techniques: HS, KA. Conducted methodological quality assessment: HS, LA, KA, MC. Meta-analysed/synthesised data: HS. Provided support in meta-analysis/synthesis of data: LA, KH, SMc. Drafted the manuscript: HS. Revised the manuscript for important intellectual content and approved the final version: all authors.

## Data availability

The data set from this systematic review and meta-analysis is available upon reasonable request.

## Conflicts of interest

SMc has received consultancy/speakers fees from Abbvie, Allergan, BMS, Gilead, Intercept, MSD, Novo Nordisk, Norgine, Novartis, and Sequana. The remaining authors declare no conflicts of interest relating to this work.

Please refer to the accompanying ICMJE disclosure forms for further details.

## Footnotes

Appendix A Supplementary data to this article can be found online at https://doi.org/10.1016/j.jhepr.2025.101507.
