# Supplementary material for: Effectiveness and active ingredients of digital behaviour change interventions for MASLD: A systematic review and meta-analysis
Source: JHEP Rep. 2025 Jul 2;7(10):101507. doi: 10.1016/j.jhepr.2025.101507 (PMC12448008; doi:10.1016/j.jhepr.2025.101507)
Supplement: Multimedia component 1 [file mmc1.pdf]

Effectiveness and active ingredients of digital behaviour change interventions for patients with MASLD: A systematic review and meta-analysis

Hollie Smith, Rebecca Livingston, Kirsten Ashley, Matthew Cooper, Stuart McPherson, Alison Innerd, Kate Hallsworth, Leah Avery

Table of contents

S1. PRISMA checklists……………………………………………………….…….....2

S2. Search strategies………………………………………………………………….5

S3. Study selection form…………………………………………………………….7

S4. Data extraction form……………………………………………………………8

S5. SWIM checklist………………………………………………………………..22

S6. Outcome metrics……………………………………………………………...24

S7. Risk of bias assessments……………………………………………….....27

Supplementary file 1. PRISMA checklists.

Section and Topic	Item #	Checklist item	Location where item is reported
**TITLE**			
Title	1	Identify the report as a systematic review.	Title
**INTRODUCTION**			
Rationale	3	Describe the rationale for the review in the context of existing knowledge.	Intro (p6-8)
Objectives	4	Provide an explicit statement of the objective(s) or question(s) the review addresses.	Intro (p8)
**METHODS**			
Eligibility criteria	5	Specify the inclusion and exclusion criteria for the review and how studies were grouped for the syntheses.	Eligibility criteria (p9); data synthesis (p11)
Information sources	6	Specify all databases, registers, websites, organisations, reference lists and other sources searched or consulted to identify studies. Specify the date when each source was last searched or consulted.	Search strategy (p8-9)
Search strategy	7	Present the full search strategies for all databases, registers and websites, including any filters and limits used.	S2
Selection process	8	Specify the methods used to decide whether a study met the inclusion criteria of the review, including how many reviewers screened each record and each report retrieved, whether they worked independently, and if applicable, details of automation tools used in the process.	Study selection (p8-9)
Data collection process	9	Specify the methods used to collect data from reports, including how many reviewers collected data from each report, whether they worked independently, any processes for obtaining or confirming data from study investigators, and if applicable, details of automation tools used in the process.	Data extraction (p10)
Data items	10a	List and define all outcomes for which data were sought. Specify whether all results that were compatible with each outcome domain in each study were sought (e.g. for all measures, time points, analyses), and if not, the methods used to decide which results to collect.	Eligibility criteria (p9-10)
	10b	List and define all other variables for which data were sought (e.g. participant and intervention characteristics, funding sources). Describe any assumptions made about any missing or unclear information.	Data extraction (p10)
Study risk of bias assessment	11	Specify the methods used to assess risk of bias in the included studies, including details of the tool(s) used, how many reviewers assessed each study and whether they worked independently, and if applicable, details of automation tools used in the process.	Methodological quality assessment (p11)
Effect measures	12	Specify for each outcome the effect measure(s) (e.g. risk ratio, mean difference) used in the synthesis or presentation of results.	Data synthesis (p11)
Synthesis methods	13a	Describe the processes used to decide which studies were eligible for each synthesis (e.g. tabulating the study intervention characteristics and comparing against the planned groups for each synthesis (item #5)).	Data synthesis (p11)
	13b	Describe any methods required to prepare the data for presentation or synthesis, such as handling of missing summary statistics, or data conversions.	N/A

Section and Topic	Item #	Checklist item	Location where item is reported
	13c	Describe any methods used to tabulate or visually display results of individual studies and syntheses.	Table 1
	13d	Describe any methods used to synthesize results and provide a rationale for the choice(s). If meta-analysis was performed, describe the model(s), method(s) to identify the presence and extent of statistical heterogeneity, and software package(s) used.	Data synthesis (p11)
	13e	Describe any methods used to explore possible causes of heterogeneity among study results (e.g. subgroup analysis, meta-regression).	Data synthesis (p11)
	13f	Describe any sensitivity analyses conducted to assess robustness of the synthesized results.	N/A
Reporting bias assessment	14	Describe any methods used to assess risk of bias due to missing results in a synthesis (arising from reporting biases).	Methodological quality assessment (p11)
Certainty assessment	15	Describe any methods used to assess certainty (or confidence) in the body of evidence for an outcome.	Promise of intervention content and features (p11-12)
**RESULTS**			
Study selection	16a	Describe the results of the search and selection process, from the number of records identified in the search to the number of studies included in the review, ideally using a flow diagram.	Figure 1
	16b	Cite studies that might appear to meet the inclusion criteria, but which were excluded, and explain why they were excluded.	Figure 1
Study characteristics	17	Cite each included study and present its characteristics.	Table 1
Risk of bias in studies	18	Present assessments of risk of bias for each included study.	Methodological quality assessment (p21)
Results of individual studies	19	For all outcomes, present, for each study: (a) summary statistics for each group (where appropriate) and (b) an effect estimate and its precision (e.g. confidence/credible interval), ideally using structured tables or plots.	Figure 2 & Figure 3
Results of syntheses	20a	For each synthesis, briefly summarise the characteristics and risk of bias among contributing studies.	Figure 2 & Figure 3
	20b	Present results of all statistical syntheses conducted. If meta-analysis was done, present for each the summary estimate and its precision (e.g. confidence/credible interval) and measures of statistical heterogeneity. If comparing groups, describe the direction of the effect.	Weight (p13), ALT (p16), AST (p16)
	20c	Present results of all investigations of possible causes of heterogeneity among study results.	N/A
	20d	Present results of all sensitivity analyses conducted to assess the robustness of the synthesized results.	N/A
Reporting biases	21	Present assessments of risk of bias due to missing results (arising from reporting biases) for each synthesis assessed.	Methodological quality assessment

Section and Topic	Item #	Checklist item	Location where item is reported
			(p11)
Certainty of evidence	22	Present assessments of certainty (or confidence) in the body of evidence for each outcome assessed.	Active intervention ingredients (p18-21)
**DISCUSSION**			
Discussion	23a	Provide a general interpretation of the results in the context of other evidence.	Discussion (p21-25)
	23b	Discuss any limitations of the evidence included in the review.	Strengths and limitations (p25)
	23c	Discuss any limitations of the review processes used.	Strengths and limitations (p25)
	23d	Discuss implications of the results for practice, policy, and future research.	Conclusions (p25-26)
**OTHER INFORMATION**			
Registration and protocol	24a	Provide registration information for the review, including register name and registration number, or state that the review was not registered.	Search strategy (p8-9)
	24b	Indicate where the review protocol can be accessed, or state that a protocol was not prepared.	Search strategy (p8-9)
	24c	Describe and explain any amendments to information provided at registration or in the protocol.	Search strategy (p8-9)
Support	25	Describe sources of financial or non-financial support for the review, and the role of the funders or sponsors in the review.	Title page
Competing interests	26	Declare any competing interests of review authors.	Title page
Availability of data, code and other materials	27	Report which of the following are publicly available and where they can be found: template data collection forms; data extracted from included studies; data used for all analyses; analytic code; any other materials used in the review.	Supplementary files

From: Page MJ, McKenzie JE, Bossuyt PM, Boutron I, Hoffmann TC, Mulrow CD, et al. The PRISMA 2020 statement: an updated guideline for reporting systematic reviews. BMJ 2021;372:n71. doi: 10.1136/bmj.n71

For more information, visit: http://www.prisma-statement.org/

Supplementary file 2. Search strategies.

MEDLINE (EBSCO):

("non alcoholic fatty liver disease"[MeSH] OR “metabolic dysfunction-associated steatotic liver disease”[tiab] OR MASLD[tiab] OR NAFLD[tiab] OR MASH[tiab] OR NASH[tiab] OR “metabolic dysfunction-associated steatohepatitis”[tiab] OR "non alcoholic steatohepatitis"[tiab])

AND

("life style"[MeSH] OR "healthy eating"[tiab] OR diet[MeSH] OR "diet therapy"[MeSH] OR Exercise[MeSH] OR "Exercise Therapy"[MeSH] OR "physical activity"[tiab] OR "endurance training"[tiab] OR "strength training"[tiab] OR “resistance training”[tiab] OR "weight loss"[MeSH] OR "weight reduction"[tiab])

AND

(“telehealth” OR "tele health" OR “telemedicine” OR "tele medicine" OR "mobile health"[Title/Abstract:~3] OR “mhealth” OR "m health" OR “ehealth” OR "e health" OR "digital health"[Title/Abstract:~3] OR "digital therapeutics"[Title/Abstract:~3] OR "mobile phone" OR “smartphone” OR “wearable*”[tiab] OR "remote monitor"[Title/Abstract:~3] OR "remote monitoring"[Title/Abstract:~3] OR "mobile app"[Title/Abstract:~3] OR "mobile application"[Title/Abstract:~3] OR “web” OR “web-based” OR “text message” OR “self-management”)

CINAHL (EBSCO):

((MH "Nonalcoholic Fatty Liver Disease") OR (MH "Liver Diseases") OR (MH "Fatty Liver") OR "MASLD" OR (MH "Metabolic Diseases") OR "metabolic dysfunction-associated steatotic liver disease" OR "steatosis" OR "NASH") AND (“digital” OR “digital technology” OR “telehealth” OR “e−health” OR “ehealth” OR “tele−health” OR “mhealth” OR “mobile health units” OR “tele medicine” OR “telemedicine” OR “tele-medicine” OR “virtual medicine” OR “virtual” OR “app” OR “mobile” OR “mobile app” OR “mobile application” OR “mobile apps” OR “portable software app” OR “portable software application” OR “portable software applications” OR “portable software apps” OR “tablet application” OR “remote delivery” OR “text” OR “text message*” OR “SMS” OR “instant messag*” OR “phone” OR “cell” OR “cellphone” OR “cell-phone” OR “smartphone” OR “tablet” OR “tablet computer” OR “computer” OR “laptop” OR “PC” OR “email” OR “e-mail”) AND (“intervention” OR “digital N3 intervention*” OR “web-based intervention” OR “web intervention” OR “online intervention” OR “virtual intervention” OR “internet−based intervention” OR “internet−intervention” OR “online−based intervention” OR “online−intervention” OR “web intervention” OR “lifestyle intervention” OR “lifestyle modification” OR “lifestyle change”)

PsycINFO:

(DE “Liver Disorders” OR DE “Liver” OR “NAFLD” OR “NAFLD nonalcoholic fatty liver disease” OR “non alcoholic fatty liver disease” OR “non alcoholic hepato−steatosis” OR “non alcoholic hepatosteatosis” OR “non alcoholic liver steatosis” OR “non−alcoholic fatty liver” OR “nonalcoholic fatty liver” OR “nonalcoholic liver steatosis” OR “fatty liver disease” OR “hepatic steatosis” OR “hepatosteatosis” OR “liver steatosis”) AND (“digital” OR “digital technology” OR “telehealth” OR “e−health” OR “ehealth” OR “tele−health” OR “mhealth” OR “mobile health units” OR “tele medicine” OR “telemedicine” OR “tele-medicine” OR “virtual medicine” OR “virtual” OR “app” OR “mobile” OR “mobile app” OR “mobile application” OR “mobile apps” OR “portable software app” OR “portable software application” OR “portable software applications” OR “portable software

apps” OR “tablet application” OR “remote delivery” OR “text” OR “text message*” OR “SMS” OR “instant messag*” OR “phone” OR “cell” OR “cellphone” OR “cell-phone” OR “smartphone” OR “tablet” OR “tablet computer” OR “computer” OR “laptop” OR “PC” OR “email” OR “e-mail”) AND (“intervention” OR “digital N3 intervention*” OR “web-based intervention” OR “web intervention” OR “online intervention” OR “virtual intervention” OR “internet−based intervention” OR “internet−intervention” OR “online−based intervention” OR “online−intervention” OR “web intervention” OR “lifestyle intervention” OR “lifestyle modification” OR “lifestyle change”)

Web of Science:

((ALL=(NAFLD)) OR ALL=(fatty liver)) OR ALL=(steatosis)

AND

((((((((((((((((((ALL=(digital)) OR ALL=(app)) OR ALL=(web)) OR ALL=(ehealth)) OR ALL=(telehealth)) OR ALL=(virtual)) OR ALL=(phone)) OR ALL=(digital tech*)) OR ALL=(e-health)) OR ALL=(mhealth)) OR ALL=(telemedicine)) OR ALL=(tele medicine)) OR ALL=(tele-medicine)) OR ALL=(mobile)) OR ALL=(portable software)) OR ALL=(remote deliver*)) OR ALL=(text)) OR ALL=(internet)) OR ALL=(computer)) OR ALL=(email)

AND

(((ALL=(intervention)) OR ALL=(digital intervention)) OR ALL=(lifestyle intervention)) OR ALL=(lifestyle change)

Scopus:

(("fatty liver") OR ("steatosis")) AND (("digital") OR ("app") OR ("web") OR ("ehealth") OR ("telehealth") OR (“telemedicine”) OR (“tele-medicine”) OR ("virtual") OR ("phone") OR ("digital tech*") OR ("e-health") OR ("mhealth") OR ("telemedicine") OR ("mobile") OR ("portable software") OR ("remote deliver*") OR ("text") OR ("internet") OR ("computer") OR ("email")) AND (("intervention") OR ("digital intervention") OR ("lifestyle intervention") OR ("lifestyle change"))

Supplementary file 3. Study selection form.

Study selection form for assessing eligibility of full text papers.

**Study ID:**	
**Reviewer initials:**	
**Date:**	

**1. Participants**	**Yes/No/Unsure**
• Age 18+<br>• Diagnosis of MASLD/MASH with no other cause of liver disease (e.g. secondary causes of steatosis)	
**2. Intervention**	**Yes/No/U**
• Digital (e.g. web, app, SMS) lifestyle intervention program (e.g. diet, physical activity) for patients with MASLD<br>• NOT remote delivery of standard care i.e. a telephone consultation without a behavioural intervention component	
**3. Comparator/control**	**Yes/No/Unsure**
• No comparator/control<br>OR<br>• Usual/standard care in-person delivered behavioural interventions	
**4. Study design**	**Yes/No/Unsure**
• Any primary study design (e.g. RCT, pilot RCT, feasibility study) that includes pre- and post-intervention data OR intervention vs control data for the main outcomes	
**5. Main outcomes**	**Yes/No/Unsure**
Baseline and follow-up data concerning both:<br>• Metabolic outcomes (i.e. body weight, HbA1c)<br>• Liver outcomes (i.e. liver stiffness, histology)	

Decision* (Include/Exclude/Discuss)	
1st reason	

*To include: 1 and 2 and 3 and 4 and 5 must = YES

Supplementary file 4. Data extraction form.

Data Extraction Form

1. General Information

**Study ID**	
**Data extracted by:**	
**Date:**	
**Full bibliographic details:**	
**References of linked publications (e.g. protocol, intervention development, feasibility testing):**	
**Corresponding author contact details (where provided):**	

2. Study Characteristics

**Country of origin**	
**Aims and objectives**	
**Study design (Please circle)**	Definitive RCT<br>Pilot RCT<br>Qualitative study<br>Non-randomised controlled trial<br>Controlled before-and-after study

	Cohort Study<br>Case Control Study<br>Cross-sectional study<br>Mixed methods – please describe:<br>Other – please describe:		
**Number of study arms**			
**Inclusion and exclusion criteria**	Inclusion:<br>Exclusion:		
**Sampling method (Please circle)**	Convenience/Opportunistic<br>Volunteer<br>Snowball<br>Random<br>Stratified<br>Other – please describe:		
**Sample size (Number of patients consented and randomised)**	Total sample size (n=)	Intervention group (n=)	Control group (n=)
**Sample size based on power analysis/sample size estimation?**	Yes/No		
**Sample size (as indicated by power analysis/sample size**	Yes/No		

**estimation) achieved at final follow up?**	
**Intention to treat**	Yes/No
**Follow up time periods (e.g. baseline, 3 months etc.)**	
**Loss to follow up**<br><br>**(n, %) at each time period**	
**Conflicts of interest recorded**	

3. Participant Characteristics

	**Intervention group**	**Control group**
**Age**<br><br>(Mean/median/SD/IQR/range/not reported)		
**Gender**<br><br>(Frequency/percentage/not reported)		
**Ethnicity**<br><br>(Frequency/percentage/not reported)		

**BMI**<br>(Mean/median/SD/IQR/range/not reported)		
**Comorbidities**<br>(Mean/median/SD/IQR/range/not reported)		

4. Intervention Characteristics

**Name of intervention (e.g. VITALISE)**	
**Digital format of intervention (Please circle)**	Web-based<br>App-based<br>Text-messaging<br>Email<br>Video call<br>Other – Please describe:
**Location of delivery (Please circle)**	Primary care<br>Secondary care<br>Community/voluntary organisation<br>Other – Please describe:
**Who delivered the intervention?**	Primary care (GP)<br>Primary care (other HCP)<br>Secondary care (Consultant)

	Secondary care (other HCP)<br><br>Other – Please describe:
**Intervention frequency and duration (Duration of actual intervention delivery e.g. one 20 minute session; continuous access to intervention over 6 month period etc.)**	
**Intervention content (e.g. information with associated health coaching)**	
**Control content**	

5. Outcomes Assessed

	**Included?**<br><br>**(Yes/No)**	**Description**<br><br>**(Including details of data collection methods and/or scales used)**
Metabolic outcomes<br><br>(i.e. body weight, HbA1c, lipid profile measures)		
Liver outcomes (i.e. stiffness, histology)		
Lifestyle behaviour change (i.e. physical activity, dietary behaviours)		

6. Relevant Outcomes (Results/Findings)

Relevant outcome<br><br>(Please state)	Group	Baseline	Other follow up points	Final follow-up	Statistically significant difference between baseline and any follow up	Statistically significant difference between intervention and usual care groups at baseline and any follow-up
	Intervention					
	Control					
	Intervention					
	Control					
	Intervention					
	Control					
	Intervention					
	Control					

7. Development process

**Evidence of a systematic development process?**	**Was a framework used to inform intervention development?**	Yes/No/Not reported<br><br>Intervention Mapping<br>MRC Framework<br>Other – Please describe

	**Informed by evidence?**	Yes/No/Not reported
	**Informed by needs assessment with patients/relatives/stakeholders?**	Yes/No/Not reported
	**Evidence of 'testing' in patients/families/facilitators etc?**	Yes/No/Not reported
**Development underpinned by a theory/model of behaviour change, or by various constructs of a theory/model of behaviour change?**	Yes/No/Not reported<br><br>If yes, please describe:	
**Evidence of co-design/coproduction**	Yes/No/Not reported<br><br>If yes, please describe:	

8. Behaviour Change Techniques Used (BCTT v1) above and beyond control group

		Page(s) number where present	Comments
**1. Goals and planning**<br>1.1. Goal setting (behavior)<br>1.2. Problem solving<br>1.3. Goal setting (outcome)<br>1.4. Action planning<br>1.5. Review behavior goal(s)	Yes/No/Unsure		

1.6. Discrepancy between current behavior and goal<br>1.7. Review outcome goal(s)<br>1.8. Behavioral contract<br>1.9. Commitment			
**2. Feedback and monitoring**<br>2.1. Monitoring of behavior by others without feedback<br>2.2. Feedback on behaviour<br>2.3. Self-monitoring of behaviour<br>2.4. Self-monitoring of outcome(s) of behaviour<br>2.5. Monitoring of outcome(s) of behavior without feedback<br>2.6. Biofeedback<br>2.7. Feedback on outcome(s) of behavior	Yes/No/Unsure		
**3. Social support**<br>3.1. Social support (unspecified)<br>3.2. Social support (practical)<br>3.3. Social support (emotional)	Yes/No/Unsure		
**4. Shaping knowledge**	Yes/No/Unsure		

4.1. Instruction on how to perform the behavior<br>4.2. Information about antecedents<br>4.3. Re-attribution<br>4.4. Behavioral experiments			
**5. Natural consequences**<br>5.1. Information about health consequences<br>5.2. Salience of consequences<br>5.3. Information about social and environmental consequences<br>5.4. Monitoring of emotional consequences<br>5.5. Anticipated regret<br>5.6. Information about emotional consequences	Yes/No/Unsure		
**6. Comparison of behaviour**<br>6.1. Demonstration of the behavior<br>6.2. Social comparison<br>6.3. Information about others' approval	Yes/No/Unsure		
**7. Associations**	Yes/No/Unsure		

7.1. Prompts/cues<br>7.2. Cue signalling reward<br>7.3. Reduce prompts/cues<br>7.4. Remove access to the reward<br>7.5. Remove aversive stimulus<br>7.6. Satiation<br>7.7. Exposure<br>7.8. Associative learning			
**8. Repetition and substitution**<br>8.1. Behavioral practice/rehearsal<br>8.2. Behavior substitution<br>8.3. Habit formation<br>8.4. Habit reversal<br>8.5. Overcorrection<br>8.6. Generalisation of target behavior<br>8.7. Graded tasks	Yes/No/Unsure		
**9. Comparison of outcomes**<br>9.1. Credible source<br>9.2. Pros and cons<br>9.3. Comparative imagining of<br>future outcomes	Yes/No/Unsure		

**10. Reward and threat**<br>10.1. Material incentive (behavior)<br>10.2. Material reward (behavior)<br>10.3. Non-specific reward<br>10.4. Social reward<br>10.5. Social incentive<br>10.6. Non-specific incentive<br>10.7. Self-incentive<br>10.8. Incentive (outcome)<br>10.9. Self-reward<br>10.10. Reward (outcome)<br>10.11. Future punishment	Yes/No/Unsure		
**11. Regulation**<br>11.1. Pharmacological support<br>11.2. Reduce negative emotions<br>11.3. Conserving mental resources<br>11.4. Paradoxical instructions	Yes/No/Unsure		
**12. Antecedents**<br>12.1. Restructuring the physical environment<br>12.2. Restructuring the social environment	Yes/No/Unsure		

12.3. Avoidance/reducing exposure to cues for the behavior<br><br>12.4. Distraction<br><br>12.5. Adding objects to the environment<br><br>12.6. Body changes			
**13. Identity**<br><br>13.1. Identification of self as role model<br><br>13.2. Framing/reframing<br><br>13.3. Incompatible beliefs<br><br>13.4. Valued self-identify<br><br>13.5. Identity associated with changed behavior	Yes/No/Unsure		
**14. Scheduled consequences**<br><br>14.1. Behavior cost<br>14.2. Punishment<br>14.3. Remove reward<br>14.4. Reward approximation<br>14.5. Rewarding completion<br>14.6. Situation-specific reward<br>14.7. Reward incompatible behavior<br><br>14.8. Reward alternative behavior<br><br>14.9. Reduce reward frequency	Yes/No/Unsure		

14.10. Remove punishment			
**15. Self-belief**<br>15.1. Verbal persuasion about capability<br>15.2. Mental rehearsal of successful performance<br>15.3. Focus on past success<br>15.4. Self-talk	Yes/No/Unsure		
**16. Covert learning**<br>16.1. Imaginary punishment<br>16.2. Imaginary reward<br>16.3. Vicarious consequences	Yes/No/Unsure		

Supplementary file 5. SWIM checklist.

The citation for the Synthesis Without Meta-analysis explanation and elaboration article is: Campbell M, McKenzie JE, Sowden A, Katikireddi SV, Brennan SE, Ellis S, Hartmann-Boyce J, Ryan R, Shepperd S, Thomas J, Welch V, Thomson H. Synthesis without meta-analysis (SWiM) in systematic reviews: reporting guideline BMJ 2020;368:l6890 http://dx.doi.org/10.1136/bmj.l6890

**SWiM is intended to complement and be used as an extension to PRISMA**			
**SWiM reporting item**	**Item description**	**Page in manuscript where item is reported**	**Other***
Methods			
**1** Grouping studies for synthesis	1a) Provide a description of, and rationale for, the groups used in the synthesis (e.g., groupings of populations, interventions, outcomes, study design)	11	
	1b) Detail and provide rationale for any changes made subsequent to the protocol in the groups used in the synthesis	N/A	
**2** Describe the standardised metric and transformation methods used	Describe the standardised metric for each outcome. Explain why the metric(s) was chosen, and describe any methods used to transform the intervention effects, as reported in the study, to the standardised metric, citing any methodological guidance consulted	9-10	
**3** Describe the synthesis methods	Describe and justify the methods used to synthesise the effects for each outcome when it was not possible to undertake a meta-analysis of effect estimates	11	
**4** Criteria used to prioritise results for summary and synthesis	Where applicable, provide the criteria used, with supporting justification, to select the particular studies, or a particular study, for the main synthesis or to draw conclusions from the synthesis (e.g., based on study design, risk of bias assessments, directness in relation to the review question)	S3	

SWiM reporting item	Item description	Page in manuscript where item is reported	Other*
**5** Investigation of heterogeneity in reported effects	State the method(s) used to examine heterogeneity in reported effects when it was not possible to undertake a meta-analysis of effect estimates and its extensions to investigate heterogeneity	N/A	
**6** Certainty of evidence	Describe the methods used to assess certainty of the synthesis findings	P11	
**7** Data presentation methods	Describe the graphical and tabular methods used to present the effects (e.g., tables, forest plots, harvest plots).<br>Specify key study characteristics (e.g., study design, risk of bias) used to order the studies, in the text and any tables or graphs, clearly referencing the studies included	Table 1, Table 2 & Table 3	
Results			
**8** Reporting results	For each comparison and outcome, provide a description of the synthesised findings, and the certainty of the findings. Describe the result in language that is consistent with the question the synthesis addresses, and indicate which studies contribute to the synthesis	Table 1; p18-21	
Discussion			
**9** Limitations of the synthesis	Report the limitations of the synthesis methods used and/or the groupings used in the synthesis, and how these affect the conclusions that can be drawn in relation to the original review question	P25	

PRISMA=Preferred Reporting Items for Systematic Reviews and Meta-Analyses.
*If the information is not provided in the systematic review, give details of where this information is available (e.g., protocol, other published papers (provide citation details), or website (provide the URL)).

Supplementary file 6. Outcome metrics.

Study	Outcome measured	Metric of measurement
Axley et al., 2018	Weight	Lbs
	HDL	mg/dL
	TGs	g/dL
	ALT	IU/L
	AST	IU/L
Björnsdottir et al., 2024	Weight	Kgs
	HbA1c	mmol/L
	HDL	mmol/L
	LDL	mmol/L
	TGs	mmol/L
	Total cholesterol	mmol/L
	ALT	IU/L
	AST	IU/L
	Fat % (MRI-PDFF)	%
	Fibrosis (LSM)	kPa
	Steatosis (CAP)	dB/m
Cho et al., 2024	Weight	Kgs
	HDL	mmol/L
	LDL	mmol/L
	TGs	mmol/L
	Total cholesterol	mmol/L
	ALT	IU/L
	AST	IU/L
	ALP	IU/L
	GGT	IU/L
	Albumin	IU/L
Kaewdech et al., 2024	Weight	Kgs
	ALT	IU/L

	Fibrosis (LSM)<br>Steatosis (CAP)	kPa<br>dB/m
Kwon et al., 2024	Weight<br>ALT<br>AST<br>GGT	Kgs<br>IU/L<br>IU/L<br>IU/L
Lim et al., 2020	Weight<br>ALT<br>AST	Kgs<br>IU/L<br>IU/L
Mazzotti et al., 2018	Weight<br>TGs<br>ALT<br>GGT	%<br>mg/dl<br>IU/L<br>IU/L
Motz et al., 2021	Weight<br>HbA1c<br>ALT<br>AST<br>Fat % (MRI-PDFF)	%<br>%<br>IU/L<br>IU/L<br>%
Sato et al., 2023	Weight<br>HbA1c<br>LDL<br>TGs<br>ALT<br>AST<br>ALP<br>GGT	Kgs<br>%<br>mg/dl<br>mg/dl<br>IU/L<br>IU/L<br>IU/L<br>IU/L
Stine et al., 2023	Weight<br>ALT<br>AST<br>ALP	Kgs<br>IU/L<br>IU/L<br>IU/L
Tincopa et al., 2022	Weight<br>HbA1c	Lbs<br>mmol/L

	HDL	mg/dl
	LDL	mg/dl
	TGs	mg/dl
	ALT	IU/L
	Fibrosis (LSM)	kPa
	Steatosis (CAP)	dB/m

Supplementary file 7. Risk of bias assessments.

Summary of risk of bias (RoB 2 tool)

Summary of risk of bias (ROBINS-I tool)

Summary of risk of bias (NHLBI pre-post tool)

**Study**	**Quality score**	**Quality rating**
Björnsdottir et al., 2024	8/11 (72.7%)	Fair
Motz et al., 2021	5/11 (45.5%)	Poor
Sato et al., 2023	9/11 (81.8%)	Fair
Tincopa et al., 2022	7/11 (63.3%)	Fair
