# Supplementary material for: Effectiveness and active ingredients of digital behaviour change interventions for MASLD: A systematic review and meta-analysis
Source: JHEP Rep. 2025 Jul 2;7(10):101507. doi: 10.1016/j.jhepr.2025.101507 (PMC12448008; doi:10.1016/j.jhepr.2025.101507)
Supplement: Multimedia component 4 [file mmc4.pdf]

JHEP|Reports

Effectiveness and active ingredients of digital behaviour change interventions for MASLD: A systematic review and meta-analysis

Authors

Hollie Smith, Rebecca Livingston, Kirsten Ashley, …, Alison Innerd, Kate Hallsworth, Leah Avery

Correspondence

leah.avery@tees.ac.uk (L. Avery).

Graphical abstract

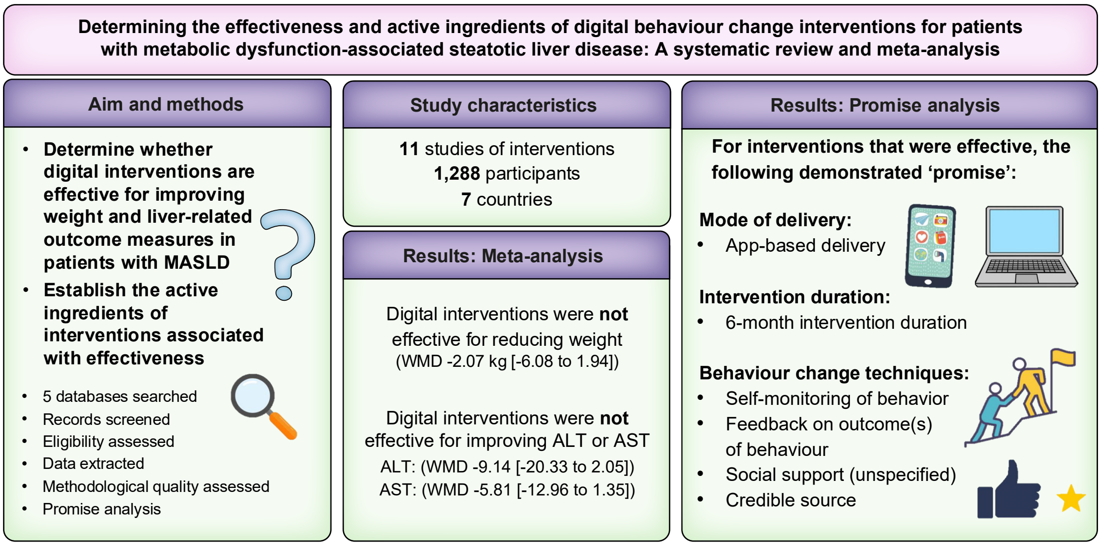

Highlights:

- Eleven digital behaviour change interventions to support patients with MASLD were identified by the review.
- Successful management of MASLD relies on targeting change in lifestyle behaviours, but behaviours were rarely measured.
- Digital interventions were not effective for improving weight, ALT, or AST.
- App-based delivery, ≥6-month duration, and self-regulation content were associated with intervention effectiveness.
- Inclusion of specific intervention ingredients increases the likelihood of effectiveness.

Impact and implications:

This review is the first of its kind to report on the effectiveness and active ingredients of digital behaviour change interventions for the management of MASLD. Although the interventions reviewed were not effective overall, specific features and content of those interventions were associated with effectiveness. These insights can be used to inform the development of new interventions or to optimise existing interventions that could improve effectiveness. Findings also suggest that digital behaviour change interventions are beneficial for a proportion of individuals, and future research should focus on identifying who those individuals are. Significant heterogeneity between interventions was evident in terms of mode of delivery, behavioural change content, duration, and frequency of delivery. To truly determine the effectiveness of digital behaviour change interventions for patients with MASLD, they should be systematically developed using behaviour change theory and in accordance with a recognised intervention development framework.

https://doi.org/10.1016/j.jhepr.2025.101507

© 2025 The Authors. Published by Elsevier B.V. on behalf of European Association for the Study of the Liver (EASL). This is an open access article under the CC BY license (http://creativecommons.org/licenses/by/4.0/).

JHEP|Reports

Effectiveness and active ingredients of digital behaviour change interventions for MASLD: A systematic review and meta-analysis

Hollie Smith[1], Rebecca Livingston[2], Kirsten Ashley[1], Matthew Cooper[3], Stuart McPherson[4,5,6], Alison Innerd[1], Kate Hallsworth[4,5,6], Leah Avery[1,6,*]

JHEP Reports **2025**. vol. 7 | 1–17

Check for updates

Background & Aims: Metabolic dysfunction-associated steatotic liver disease (MASLD) is the most prevalent liver condition worldwide. Successful management relies on targeting changes in lifestyle behaviours. Digital behaviour change interventions present a scalable approach to lifestyle change. The aim of this systematic review was to determine the effectiveness and active ingredients of digital behavior change interventions for improving weight and liver-related outcome measures in patients with MASLD.

Methods: Five databases were searched up to 31 January 2025 for studies reporting on digital lifestyle behaviour change interventions for patients with MASLD. Data were meta-analysed or narratively synthesised depending on study design. Intervention content and features positively associated with changes in outcomes of interest were identified using promise analysis.

Results: Eleven studies involving 1,288 participants fulfilled the review criteria. Digital behavior change interventions were not effective for reducing weight (weighted mean difference [WMD] -2.07 kg [-6.08 to 1.94 kg]). Likewise, they did not lead to statistically significant improvements in alanine transaminase and aspartate transaminase (WMD -9.14 [-20.33 to 2.05] and WMD -5.81 [-12.96 to 1.35], respectively). Interventions varied in terms of mode of delivery (*e.g.* app and SMS), duration (1–11 months), and frequency of delivery (three times/week to continuous access). Promising intervention features/content included app-based delivery, ≥6-month duration, and self-monitoring of behaviour, feedback on outcomes, and social support.

Conclusions: Digital behaviour change interventions did not improve weight and liver-related outcomes measures in patients with MASLD. However, the inclusion of proposed specific intervention ingredients is likely to improve effectiveness.

© 2025 The Authors. Published by Elsevier B.V. on behalf of European Association for the Study of the Liver (EASL). This is an open access article under the CC BY license (http://creativecommons.org/licenses/by/4.0/).

Introduction

Metabolic dysfunction-associated steatotic liver disease (MASLD) is defined as the presence of hepatic steatosis on imaging or histology in conjunction with at least one cardiometabolic risk factor and no other discernible cause.[1] MASLD is the most common liver condition, affecting upwards of 30% of adults globally,[2] and is related to lifestyle factors such as regular consumption of energy-dense, ultra-processed foods and low levels of physical activity that contribute towards overweight and obesity.[3]

Despite the prevalence of MASLD, management of the condition is variable.[4] Clinical practice guidelines recommend lifestyle modification (*i.e.* changes to diet and physical activity behaviours) to facilitate weight loss for all patients with MASLD.[3] A reduction in body weight of ≥5% is associated with a reduction in liver fat, a reduction of 7–10% is associated with an improvement in liver inflammation, and a reduction of ≥10% is associated with an improvement in fibrosis.[5]

However, despite the dose–response relationship between the amount of weight lost and the extent of improvement in liver disease biomarkers,[6] changing lifestyle behaviours remains a challenge, and many patients do not achieve guideline-recommended weight loss goals.[7,8] Barriers to changing lifestyle behaviours can be attributed to many factors, including patients not receiving appropriate support, stigma, comorbidities, constraints on time preventing attendance at appointments (*e.g.* long working hours and caring responsibilities), and access to healthcare and other facilities (*e.g.* distance to clinic and appointment availability) that are not conducive to facilitating, enacting, and sustaining lifestyle behaviour change.[9–11] In the context of MASLD, healthcare professionals (HCPs) have raised concerns about supporting patients to make changes to their lifestyle behaviours because of a lack of specific training, appropriate interventions, and referral pathways.[12–14] Furthermore, the prevalence of MASLD globally highlights the necessity to scale

* Corresponding author. Address: School of Health and Life Sciences, Teesside University TS1 3BA, Middlesbrough, UK.
E-mail address: leah.avery@tees.ac.uk (L. Avery).
https://doi.org/10.1016/j.jhepr.2025.101507

up clinical services/interventions to meet growing demands in a complex patient population.

Digital technology allows health services and interventions to be delivered or enriched via the internet and mobile applications at scale.[15] Digital healthcare delivery is an innovative and cost-effective method to meet the demand for long-term support for those living with chronic conditions.[16] Several meta-analyses have highlighted the positive effect of digital interventions on measures of health,[17,18] and digital lifestyle interventions are currently being used to support patients at risk of other long-term conditions. An example of such an intervention is the Diabetes Prevention Programme (DPP). The DPP was created by the UK National Health Service (NHS) and Diabetes UK and provides a 9-month, evidence-based digital lifestyle behaviour change programme[19] for individuals at risk of type 2 diabetes. It includes personalised support using digital tools, such as apps and wearable technologies, and provides health coaches and online peer support groups that aim to support patients to change and maintain changes in lifestyle behaviours. The DPP has proven to be effective, evidenced by clinically significant reductions in weight and HbA_{1c}, and acceptable to patients.[20]

A recent systematic review reported on the effectiveness of digital behaviour change interventions for facilitating weight loss in patients with MASLD.[21] Although the findings concluded that the interventions were effective overall, the analysis included only the intervention arms of eight studies with small sample sizes in the meta-analysis. They were single-group studies (n = 4), randomised controlled trials (RCTs) (n = 3), and non-RCTs (n = 1). Furthermore, the specific features and active ingredients of the digital behaviour change interventions (*i.e.* those that contribute to intervention effectiveness) were not identified or reported. As such, the aim of this systematic review was to determine the effectiveness of digital behaviour change interventions for improving weight and liver-related outcomes in patients diagnosed with MASLD and to identify the active ingredients of these interventions, namely, the specific features and content that lead to changes in the outcomes of interest. The specific research questions were as follows:

1. Are digital behaviour change interventions effective for improving weight in patients with MASLD?
2. Are digital behaviour change interventions effective for improving liver-related outcome measures in patients with MASLD?
3. What are the active ingredients of digital behaviour change interventions for patients with MASLD that lead to improvements in weight, metabolic outcomes, and/or liver-related outcome measures?

Materials and methods

Search strategy and study selection

This systematic review was conducted with reference to a registered protocol (CRD42023406827),[22] and adhered to the Preferred Reporting Items for Systematic Reviews and Meta-Analysis (PRISMA) guidelines[23] (Supplementary file 1).

Five electronic databases (MEDLINE, CINAHL, Web of Science, PsycINFO, and SCOPUS) were searched from inception to 31 January 2025 using a combination of MeSH headings and keywords (Supplementary file 2). No limits or restrictions were applied. Before conducting the search, scoping searches were performed to refine the search strategy. Manual searches of end reference lists and citation searches of included studies were conducted to identify potentially relevant studies not captured by the electronic search.

Search results underwent a process of electronic deduplication that was checked for accuracy by one reviewer (HS). The same reviewer screened the titles and abstracts of all references retrieved by the search, and a second reviewer (RL) independently screened a 20% random sample (Stage 1 screening). Articles retained following screening of titles and abstracts were retrieved in full text and independently screened by HS and one of four reviewers (RL, LA, SMc, or KH) against eligibility criteria using a study selection form (Stage 2 screening) (Supplementary file 3). Disagreements between reviewers were resolved by discussion.

Eligibility criteria

Studies reporting on a digital (*e.g.* web, app, SMS, instant messaging, e-mail, and video conferencing) behaviour change interventions designed to facilitate improvements in weight and liver-related outcome measures in patients aged ≥18 years with MASLD or metabolic dysfunction-associated steatohepatitis (MASH) were included in the review. Studies reporting on participants with non-alcoholic fatty liver disease were also eligible for inclusion. It was a requirement for studies to report the impact of the digital intervention on weight, metabolic outcomes (*e.g.* HbA_{1c}, high-density lipoprotein [HDL], low-density lipoprotein [LDL], triglycerides [TGs], and total cholesterol), and liver-related measures (*e.g.* hepatic fibrosis using liver stiffness measurement [LSM], hepatic steatosis using controlled attenuation parameter [CAP], liver fat % using magnetic resonance imaging proton density fat fraction [MRI-PDFF], and liver enzymes, including alanine transaminase [ALT], aspartate aminotransferase [AST], alkaline phosphatase [ALP], gamma-glutamyl transferase [GGT], and albumin). In instances where studies reported on the impact of the digital intervention on lifestyle behaviours (*e.g.* physical activity, diet, and alcohol), data were extracted and reported. No limits were applied to year or country of publication.

Data extraction

A standardised data extraction form (Supplementary file 4) was developed to capture the following information: study characteristics (country of origin, aims, design, eligibility criteria, sampling method, sample size, follow-up period, and loss to follow-up), patient characteristics (age, sex/gender, ethnicity, BMI, and comorbidities), intervention characteristics (name, mode of digital delivery, intervention frequency, duration, content, and use of theory in intervention design), outcomes assessed, including findings, and theory-linked behaviour change techniques (BCTs), that is, theory-linked strategies for changing one or more mechanism of action impacting determinants of behaviour (*e.g.* attitudes and self-efficacy), and subsequently behaviour itself.[24] The data extraction form was piloted by two reviewers (HS and KA) using one included study and subsequently refined.

Data were extracted from studies by one reviewer (HS) and checked by a second reviewer (RL, LA, SMc, KH, or AI). One reviewer (HS) independently coded the presence of BCTs within interventions using the Behaviour Change Technique Taxonomy version 1 (BCTTv1).[24] BCTTv1 is an extensive

taxonomy of 93 consensually agreed, distinct BCTs that offers a method for specifying interventions. A second reviewer (KA) checked 50% of the extracted information for accuracy. Both reviewers had previously completed the BCTTv1 online training, and one reviewer was an experienced coder (KA). Only BCTs included in the interventions that were over and above the standard care/control/comparator group were coded; that is, BCTs present in both the intervention and standard care/control/comparator were not coded.

Methodological quality assessment

The methodological quality of all included studies was independently assessed by HS and one other reviewer (KA, MC, or LA) using the revised experimental Risk of Bias tool for Randomised Trials (ROB-2),[25] the Risk of Bias In Non-randomised Studies of Interventions (ROBINS-I) tool,[26] or the National Heart, Lung, and Blood Institute (NHLBI) Quality Assessment Tool for Before–After (Pre–Post) Studies with No Control Group.[27] A third reviewer was consulted to resolve any discrepancies.

Data synthesis

The *Cochrane Handbook for Systematic Reviews of Interventions*[28] suggests that the results from different study designs should be expected to differ systematically, resulting in increased heterogeneity. As such, the handbook recommends that RCTs and non-randomised experimental studies (*e.g.* single-group pre–post studies) should not be combined with RCTs in a meta-analysis. Therefore, data on changes in weight, ALT, and AST from RCTs only were synthesised using a random-effects meta-analysis in Review Manager (RevMan 8.14.0, available at revman.cochrane.org). Outcomes of single-group pre–post studies and non-randomised studies were synthesised narratively using the Synthesis Without Meta-Analysis Guidelines (SWiM; Supplementary files 5 and 6).

Promise of intervention content and features

To facilitate the design of effective interventions, it is important to understand the specific content and features that are associated with effectiveness, that is, their active ingredients[29] (*e.g.* mode of delivery, duration, intensity and frequency of delivery, and specific BCTs). To achieve this, we calculated promise ratios.[30,31] Promise analysis is an accepted method for identifying the features/content of interventions associated with positive outcomes/effectiveness.

Firstly, interventions were classified as very promising (statistically significant between-group improvements in weight/metabolic outcomes and/or liver-related outcome measures in favour of the intervention group); quite promising (intervention groups with statistically significant within-group improvements in weight/metabolic outcomes and/or liver-related outcome measures, or improvements greater than those seen in standard care/control/comparator); or non-promising (no statistically significant within- or between-group improvements in weight/metabolic outcomes and/or liver-related measures).

To calculate a ‘promise ratio’, very or quite promising interventions that contained a specific active ingredient were summed and subsequently divided by the number of non-promising interventions that contained the same active ingredient. Active ingredients found in at least twice as many very or quite promising interventions compared with non-promising interventions were classified as promising (promise ratio of ≥2).[30]

Results

The study selection process is presented in Fig. 1. Eleven articles reporting on 11 digital behaviour change interventions involving 1,288 participants met the eligibility criteria and were retained for review.[32–42] Six studies were RCTs,[32,34–37,41] four were single-group pre–post studies,[33,39,40,42] and one was a non-RCT[38].

Study characteristics

A summary of all included studies is reported in Table 1. Studies were published between 2018 and 2024. They were conducted in the USA,[32,39,41,42] South Korea,[34,36] Italy,[38] Iceland,[33] Japan,[40] Singapore,[37] and Thailand.[35] Five interventions were delivered via app,[33,34,37,40,41] one of which was delivered alongside the provision of a daily partial meal replacement product.[34] Two interventions were delivered via SMS/instant messaging,[32,35] two interventions were delivered via a combination of modes of delivery (*i.e.* app and SMS[36] and app and e-mail[42]), one intervention was delivered via a website,[38] and one intervention was delivered via video software.[39] The duration of intervention delivery varied from 1 month,[34] 3 months,[33] 4 months,[41] 5 months,[3] 6 months,[32,35–38,42] to 11 months[40].

Change in weight (kg, lbs, or %)

Digital interventions (compared with standard care/control/comparator) showed no statistically significant improvements in weight (weighted mean difference [WMD] -2.07 kg, 95% CI -6.08 to +1.94 kg, I^2 = 90%) based on data from six RCTs[32,34–37,41] (Fig. 2).

Of the remaining five intervention studies (four single-group pre–post studies[33,39,40,42] and one non-RCT[38]), two studies reported statistically significant post-intervention weight loss of -7.3 kg (p <0.001)[40] and -3.5 kg (p <0.001).[33] One study[38] reported statistically significant reductions in body weight of -3.4% at 6-month follow-up, -4.9% at 12-month follow-up, and -5.5% at 24-month follow-up (all p <0.001). The remaining two studies reported changes in body weight of -5.1%[39] and +7 lbs,[42] but neither at a statistically significant level.

Change in liver-related measures

ALT

Digital behavior change interventions, when compared with standard care/control/comparator, showed no statistically significant improvements in ALT (WMD -9.14 IU/L, 95% CI -20.33 to +2.05 IU/L, I^2 = 81%) based on data from six RCTs[32,34–37,41] (Fig. 3).

The five intervention studies not included in the meta-analysis (four single-group pre–post studies[33,39,40,42] and one non-RCT[38]) also assessed ALT as an outcome. Changes in ALT ranging from -49.5 to +1.8 IU/L were observed across studies (mean -20.9 IU/L, SD 15.62 IU/L). However, statistically

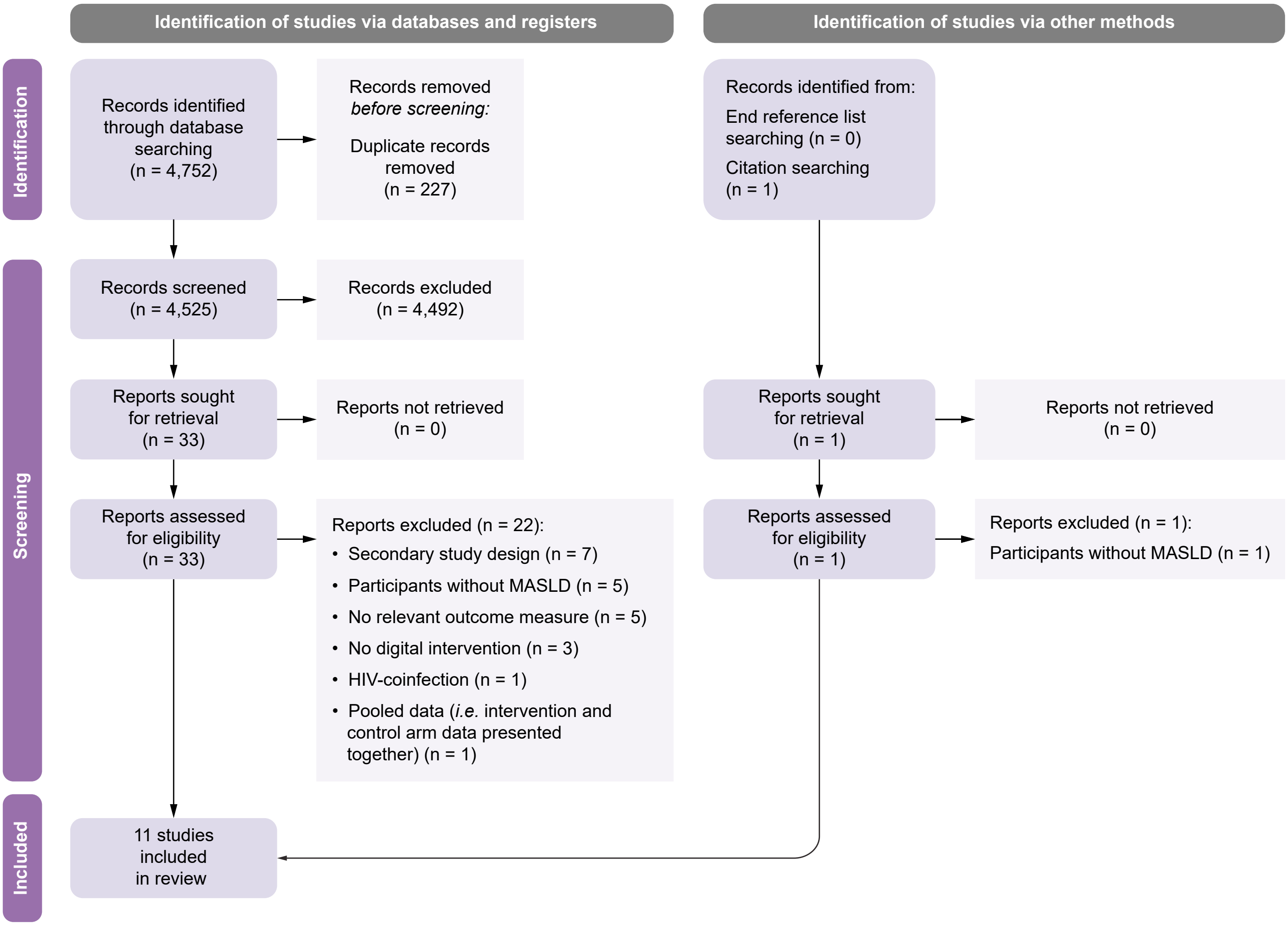

Fig. 1. PRISMA flow diagram. MASLD, metabolic dysfunction-associated steatotic liver disease; PRISMA, Preferred Reporting Items for Systematic reviews and Meta-Analyses.

Study or Subgroup	Digital intervention Mean [kgs]	Digital intervention SD [kgs]	Digital intervention Total	Standard care/control/comparator Mean [kgs]	Standard care/control/comparator SD [kgs]	Standard care/control/comparator Total	Weight	Mean difference IV, Random, 95% CI [kgs]
Axley (2018)	103	6.4	13	105	6.4	17	16.9%	-2.00 [-6.62, 2.62]
Cho (2024)	88	2.6	25	86	1.5	24	22.8%	2.00 [0.82, 3.18]
Kaewdech (2024)	69	7.3	61	70	10.8	61	19.6%	-1.00 [-4.27, 2.27]
Kwon (2024)	81	19.6	48	82	16.1	54	12.4%	-1.00 [-8.01, 6.01]
Lim (2020)	78	4.2	55	86	2.9	53	22.6%	-8.00 [-9.36, -6.64]
Stine (2023)	97	19.9	20	98	22.2	20	5.7%	-1.00 [-14.07 , 12.07]
**Total (HKSJ[a])**			**222**			**229**	**100.0%**	**-2.07 [-6.08 , 1.94]**
95% prediction interval								[-12.61, 8.47]

Test for overall effect: T = 1.33, df = 5 (p = 0.24)
Test for subgroup differences: Not applicable
Heterogeneity: Tau² (REML[b], 95% CI) = 14.38 [3.67 , 64.97]; Chi² = 119.28, df = 5 (p <0.00001); I^2 = 90%

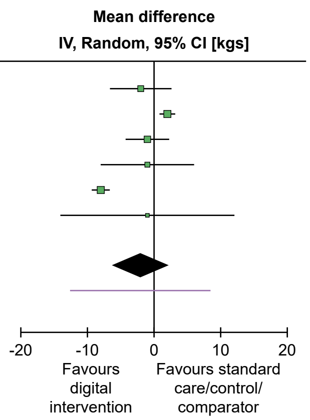

Fig. 2. Random-effects meta-analysis for weight (kg).

significant changes were reported in only one study[40] (reduction of -49.5 IU/L at post-intervention follow-up; p <0.001).

AST

Digital interventions (compared with standard care/control/ comparator) showed no statistically significant improvements in AST (WMD -5.81 IU/L, 95% CI -12.96 to +1.35 IU/L, I^2 = 55%) based on data from five RCTs.[32,34,36,37,41] (Fig. 4).

Three single-group pre–post studies[33,39,40] assessed AST as an outcome. Changes ranged from -23.1 to +1.5 IU/L (mean -12.2 IU/L, SD 9.53 IU/L), although these changes were only statistically significant for one study[40] (reduction of -18.8 IU/L at post-intervention follow-up; p = 0.003).

Study or Subgroup	Digital intervention Mean [IU/L]	SD [IU/L]	Total	Standard care/control/comparator Mean [IU/L]	SD [IU/L]	Total	Weight	Mean difference IV, Random, 95% CI [IU/L]
Axley (2018)	32	3	13	51	11	17	19.8%	-19.00 [-24.48, -13.52]
Cho (2024)	49	25.9	25	54	15.5	24	15.2%	-5.00 [-16.90, 6.90]
Kaewdech (2024)	31	25.2	61	26	17.8	61	18.3%	5.00 [-2.74, 12.74]
Kwon (2024)	31	23.3	48	37	32.5	54	16.0%	-6.00 [-16.89, 4.89]
Lim (2020)	42	40.4	55	67	35.2	53	13.5%	-25.00 [-39.28, -10.72]
Stine (2023)	39	17.4	20	46	11.8	20	17.2%	-7.00 [-16.21, 2.21]
**Total (HKSJ[a])**			**222**			**229**	**100.0%**	**-9.14 [-20.33, 2.05]**
95% prediction interval								[-35.80, 17.53]

Test for overall effect: T = 2.10, df = 5 (p = 0.09)
Test for subgroup differences: Not applicable
Heterogeneity: Tau² (REML[b], 95% CI) = 88.67 [20.98, 666.61]; Chi² = 30.62, df = 5 (p <0.0001); I^2 = 81%

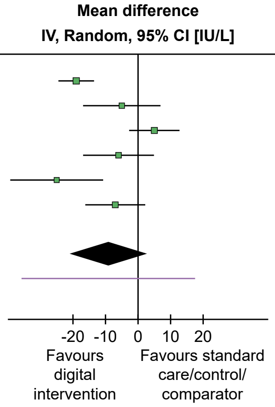

Fig. 3. Random-effects meta-analysis for ALT (IU/L). ALT, alanine transaminase.

Study or Subgroup	Digital intervention Mean [IU/L]	SD [IU/L]	Total	Standard care/control/comparator Mean [IU/L]	SD [IU/L]	Total	Weight	Mean difference IV, Random, 95% CI [IU/L]
Axley (2018)	37	4	13	47	8	17	29.7%	-10.00 [-14.38, -5.62]
Cho (2024)	34	20.3	25	36	13	24	16.4%	-2.00 [-11.51, 7.51]
Kwon (2024)	30	12.5	48	36	30.8	54	17.6%	-6.00 [-14.94, 2.94]
Lim (2020)	32	27.5	55	43	17.6	53	18.2%	-11.00 [-19.68, -2.32]
Stine (2023)	37	16.3	20	34	11.3	20	18.1%	3.00 [-5.69, 11.69]
**Total (HKSJ[a])**			**161**			**168**	**100.0%**	**-5.81 [-12.96, 1.35]**
95% prediction interval								[-19.59, 7.97]

Test for overall effect: T = 2.25, df = 4 (p = 0.09)
Test for subgroup differences: Not applicable
Heterogeneity: Tau² (REML[b], 95% CI) = 18.00 [0.00, >179.97366732032785]; Chi² = 8.81, df = 4 (p = 0.07); I^2 = 55%

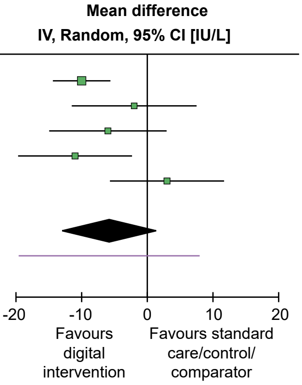

Fig. 4. Random-effects meta-analysis for AST (IU/L). AST, aspartate aminotransferase.

Other liver function tests (GGT, ALP, and albumin)

Four studies (two RCTs,[34,36] one single-group pre–post study,[40] and one non-RCT[38]) assessed GGT as an outcome. Three studies reported a statistically significant improvement in GGT within intervention groups, with a mean improvement of -21.28 IU/L (SD 6.61 IU/L). One RCT[34] reported a statistically significant reduction of -27.76 IU/L (p = 0.007) from baseline to post-intervention follow-up in the intervention group and a statistically significant difference between groups (p = 0.014). The second RCT[36] reported a statistically significant reduction of -12.2 IU/L within the intervention group (p = 0.04), but this did not significantly differ from the control group. The single-group pre–post study[40] reported a statistically significant reduction of -28.6 IU/L (p = 0.02) from baseline to post-intervention follow-up. The non-RCT[38] narratively reported significant improvements in GGT from baseline to post-intervention follow-up and between groups.

Three studies (two RCTs[34,41] and one single-group pre–post study[40]) assessed ALP as an outcome. The single-group pre–post study[40] reported statistically significant reductions of -10.6 IU/L (p <0.001) from baseline to post-intervention follow-up. Of the two RCTs,[34,41] neither reported statistically significant within- or between-group differences.

One RCT[34] assessed albumin as an outcome. No statistically significant within-group improvements were observed in the intervention group from baseline to post-intervention follow-up, although there was a statistically significant difference between groups (p = 0.017).

Fibrosis (LSM; kPa), steatosis (CAP; dB/m), and liver fat percentage (MRI-PDFF)

Three studies (two single-group pre–post studies[33,42] and one RCT[35]) assessed fibrosis (LSM; kPa) and steatosis (CAP; dB/m) as outcomes. The RCT[35] reported a statistically significant reduction in LSM (-0.4 kPa) from baseline to post-intervention follow-up in the intervention group (p = 0.003), and when compared with the standard care group (p = 0.035). Both single-group pre–post studies[33,42] reported no statistically significant changes in LSM from baseline to post-intervention follow-up. The RCT also reported a statistically significant reduction in CAP from baseline to post-intervention follow-up of -20.2 dB/m (p <0.001) in the intervention group, but no between-group differences were reported (p = 0.655).[35] One single-group pre–post study[33] reported a statistically significant reduction in CAP of -33.3 dB/m (p <0.001) from baseline to post-intervention follow-up. The second pre–post study[42] reported no statistically significant changes.

Two single-group pre–post studies assessed fat percentage (MRI-PDFF) as an outcome.[33,39] The first study reported a statistically significant reduction of -2.2% in fat percentage (p

Table 1. Summary of study characteristics.

Study ID, country of origin, study design	Sample details and demographics	Intervention group	Intervention mode of delivery, duration, and frequency of access	Standard care/ control/comparator group	BCTs coded in intervention group over and above standard care/control/comparator group	Change in weight and metabolic outcomes from baseline to all follow-up points*	Change in liver-related outcome measures from baseline to all follow-up points*
**Axley *et al.* (2018)**[32]<br>USA<br>Pilot RCT	Total sample: 30<br>• Intervention: 13 enrolled; 13 analysed<br>• Control: 17 enrolled; 17 analysed<br>Age (years), mean (SD):<br>• Intervention: 54 (2.7)<br>• Control: 52 (2.3)<br>Female, n (%):<br>• Intervention: 11 (85)<br>• Control: 8 (47)<br>BMI (kg/m^2), mean (SD):<br>• Intervention: 39 (2.4)<br>• Control: 36 (1.9)	Intervention name: No name reported<br>Intervention content: education on nutrition, exercise, stress, and weight management; prompts to set goals with actionable tips provided; information about overcoming barriers with actionable tips<br>Theoretical underpinning: not reported	Mode of delivery: SMS/instant messaging<br>Duration: 6 months<br>Frequency: 3 uni- and bi-directional SMS/ week for 6 months	Standard care/control/ comparator content: standard of care for liver disease with detailed instructions on healthy diet and daily exercise for weight loss	**1.2** Problem solving<br>**1.3** Goal setting (outcome)<br>**3.1** Social support (unspecified)<br>**7.1** Prompts/cues	6-month follow-up:<br>Mean weight (lbs)<br>• **Intervention: -6**<br>• Control: +2<br>• **Between groups**<br>Mean HDL (mg/dl)<br>• Intervention: -2<br>• Control: 0<br>• Between groups<br>Mean TGs (g/dl)<br>• **Intervention: -17**<br>• Control: -41<br>• Between groups	6-month follow-up:<br>Mean ALT (IU/L)<br>• **Intervention: -12**<br>• Control: -6<br>• Between groups<br>Mean AST (IU/L)<br>• **Intervention: -9**<br>• Control: 0<br>• Between groups
**Björnsdottir *et al.* (2024)**[33]<br>Iceland<br>Single-group pre–post study	Total sample: 38<br>• Intervention: 38 enrolled; 38 analysed<br>Age (years), median (IQR):<br>• Intervention: 59.5 (46.3–68.8)<br>Female, n (%):<br>• Intervention: 23 (61)<br>BMI (kg/m^2), mean (SD):<br>• Intervention: 37.6 (5.8)	Intervention name: SK-241<br>Intervention content: education videos; self-regulation tools (food journal, step counter, and quality of life rating scale); mindfulness and meditation; health coaching<br>Theoretical underpinning: not reported	Mode of delivery: app<br>Duration: 3 months<br>Frequency: continuous access	Standard care/control/ comparator content: N/A	**2.2** Feedback on behaviour<br>**2.3** Self-monitoring of behaviour<br>**3.1** Social support (unspecified)	3-month follow-up:<br>Mean weight (kg)<br>• **Intervention: -3.5**<br>Median HbA_{1c} (mmol/ L)<br>• **Intervention: 0.5**<br>Mean HDL (mmol/L)<br>• Intervention: +0.01<br>Mean LDL (mmol/L)<br>• Intervention: 0<br>Median TGs (mmol/L)<br>• **Intervention: -0.20**<br>Mean total cholesterol (mmol/L)<br>• Intervention: -0.1	3-month follow-up:<br>Median ALT (IU/L)<br>• Intervention: +1.8<br>Median AST (IU/L)<br>• Intervention: +1.5<br>Mean fat %: MRI-PDFF (%)<br>• **Intervention: -2.2**<br>Median fibrosis: LSM (kPa)<br>• Intervention: +0.2<br>Mean steatosis: CAP (dB/m)<br>**-33.3**

(*continued on next page*)

Table 1. (*continued*)

Study ID, country of origin, study design	Sample details and demographics	Intervention group	Intervention mode of delivery, duration, and frequency of access	Standard care/control/comparator group	BCTs coded in intervention group over and above standard care/control/comparator group	Change in weight and metabolic outcomes from baseline to all follow-up points*	Change in liver-related outcome measures from baseline to all follow-up points*
**Cho *et al.* (2024)**[34]<br>South Korea<br>Pilot RCT	Total sample: 60<br>• Intervention: 30 enrolled; 25 analysed<br>• Control: 30 enrolled; 24 analysed<br>Age (years), mean (SD):<br>• Intervention: 41.3 (6.7)<br>• Control: 43.5 (6.7)<br>Female (n, %):<br>• Intervention: 0 (0)<br>• Control: 3 (12.5)<br>BMI (kg/m^2), mean (SD):<br>• Intervention: 30 (4.5)<br>• Control: 30.2 (3.7)	Intervention name: Dr. Coach<br>Intervention content: personalised nutrition prescription, self-monitoring of weight diet, activity levels, emotions, and sleep; health coaching to offer knowledge education, goal setting and planning support, monitoring and feedback, and helping actions (i.e. reminders, alternatives for obstacles); 1/day partial meal replacement<br>Theoretical underpinning: not reported	Mode of delivery: app<br>Duration: 1 month<br>Frequency: continuous access to app; 1 evening meal partial meal replacement/day	Standard care/control/comparator content: Standard care consisting of brief education provided by healthcare providers	**1.1** Goal setting (behaviour)<br>**1.4** Action planning<br>**2.2** Feedback on behaviour<br>**2.3** Self-monitoring of behaviour<br>**2.4** Self-monitoring of outcome(s) of behaviour<br>**2.7** Feedback on outcome(s) of behaviour<br>**3.1** Social support (unspecified)<br>**7.1** Prompts/cues<br>**8.3** Habit formation<br>**8.4** Habit reversal<br>**12.5** Adding objects to the environment	1-month follow-up:<br>Mean weight (kg)<br>• Intervention: -2.93<br>• Control: -2.74<br>• Between groups<br>Mean HDL (mmol/L)<br>• Intervention: -1.2<br>• Control: -1.04<br>• Between groups<br>Mean LDL (mmol/L)<br>• Intervention: -2.95<br>• Control: -1.39<br>• Between groups<br>Mean TGs (mmol/L)<br>• Intervention: -43.84<br>• Control: -25.96<br>• Between groups<br>Mean total cholesterol (mmol/L)<br>• Intervention: -12.92<br>• Control: -5.54<br>• Between groups	1-month follow-up:<br>Mean ALT (IU/L)<br>• **Intervention: -28.32**<br>• Control: -10.67<br>• **Between groups**<br>Mean AST (IU/L)<br>• **Intervention: -13.28**<br>• Control: -4.25<br>• Between groups<br>Mean ALP (IU/L)<br>• Intervention: -0.96<br>• Control: +1.46<br>• Between groups<br>Mean GGT (IU/L)<br>• **Intervention: -27.76**<br>• Control: +2.79<br>• **Between groups**<br>Mean albumin (IU/L)<br>• Intervention: -0.06<br>• Control: +0.07<br>• **Between groups**
**Kaewdech *et al.* (2024)**[35]<br>Thailand<br>RCT	Total sample: 122<br>• Intervention: 61 enrolled; 61 analysed<br>• Control: 61 enrolled; 61 analysed<br>Age (years), median (IQR):<br>• Intervention: 53.8 (46.6–57.4)<br>• Control: 52.9 (43.2– 59.7)<br>Female, n (%):<br>• Intervention: 38 (62.3)<br>• Control: 41 (67.2)<br>BMI (kg/m^2), median (IQR):<br>• Intervention: 28 (24.8–30.1)<br>• Control: 27.1 (24.9–29.3)	Intervention name: LINE<br>Intervention content: standard briefing video clip; knowledge broadcast about diet, physical activity, and exercise via video, infographics, and text; reminders to carry out lifestyle changes that were broadcast via messages<br>Theoretical underpinning: Not reported	Mode of delivery: SMS/instant messaging<br>Duration: 6 months<br>Frequency: 3–7 instant messages/week	Standard care/control/comparator content: access to standard briefing video clip (broadcast to all patients at enrolment)	**7.1** Prompts/cues	6-month follow-up:<br>Median weight (kg)<br>• **Intervention: -1**<br>• **Control: -2.2**<br>• Between groups	6-month follow-up:<br>Median ALT (IU/L)<br>• **Intervention: -5**<br>• **Control: -14**<br>• Between groups<br>Median fibrosis: LSM (kPa)<br>• **Intervention: -0.4**<br>• Control: -0.2<br>• **Between groups**<br>Mean steatosis: CAP (dB/m)<br>• **Intervention: -20.2**<br>• **Control: -25**<br>• Between group

(*continued on next page*)

Table 1. (*continued*)

Study ID, country of origin, study design	Sample details and demographics	Intervention group	Intervention mode of delivery, duration, and frequency of access	Standard care/control/comparator group	BCTs coded in intervention group over and above standard care/control/comparator group	Change in weight and metabolic outcomes from baseline to all follow-up points*	Change in liver-related outcome measures from baseline to all follow-up points*
**Kwon *et al.* (2024)**[36]<br>South Korea<br>RCT	Total sample: 111<br>• Intervention: 51 enrolled; 48 analysed<br>• Control: 60 enrolled; 54 analysed<br>Age (years), mean (SD):<br>• Intervention: 51 (13.4)<br>• Control: 47.1 (13.9)<br>Female, n (%):<br>• Intervention: 17 (29)<br>• Control: 31 (46)<br>BMI (kg/m^2), mean (SD):<br>• Intervention: 29.9 (4.1)<br>• Control: 29.6 (4.6)	Intervention name: SMART-Liver<br>Intervention content: self-monitoring of nutritional intake, exercise, sleep, alcohol intake, and smoking; educational slides for MASLD treatment, nutrition, and exercise; health coaching to set goals, identify barriers/facilitators to behaviour change, and provide feedback; Daily SMS to remind participants about daily 'missions'; real-time chat channel<br>Theoretical underpinning: not reported	Mode of delivery: app and SMS<br>Duration: 6 months<br>Frequency: continuous access to app; 1 SMS/day	Standard care/control/comparator content: phone call after enrolment to obtain weight and inform on the importance of weight loss, diet, and exercise for MASLD management; standard clinical care conducted for 6 months, with contact at 3 and 6 months to obtain weight data	**1.1** Goal setting (behaviour)<br>**1.3** Goal setting (outcomes)<br>**1.4** Action planning<br>**1.5** Review behaviour goal(s)<br>**1.7** Review outcome goal(s)<br>**2.2** Feedback on behaviour<br>**2.3** Self-monitoring of behaviour<br>**3.1** Social support (unspecified)<br>**4.1** Instruction on how to perform the behaviour<br>**6.1** Demonstration of the behaviour<br>**7.1** Prompts/cue<br>**9.1** Credible source<br>**10.4** Social reward	6-month follow-up:<br>Mean weight (kg)<br>• **Intervention: -2.5**<br>• Control: -2.1<br>• Between groups	6-month follow-up:<br>Mean ALT (IU/L)<br>• **Intervention: -8.1**<br>• Control: -5.6<br>• Between groups<br>Mean AST (IU/L)<br>• **Intervention: -4.1**<br>• Control: -0.9<br>• Between groups<br>Mean GGT (IU/L)<br>• **Intervention: -12.2**<br>• Control: -4<br>• Between groups
**Lim *et al.* (2020)**[37]<br>Singapore<br>RCT	Total sample: 108<br>• Intervention: 55 enrolled; 55 analysed<br>• Control: 53 enrolled; 53 analysed<br>Age (years), mean (SD):<br>• Intervention: 46.8 (11.1)<br>• Control: 46.2 (10.1)<br>Female, n (%):<br>• Intervention: 23 (42)<br>• Control: 17 (32)<br>BMI (kg/m^2), mean (SD):<br>• Intervention: 30.1 (4)<br>• Control: 30.8 (4.8)	Intervention name: Nutritionist Buddy (nBuddy)<br>Intervention content: Self-regulation tools (food diary, step recorder and goal setting, weight logging); feedback from dietitians; peer support chat channel; information (video education, dietary recommendations); daily prompts to log meals<br>Theoretical underpinning: Obesity-Related Behavioural Intervention Trials	Mode of delivery: app<br>Duration: 6 months<br>Frequency: continuous access	Standard care/control/comparator content: standard care advice on dietary and physical activity modification as per guidelines at a single face-to-face session	**2.2** Feedback on behaviour<br>**2.3** Self-monitoring of behaviour<br>**2.7** Feedback on outcome(s) of behaviour<br>**3.1** Social support (unspecified)<br>**7.1** Prompts/cues<br>**9.1** Credible source<br>**10.4** Social reward	3-month follow-up:<br>Mean weight (kg)<br>• Intervention: -3.2<br>• Control: -0.8<br>• **Between groups**<br>6-month follow-up:<br>Mean weight (kg)<br>• Intervention: -3.2<br>• Control: -0.5<br>• **Between groups**	3-month follow-up:<br>Mean ALT (IU/L)<br>• Intervention: -37.2<br>• Control: -20.7<br>• **Between groups**<br>Mean AST (IU/L)<br>• Intervention: -20.2<br>• Control: -11<br>• Between groups<br>6-month follow-up:<br>Mean ALT (IU/L)<br>• Intervention: -33.5<br>• Control: -11.5<br>• **Between groups**<br>Mean AST (IU/L)<br>• Intervention: -17.4IU/L<br>• Control: -7.4IU/L<br>**Between groups**

(*continued on next page*)

Table 1. (*continued*)

Study ID, country of origin, study design	Sample details and demographics	Intervention group	Intervention mode of delivery, duration, and frequency of access	Standard care/ control/comparator group	BCTs coded in intervention group over and above standard care/control/comparator group	Change in weight and metabolic outcomes from baseline to all follow-up points*	Change in liver-related outcome measures from baseline to all follow-up points*
**Mazzotti *et al.* (2018)**[38]<br>Italy<br>Two-group non-RCT	Total sample: 716<br>• Intervention: 278 enrolled; 211 analysed at 6 months; 160 analysed at 12 months; 118 analysed at 24 months<br>• Control: 438 enrolled; 383 analysed at 6 months; 352 analysed at 12 months; 301 analysed at 24 months<br>Age (years), mean (SD):<br>• Intervention: 46.0 (11.5)<br>• Control: 55.1 (12.3)<br>Female (%):<br>• Intervention: 33.1<br>• Control: 55<br>BMI (kg/m^2), mean (SD):<br>• Intervention: 33.7 (6)<br>• Control: 33.2 (5.2)	Intervention name: No name reported<br>Intervention content: educational slides (energy balance, nutrients and weight monitoring; alimentary pyramid and portion size; food shopping, food labels; physical activity, when and how much); tests of information; gamification; ability to send food diaries to clinical centre<br>Theoretical underpinning: not reported	Mode of delivery: Web<br>Duration: 5-weeks<br>Frequency: 1 session/ week with content accessible continuously thereafter	Standard care/control/ comparator content: 1 120 min group counselling session/week for 5-weeks chaired by physicians and dietitians. Educational slides (energy balance, nutrients and weight monitoring; alimentary pyramid and portion size; food shopping, food labels; physical activity, when and how much); behavioural strategies for weight loss maintenance	**2.1** Monitoring of behaviour by others without feedback	6-month follow-up:<br>Mean weight (%)<br>• Intervention: -3.4<br>• Control: -3.1<br>• **Between groups**<br>Mean TGs (mg/dl)<br>• Intervention: -15.6<br>• Control: -23.5<br>• Between groups<br>12-month follow-up:<br>Mean weight (%)<br>• Intervention: -4.9<br>• Control: -4<br>• **Between groups**<br>Mean TGs (mg/dl)<br>• Intervention: -24.9<br>• Control: -27.9<br>• Between groups<br>24-month follow-up:<br>Mean weight (%)<br>• Intervention: -5.5<br>• Control: -4.2<br>• **Between groups**<br>Mean TGs (mg/dl)<br>• Intervention: -23.5<br>• Control: -26.6<br>• Between groups	6-month follow-up:<br>Mean ALT (IU/L)<br>• Intervention: -14.3<br>• Control: -17.1<br>• Between groups<br>Mean GGT(IU/L)<br>• Intervention: -7.4<br>• Control: -12.6<br>• **Between groups**<br>12-month follow-up:<br>Mean ALT (IU/L)<br>• Intervention: -18.5<br>• Control: -18.5<br>• Between groups<br>Mean GGT(IU/L)<br>• Intervention: -16.4<br>• Control: -15.2<br>• Between groups<br>24-month follow-up:<br>Mean ALT (IU/L)<br>• Intervention: -22<br>• Control: -19.4<br>• Between groups<br>Mean GGT(IU/L)<br>• Intervention: -23.5<br>• Control: -16.1<br>• **Between groups**
**Motz *et al.* (2021)**[39]<br>USA<br>Single-group pre–post study	Total sample: 3<br>• Intervention: 3 enrolled; 3 analysed<br>Age (years), mean (SD):<br>• Intervention: 52 (14)<br>Female, n (%):<br>• Intervention: 3 (100)<br>BMI (kg/m^2), mean (SD):<br>• Intervention: 31.9 (5.1)	Intervention name: NASHFit<br>Intervention content: supervised aerobic exercise; provision of fitness trackers; feedback from exercise physiologist; dietary counselling and nutritional feedback<br>Theoretical underpinning: not reported	Mode of delivery: video software<br>Duration: 5 months<br>Frequency: five 30-min supervised exercise sessions/week	Standard care/control/ comparator content: N/A	**2.2** Feedback on behaviour<br>**2.6** Biofeedback<br>**3.1** Social support (unspecified)<br>**9.1** Credible source<br>**12.5** Adding objects to the environment	5-month follow-up:<br>Mean weight (%)<br>• Intervention: -5.1<br>Mean HbA_{1c} (%)<br>• Intervention: -0.5	5-month follow-up:<br>Mean ALT (IU/L)<br>• Intervention: -12.5<br>Mean AST (IU/L)<br>• Intervention: -8.5<br>Mean fat %: MRI-PDFF (%)<br>• Intervention: -35.1

(*continued on next page*)

Table 1. (*continued*)

Study ID, country of origin, study design	Sample details and demographics	Intervention group	Intervention mode of delivery, duration, and frequency of access	Standard care/ control/compar- ator group	BCTs coded in intervention group over and above stan- dard care/control/compar- ator group	Change in weight and metabolic outcomes from baseline to all follow-up points*	Change in liver-related outcome measures from baseline to all follow- up points*
**Sato *et al.*, (2023)**[40]<br>Japan<br>Single-group pre–post study	Total sample: 20<br>• Intervention: 20 enrolled; 19 analysed<br>Age (years), mean (SD):<br>• Intervention: 52.16 (10.77)<br>Female, n (%):<br>• Intervention: 9 (47.4)<br>BMI (kg/m^2), mean (SD):<br>• Intervention: 32.04 (4.19)	Intervention name: NASH App<br>Intervention content: education; app-based support and instruction to implement lifestyle advice; self-planning and evaluation to encourage maintenance of behavioural change<br>Theoretical underpinning: not reported	Mode of delivery: app<br>Duration: 11 months<br>Frequency: continuous access	Standard care/control/ comparator content: N/A	**2.4** Self-monitoring of outcome(s) of behaviour<br>**3.1** Social support (unspecified)<br>**4.1** Instruction on how to perform the behaviour	6-month follow-up:<br>Mean weight (kg)<br>• **Intervention: -6**<br>Mean HbA_{1c} (%)<br>• Intervention: -0.26<br>Mean LDL (mg/dl)<br>• Intervention: +0.37<br>Mean TGs (mg/dl)<br>• Intervention: -1.32<br>11-month follow-up:<br>Mean weight (kg)<br>• **Intervention: -7.3**<br>Mean HbA_{1c} (%)<br>• Intervention: -0.27<br>Mean LDL (mg/dl)<br>• Intervention: -6<br>Mean TGs (mg/dl)<br>**Intervention: -19.89**	6-month follow-up:<br>Mean ALT (IU/L)<br>• **Intervention: -40.84**<br>Mean AST (IU/L)<br>• **Intervention: -18.84**<br>Mean ALP (IU/L)<br>• **Intervention: -8.84**<br>Mean GGT (IU/L)<br>• **Intervention: -23.89**<br>11-month follow-up:<br>Mean ALT (IU/L)<br>• **Intervention: -49.53**<br>Mean AST (IU/L)<br>• **Intervention: -23.05**<br>Mean ALP (IU/L)<br>• **Intervention: -10.63**<br>Mean GGT (IU/L)<br>• **Intervention: -28.63**
**Stine *et al.* (2023)**[41]<br>USA<br>Pilot RCT	Total sample: 40<br>• Intervention: 20 enrolled; 20 analysed<br>• Control: 20 enrolled; 20 analysed<br>Age (years), mean (SD):<br>• Intervention: 53.3 (13.3)<br>• Control: 50.4 (12.1)<br>Female, n (%):<br>• Intervention: 12 (60%)<br>• Control: 17 (85%)<br>BMI (kg/m^2), mean (SD):<br>• Intervention: 36.1 (6.4)<br>• Control: 36.3 (6.5)	Intervention name: Noom Weight<br>Intervention content: self-monitoring and feedback on food, exercise, weight; access to coach and support group; education on nutrition, activity, and behavioural change; provision of electronic scales and FitBit<br>Theoretical underpinning: Cognitive Behavioural Therapy; Acceptance and Commitment Therapy; Dialectical Behaviour Therapy	Mode of delivery: app<br>Duration: 4 months<br>Frequency: continuous access	Standard care/control/ comparator content: standard in-person counselling; provision of electronic scales and FitBit	**2.2** Feedback on behaviour<br>**2.3** Self-monitoring of behaviour<br>**2.4** Self-monitoring of outcome(s) of behaviour<br>**2.7** Feedback on outcome(s) of behaviour<br>**11.2** Reduce negative emotions	4-month follow-up:<br>Mean weight (kg)<br>• **Intervention: -8.5**<br>• Control: +0.4<br>• **Between groups**	4-month follow-up:<br>Mean ALT (IU/L)<br>• Intervention: -9.2<br>• Control: -5.2<br>• Between groups<br>Mean AST (IU/L)<br>• Intervention: -4.3<br>• Control: -3.9<br>• Between groups<br>Mean ALP (IU/L)<br>• Intervention: +5.5<br>• Control: +11.3<br>• Between groups

(*continued on next page*)

Table 1. (*continued*)

Study ID, country of origin, study design	Sample details and demographics	Intervention group	Intervention mode of delivery, duration, and frequency of access	Standard care/control/comparator group	BCTs coded in intervention group over and above standard care/control/comparator group	Change in weight and metabolic outcomes from baseline to all follow-up points*	Change in liver-related outcome measures from baseline to all follow-up points*
**Tincopa *et al.* (2022)**[42]<br>USA<br>Single-group pre–post study	Total sample: 40<br>• Intervention: 40 enrolled; 33 analysed<br>Age (years), median (IQR):<br>• Intervention: 53 (43–61)<br>Female, n (%):<br>• Intervention: 16 (48.5%)<br>BMI (kg/m^2), median (IQR):<br>• Intervention: 33.6 (29.6–35.3)	Intervention name: No name reported<br>Intervention content: Provision of FitBit; self-management of physical activity; feedback on physical activity with personalised step goals; motivational messages via email<br>Theoretical underpinning	Mode of delivery: app and email<br>Duration: 6 months<br>Frequency: continuous access to app; email communication no more than once weekly for first 3 months and then bi-weekly for the remaining 3 months	Standard care/control/comparator content: N/A	**2.2** Feedback on behaviour<br>**2.3** Self-monitoring of behaviour<br>**3.1** Social support (unspecified)<br>**7.1** Prompts/cues<br>**12.5** Adding objects to the environment	6-month follow-up:<br>Median weight (lbs)<br>• Intervention: +7<br>Median HbA$_{1c}$ (mmol/L)<br>• **Intervention: -0.1**<br>Median HDL (mg/dl)<br>• **Intervention: +5.5**<br>Median LDL (mg/dl)<br>• **Intervention: -19.5**<br>Median TGs (mg/dl)<br>**Intervention: -10.5**	6-month follow-up:<br>Median ALT (IU/L)<br>• Intervention: -11<br>Median fibrosis: LSM (kPa)<br>• Intervention: +0.6<br>Mean steatosis: CAP (dB/m)<br>• Intervention: -22.5

*Change calculated by subtracting the mean/median value at follow-up from the mean/median value at baseline. Statistically significant (p <0.05) results are highlighted in bold. ALP, alkaline phosphatase; ALT, alanine transaminase; AST, aspartate transaminase; BCT, behaviour change technique; CAP, controlled attenuation parameter; GGT, gamma-glutamyl transaminase; HDL, high-density lipoprotein cholesterol; LDL, low-density lipoprotein cholesterol; LSM, liver stiffness measurement; MRI-PDFF, magnetic resonance imaging proton density fat fraction; RCT, randomised controlled trial; TG, triglyceride.

<0.001).[33] No statistically significant differences from baseline to post-intervention follow-up were reported for the second study.[39]

Change in metabolic outcomes

HbA$_{1c}$ (mmol/L or %)

Four single-group pre–post studies assessed HbA$_{1c}$ as an outcome.[33,39,40,42] Of these studies, two reported statistically significant reductions of -0.5 mmol/L (p = 0.03)[33] and -0.1 mmol/L (p <0.01)[42] from baseline to post-intervention follow-up. Two studies reported no statistically significant changes.[39,40]

Lipid profile (TGs, LDL, HDL, and total cholesterol)

Six studies (three single-group pre–post studies,[33,40,42] two RCTs,[32,34] and one two-group non-RCT[38]) assessed TGs as an outcome. Four studies reported a statistically significant improvement in TGs within intervention groups, with a mean reduction of -12.7 mg/dl (SD 6.28 mg/dl).[32,33,40,42] Of these four studies, the three single-group pre–post studies showed statistically significant reductions of -0.2 mmol/L (p = 0.003),[33] -10.5 mg/dl (p = 0.03),[42] and -19.9 mg/dl (p = 0.01)[40] from baseline to post-intervention follow-up, whereas the RCT[32] showed a statistically significant reduction of -17 mg/dl (p = 0.04) in the intervention group but found no differences between groups. The second RCT[34] reported no statistically significant differences within or between groups. The two-group non-RCT[38] showed no statistically significant differences between groups at any time point.

Four studies (three single-group pre–post studies[33,40,42] and one RCT[34]) reported LDL as an outcome. One study[42] reported a statistically significant reduction of -19.5 mg/dl (p <0.01), whereas the remaining three studies[33,34,40] showed no statistically significant changes from baseline to post-intervention follow-up, or between intervention and control groups.

Similarly, four studies (two single-group pre–post studies[33,42] and two RCTs[32,34]) assessed HDL as an outcome. One study showed a statistically significant increase of +5.5 mg/dl from baseline to post-intervention follow-up (p <0.01),[42] whereas no statistically significant changes were observed in the remaining three studies [32–34].

Two studies (one single-group pre–post study[33] and one RCT[34]) assessed total cholesterol (mmol/L) as an outcome. Neither reported statistically significant changes from baseline to post-intervention follow-up or between intervention and control groups.

Change in lifestyle behaviours

Five studies (three single-group pre–post studies,[33,39,42] one RCT,[35] and one non-RCT[38]) assessed whether changes in physical activity/physical fitness were observed following engagement with the digital intervention. Two studies (one RCT,[35] and one non-RCT[38]) assessed metabolic equivalents (METs) as an outcome. Both studies reported no statistically significant changes in METs from baseline to post-intervention follow-up or between groups. Two single-group studies[33,42] assessed steps/day as an outcome. One study reported a

statistically significant increase of +1,579 steps/day from pre- to post-intervention (p = 0.02),[33] whereas the other reported a decrease of -751 steps/day that was not statistically significant.[42] One single-group study[39] reported an increase of +9.9 ml/kg/min in peak oxygen uptake (VO_2 peak), although this was not assessed for statistical significance. Another single-group study[42] assessed the 6-minute walk test as an outcome and reported a statistically significant increase of 140 feet from baseline to post-intervention follow-up (p <0.01). Only one study (a non-RCT[38]) assessed whether changes in dietary behaviours were observed after engagement with the intervention. This study assessed caloric intake (kcal/day) and reported a decrease in kcal/day in the intervention group (-273 kcal/day) and the comparator group (-193 kcal/day), but these were not statistically significant.

Active intervention ingredients and intervention promise

Overall, 22 distinct BCTs were identified across all digital interventions (see Table 2). Each intervention included at least one BCT, and the mean number of BCTs used within interventions was 5 (SD 3.6; median 5; IQR 3-7; range 1–13). The most frequently coded BCTs were 'social support (unspecified)' (n = 8), 'feedback on behaviour' (n = 7), 'self-monitoring of behaviour' (n = 6), and 'prompts/cues' (n = 6). Table 2 presents the specific BCTs coded within each intervention in accordance with a valid and reliable behaviour change taxonomy.[24]

Table 3 presents promise ratios for the active ingredients (intervention features and BCTs) of the included interventions that are associated with improvements in weight/metabolic outcomes and/or liver-related outcome measures.

App-based delivery was the only mode of delivery that was associated with improvements in both weight/metabolic outcomes and liver-related outcome measures (promise ratios 4.00). In relation to improvements in liver-related outcome measures, SMS/instant messaging was used in promising interventions only (n = 2). However, SMS/instant messaging was not associated with improvements in weight/metabolic outcomes. Similarly, in relation to improvements in weight/metabolic outcomes, app-based delivery in conjunction with another mode of communication (*i.e.* e-mail or SMS) was used in promising interventions only (n = 2) but was not associated with improvements in liver-related outcome measures.

An intervention duration of ≥6 months was associated with improvements in both weight/metabolic outcomes and liver-related outcome measures (promise ratios 5.00), whereas an intervention duration of <6 months was not associated with improvements in either outcome. Continuous access was associated with improvements in liver-related outcomes (promise ratio 3.00). Similarly, in relation to improvements in weight/metabolic outcomes, continuous access to intervention content was used in promising interventions only (n = 4). A frequency of access three to seven times per week was also associated with improvements in liver-related outcome measures (promise ratio 2.00). Continuous access with provision of an exercise session and/or meal replacement one to seven times per week was not associated with improvements in weight/metabolic outcomes. In relation to improvements in liver-related outcome measures, continuous access with provision of an exercise session and/or meal replacement one to seven times per week was present in promising interventions only (n = 2).

Of the 22 BCTs identified across all interventions, 13 were used by at least two interventions. Seven BCTs were associated with improvements in weight/metabolic outcomes, and six BCTs were associated with improvements in liver-related outcomes. Examples of BCTs associated with improvements in weight/metabolic outcomes include 'self-monitoring of behaviour' (promise ratio 5.00), 'social support (unspecified)' (promise ratio 3.00), and 'feedback on behaviour' (promise ratio 2.50). Examples of BCTs associated with improvements in liver-related outcome measures were 'prompts/cues' (promise ratio 5.00) and 'social support (unspecified)' (promise ratio 3.00). Overall, six BCTs were associated with improvements in both weight/metabolic outcomes and liver-related outcome measures. They were 'self-monitoring of behaviour', 'self-monitoring of outcome(s) of behaviour', 'feedback on outcome(s) of behaviour', 'social support (unspecified)', 'prompts/cues', and 'credible source'.

Methodological quality assessment

A summary of methodological quality assessment for all 11 included studies is presented in Supplementary file 7 in accordance with the assessment tool used.

Six studies[32,34–37,41] were assessed using the ROB-2 tool.[25] These studies were rated as having 'some concerns' because of a lack of information about pre-specified analysis plans. One study[38] was assessed using the ROBINS-I tool[26] and was rated as 'low' risk of bias. Four studies[33,39,40,42] were assessed using the NHLBI pre–post tool.[27] Of these, one study[40] was rated as 'good' quality. Two studies[33,42] were rated as 'fair' because of a lack of reporting concerning whether the sample size was sufficiently large to provide confidence in the findings. One study[39] was rated as 'poor' quality because of a lack of clarity about the reporting of study objectives and a small sample size.

Discussion

This is the first systematic review and meta-analysis to determine the effectiveness and active ingredients of digital behaviour change interventions for improving weight and liver-related outcome measures in patients diagnosed with MASLD.

A total of 11 studies reporting on digital behaviour change interventions met the eligibility criteria. Findings of the meta-analysis involving five RCTs for AST and six RCTs for ALT and weight indicate that, overall, digital behavior change interventions are not effective for improving weight or liver-related outcome measures. Findings from pre–post studies and one non-RCT showed statistically significant improvements in weight (n = 3 studies) and improvements in weight that were not statistically significant (n = 1 study). Findings from pre–post and non-RCT studies showed statistically

Table 2. BCTs coded within each digital intervention using BCTTv1.

BCTs (BCT taxonomy identifiers and name)	Axley *et al.* (2018)[32]	Björnddottir *et al.* (2024)[33]	Cho *et al.* (2024)[34]	Kaewdech *et al.* (2024)[35]	Kwon *et al.* (2024)[36]	Lim *et al.* (2020)[37]	Mazzotti *et al.* (2018)[38]	Motz *et al.* (2021)[39]	Sato *et al.* (2023)[40]	Stine *et al.* (2023)[41]	Tincopa *et al.* (2022)[42]
1.1 Goal setting (behaviour)			X		X						
1.2 Problem solving	X										
1.3 Goal setting (outcome)	X				X						
1.4 Action planning			X		X						
1.5 Review behaviour goal(s)					X						
1.7 Review outcome goal(s)					X						
2.1 Monitoring of behaviour by others without feedback							X				
2.2 Feedback on behaviour		X	X		X	X		X		X	X
2.3 Self-monitoring of behaviour		X	X		X	X				X	X
2.4 Self-monitoring of outcome(s) of behaviour			X						X	X	
2.6 Biofeedback								X			
2.7 Feedback on outcome(s) of behaviour			X			X				X	
3.1 Social support (unspecified)	X	X	X		X	X		X	X		X
4.1 Instruction on how to perform the behaviour					X				X		
6.1 Demonstration of the behaviour					X						
7.1 Prompts/cues	X		X	X	X	X					X
8.3 Habit formation			X								
8.4 Habit reversal			X								
9.1 Credible source					X	X		X			
10.4 Social reward					X	X					
11.2 Reduce negative emotions										X	
12.5 Adding objects to the environment			X					X			X

BCT, behaviour change techniques; BCTTv1, Behaviour Change Techniques Taxonomy version 1.

Table 3. Promise ratios for active ingredients of digital behavioural interventions.

	Associated with improvement in weight/metabolic outcomes				Associated with improvement in liver-related outcome measures			
**Active ingredient of digital behavioural interventions**	**n**	**Presence in very/quite promising interventions**	**Presence in non-promising interventions**	**Promise ratio**	**n**	**Presence in very/quite promising interventions**	**Presence in non-promising interventions**	**Promise ratio**
**Mode of delivery**								
App	5	**4**	**1**	**4.00**	5	**4**	**1**	**4.00**
SMS/instant messaging	2	1	1	1.00	2	**2**	-	–
App and additional communication (*i.e.* email/SMS)	2	**2**	-	–	2	1	1	1.00
**Duration of intervention (months)**								
<6	5	3	2	1.50	5	3	2	1.50
≥6	6	**5**	**1**	**5.00**	6	**5**	**1**	**5.00**
**Intervention frequency**								
Continuous access	4	**4**	-	–	4	**3**	**1**	**3.00**
3–7 times/week	3	1	2	0.50	3	**2**	**1**	**2.00**
Continuous access with additional communication 1–7 times/week	2	**2**	-	-	2	1	1	1.00
Continuous access with provision of exercise session/meal replacement 1–7 times/week	2	1	1	1.00	2	**2**	-	–
**BCTs***								
1.1 Goal setting (behaviour)	2	1	1	1.00	2	**2**	-	–
1.3 Goal setting (outcome)	2	**2**	-	–	2	**2**	-	–
1.4 Action planning	2	1	1	1.00	2	**2**	-	–
2.2 Feedback on behaviour	7	**5**	**2**	**2.50**	7	4	3	1.33
2.3 Self-monitoring of behaviour	6	**5**	**1**	**5.00**	6	**4**	**2**	**2.00**
2.4 Self-monitoring of outcome(s) of behaviour	3	**2**	**1**	**2.00**	3	**2**	**1**	**2.00**
2.7 Feedback on outcome(s) of behaviour	3	**2**	**1**	**2.00**	3	**2**	**1**	**2.00**
3.1 Social support (unspecified)	8	**6**	**2**	**3.00**	8	**6**	**2**	**3.00**
4.1 Instruction on how to perform the behaviour	2	**2**	-	–	2	**2**	-	–
7.1 Prompts/cues	6	**4**	**2**	**2.00**	6	**5**	**1**	**5.00**
9.1 Credible source	3	**2**	**1**	**2.00**	3	**2**	**1**	**2.00**
10.4 Social reward	2	**2**	-	–	2	**2**	-	–
12.5 Adding objects to the environment	3	1	2	0.50	3	1	2	0.50

Promise ratio denotes the number of very or quite promising interventions in which an intervention ingredient featured, divided by the number of non-promising interventions in which it featured. Promise ratios are only calculable for ingredients used in both promising and non-promising interventions. Where functions or techniques were used only in (two or more) promising interventions (promise ratio = ∞), the number of interventions in which they were used was reported instead of the ratio. Ratios in bold denote ingredients associated with a promise ratio of 2 or above, or used exclusively in promising interventions and featuring in at least two interventions.[43] *Number reported with BCT corresponds to the identifying number reported in the BCTTv1.[24] BCT, behaviour change technique; BCTTv1, Behaviour Change Techniques Taxonomy version 1.

significant improvements in ALT (n = 1 study), improvements in ALT that were not statistically significant (n = 3 studies), and worsening of ALT that was not statistically significant (n = 1 study). Similarly, findings from pre–post and non-RCT studies showed statistically significant improvements in AST (n = 1 study), improvements in AST that were not statistically significant (n = 1 study), and worsening of AST that was not statistically significant (n = 1 study). Promise analysis conducted on data from studies that reported statistically significant findings in relation to weight/metabolic outcomes and/or liver-related outcome measures identified specific features and content of digital interventions that likely moderated the effectiveness of the interventions. The findings of this current review do not support the findings of a previous review published in 2024 that reported digital interventions as effective for weight loss.[21] However, the meta-analysis presented in the 2024 systematic review included data from single-group studies and intervention arms of RCTs only. This likely contributed to the larger effect sizes reported compared with the current review.

All studies included in the current review differed in terms of their mode of delivery, duration, frequency of intervention delivery, and specific BCTs included within them. App-based delivery and an intervention duration of ≥6 months were both associated with improvements in weight/metabolic outcomes and liver-related outcome measures. Similarly, six distinct BCTs were identified as promising for improving weight/metabolic outcomes and liver-related outcome measures. Of the six BCTs that showed promise, many are associated with self-regulation of behaviours, including diet and physical activity (*i.e.* 'self-monitoring of behaviour' and 'prompts/cues'). The identification of these BCTs emphasises the important role that self-regulation plays in improving MASLD-related outcomes. This finding supports several health behaviour change theories that position self-regulation as a central mechanism of action in health behaviour change.[44,45] Of the 11 studies included in this review, only two reported the use of theory in intervention development.[37,41] Theory provides a structured and explicit framework for designing, evaluating, and optimising interventions. This can inform intervention content, enhance effectiveness by ensuring interventions target appropriate behavioural determinants, provide a framework for evaluation, and facilitate replication. A substantial body of evidence has highlighted the effectiveness of theory-based interventions for targeting change in behaviour[46,47] The Medical Research Council framework for the Development and Evaluation of Complex Interventions suggests that complex interventions should be developed with reference to theory and systematically following phases of iterative development (*i.e.* considering core elements of the intervention, assessment of feasibility, consideration of implementation, and evaluation).[48] Digital behaviour change interventions are often complex and multi-faceted; therefore, it is recommended that they follow a systematic, theory-informed development process, taking into account the complexity of the intended intervention.[49]

Several systematic reviews have been conducted to determine the efficacy of digital behaviour change interventions for other long-term health conditions (*e.g.* type 2 diabetes) and have reported BCTs or combinations of BCTs (*i.e.* social support and feedback on behaviour) positively associated with improvements in clinical outcome measures, similar to those reported by this review.[50,51] The findings of the current review are important as they highlight the specific active ingredients that could be central to the management of MASLD and for improving MASLD-related outcomes in future research or clinical practice. The success of specific BCTs (and other intervention features, including mode of digital intervention delivery, frequency of delivery, and duration of provision) for improving clinical outcome measures in other conditions further emphasises the utility of incorporating promising active ingredients into interventions to facilitate improvements in MASLD-related outcomes.

Importantly, only five of the studies included in this systematic review explicitly measured and reported data on changes in lifestyle behaviours.[33,35,38,39,42] This is despite most interventions aiming to change lifestyle behaviours (*i.e.* diet and physical activity) to positively impact weight or liver-related outcome measures. Therefore, it cannot be definitively concluded that changes in weight and liver-related outcome measures were a consequence of any changes in lifestyle behaviours targeted by the intervention. Future studies should explicitly measure whether the intervention leads to changes in lifestyle behaviours that precede changes in weight and liver-related outcome measures.

Strengths and limitations

A strength of this review is the use of robust methodology and data analysis to determine the effectiveness of digital behaviour change interventions for improving weight and liver-related outcome measures and to identify promising intervention features and content. This review is the first of its kind to conduct these analyses in the context of MASLD and provides important information to inform the development and optimisation of future digital interventions. Although it was not possible to conduct a meta-analysis of all included studies owing to study design and heterogeneity of outcomes reported, an alternative approach was used (i.e., SWiM) to ensure that data of single-group studies and non-RCTs were examined consistently, robustly, and transparently.

A potential limitation of this review is the low number of RCTs included, and those were largely feasibility and pilot studies with small sample sizes. This indicates the relative infancy of this field, which demonstrates the need for large, controlled studies to establish a more robust evidence base. Similarly, several studies included were rated as having 'some concerns' following risk of bias assessment. This may have implications for the reliability of the conclusions drawn.

Conclusions

The findings of this systematic review indicate that digital behaviour change interventions, overall, are not effective for improving weight and liver-related outcome measures in patients with MASLD. However, of those individual studies that did report statistically significant improvements in weight/metabolic outcomes and liver-related outcome measures, we were able to identify a range of promising active intervention ingredients, which provide some guidance for the development of future digital behaviour change interventions or the optimisation of existing interventions. It is important to refer to

intervention features and content that have been shown to be associated with effectiveness in other intervention studies, in related clinical fields, and in this review. In conclusion, although the findings of this review indicate that digital behaviour change interventions are not effective for improving weight and liver-related outcomes measures overall, it is clear that these interventions do work for some people, and it is important to identify who those people are. Moreover, findings indicate that those interventions that do lead to effective changes in weight and/or liver-related outcome measures share some common features and content. These findings can inform future digital behaviour change interventions for MASLD.

Affiliations

[1]*School of Health and Life Sciences, Teesside University, Middlesbrough, UK;* [2]*School of Social Sciences, Humanities and Law, Teesside University, Middlesborough, UK;* [3]*NIHR Newcastle Patient Safety Research Collaborative, Newcastle University, Newcastle upon Tyne, UK;* [4]*NIHR Newcastle Biomedical Research Centre, Newcastle upon Tyne Hospitals NHS Foundation Trust, Newcastle upon Tyne, UK;* [5]*Liver Unit, Newcastle upon Tyne Hospitals NHS Foundation Trust, Newcastle upon Tyne, UK;* [6]*Translational and Clinical Research Institute, Faculty of Medical Sciences, Newcastle University, Newcastle upon Tyne, UK*

Abbreviations

ALP, alkaline phosphatase; ALT, alanine transaminase; AST, aspartate transaminase; BCT, behaviour change technique; BCTTv1, Behaviour Change Techniques Taxonomy version 1; CAP, controlled attenuation parameter; DPP, Diabetes Prevention Programme; GGT, gamma-glutamyl transaminase; HCP, healthcare professional; HDL, high-density lipoprotein cholesterol; LDL, low-density lipoprotein cholesterol; LSM, liver stiffness measurement; MASH, metabolic dysfunction-associated steatohepatitis; MASLD, metabolic dysfunction-associated steatotic liver disease; METS, metabolic equivalents; MRI-PDFF, magnetic resonance imaging proton density fat fraction; NHLBI, National Heart, Lung and Blood Institute; NHS, National Health Service; PRISMA, Preferred Reporting Items for Systematic reviews and Meta-Analyses; RCT, randomised controlled trial; ROB-2, Revised Cochrane Risk of Bias Tool; ROBINS-I, Risk Of Bias In Non-randomised Studies of Interventions; SWiM, Synthesis Without Meta-Analysis; TG, triglyceride; WMD, weighted mean difference.

Financial support

This systematic review was conducted as part of a Teesside University fully funded PhD studentship undertaken by HS.

Conflicts of interest

SMc has received consultancy/speakers fees from Abbvie, Allergan, BMS, Gilead, Intercept, MSD, Novo Nordisk, Norgine, Novartis, and Sequana. The remaining authors declare no conflicts of interest relating to this work.

Please refer to the accompanying ICMJE disclosure forms for further details.

Authors' contributions

Conceived the idea for this review: HS, LA, SMc, KH. Developed the review protocol: HS, LA, SMc, KH, AI. Developed the search strategy and conducted database searching: HS. Conducted stage 1 and stage 2 screening: HS, RL. Conducted data extraction, with input from all authors when required: HS. Coded the presence of behaviour change techniques: HS, KA. Conducted methodological quality assessment: HS, LA, KA, MC. Meta-analysed/synthesised data: HS. Provided support in meta-analysis/synthesis of data: LA, KH, SMc. Drafted the manuscript: HS. Revised the manuscript for important intellectual content and approved the final version: all authors.

Data availability

The data set from this systematic review and meta-analysis is available upon reasonable request.

Supplementary data

Supplementary data to this article can be found online at https://doi.org/10.1016/j.jhepr.2025.101507.

References

Author names in bold designate shared co-first authorship

[1] Rinella ME, Lazarus JV, Ratziu V, et al. A multisociety Delphi consensus statement on new fatty liver disease nomenclature. Hepatology 2023;78:1966–1986.

[2] Miao L, Targher G, Byrne CD, et al. Current status and future trends of the global burden of MASLD. Trends Endocrinol Metab 2024;35:697–707.

[3] European Association for the Study of the Liver. EASL–EASD–EASO Clinical Practice Guidelines on the management of metabolic dysfunction-associated steatotic liver disease (MASLD). J Hepatol 2024;81:492–542.

[4] Anstee QM, Hallsworth K, Lynch N, et al. Real-world management of non-alcoholic steatohepatitis differs from clinical practice guideline recommendations and across regions. JHEP Rep 2022;4:100411.

[5] Vilar-Gomez E, Martinez-Perez Y, Calzadilla-Bertot L, et al. Weight loss through lifestyle modification significantly reduces features of nonalcoholic steatohepatitis. Gastroenterology 2015;149:367–378.

[6] Koutoukidis DA, Koshiaris C, Henry JA, et al. The effect of the magnitude of weight loss on non-alcoholic fatty liver disease: a systematic review and meta-analysis. Metabolism 2021;115:154455.

[7] Kalmpourtzidou A, Eilander A, Talsma EF. Global vegetable intake and supply compared to recommendations: a systematic review. Nutrients 2020;12:1558.

[8] Guthold R, Stevens GA, Riley LM, et al. Worldwide trends in insufficient physical activity from 2001 to 2016: a pooled analysis of 358 population-based surveys with 1·9 million participants. Lancet Glob Health 2018;6:e1077–e1086.

[9] Middleton KR, Anton SD, Perri MG. Long-term adherence to health behavior change. Am J Lifestyle Med 2013;7:395–404.

[10] Deslippe AL, Soanes A, Bouchaud CC, et al. Barriers and facilitators to diet, physical activity and lifestyle behavior intervention adherence: a qualitative systematic review of the literature. Int J Behav Nutr Phys Act 2023;20:14.

[11] Kelly S, Martin S, Kuhn I, et al. Barriers and facilitators to the uptake and maintenance of healthy behaviours by people at mid-life: a rapid systematic review. PLoS One 2016;11:e0145074.

[12] Avery L, Exley C, McPherson S, et al. Lifestyle behavior change in patients with nonalcoholic fatty liver disease: a qualitative study of clinical practice. Clin Gastroenterol Hepatol 2017;15:1968–1971.

[13] Hallsworth K, Dombrowski SU, McPherson S, et al. Using the theoretical domains framework to identify barriers and enabling factors to implementation of guidance for the diagnosis and management of nonalcoholic fatty liver disease: a qualitative study. Transl Behav Med 2020;10:1016–1030.

[14] Tincopa MA, Wong J, Fetters M, et al. Patient disease knowledge, attitudes and behaviours related to non-alcoholic fatty liver disease: a qualitative study. BMJ Open Gastroenterol 2021;8:e000634.

[15] Eysenbach G. What is e-health? J Med Internet Res 2001;3:e20.

[16] Nkhoma DE, Soko CJ, Bowrin P, et al. Digital interventions self-management education for type 1 and 2 diabetes: a systematic review and meta-analysis. Comput Methods Programs Biomed 2021;210:106370.

[17] Wang Y, Xue H, Huang Y, et al. A systematic review of application and effectiveness of mHealth interventions for obesity and diabetes treatment and self-management. Adv Nutr 2017;8:449–462.

[18] Stratton E, Lampit A, Choi I, et al. Effectiveness of eHealth interventions for reducing mental health conditions in employees: a systematic review and meta-analysis. PLoS One 2017;12:e0189904.

[19] NHS England. NHS Diabetes Prevention Programme (NHS DPP). Available from: https://www.england.nhs.uk/diabetes/diabetes-prevention/Accessed 25 March 2025.

[20] Ross JAD, Barron E, McGough B, et al. Uptake and impact of the English National Health Service digital diabetes prevention programme: observational study. BMJ Open Diabetes Res Care 2022;10:e002736.

[21] Albhaisi S, Tondt J, Cyrus J, et al. Digital therapeutics lead to clinically significant body weight loss in patients with metabolic dysfunction–associated steatotic liver disease: a systematic review and meta-analysis. Hepatol Commun 2024;8:e0499.

[22] Smith H., Hallsworth K., McPherson S., et al. A systematic review to determine the effectiveness of digital behaviour change interventions for improving liver disease outcomes. PROSPERO 2023. CRD42023406827. Available from: https://www.crd.york.ac.uk/PROSPERO/view/CRD42023406827. Accessed 16 Aug 2025.
[23] Page MJ, Bossuyt PM, Boutron I, et al. The PRISMA 2020 statement: an updated guideline for reporting systematic reviews. BMJ 2021;372:n71.
[24] Michie S, Richardson M, Johnston M, et al. The Behavior Change Technique Taxonomy (v1) of 93 hierarchically clustered techniques: building an international consensus for the reporting of behavior change interventions. Ann Behav Med 2013;46:81–95.
[25] Higgins JPT, Altman DG, Gøtzsche PC, et al. The Cochrane Collaboration's tool for assessing risk of bias in randomised trials. BMJ 2011;343:d5928.
[26] Sterne JAC, Hernán MH, Reeves BC, et al. ROBINS-I: a tool for assessing risk of bias in non-randomised studies of interventions. BMJ 2016;355:i4919.
[27] National Heart, Lung and Blood Institute. Quality assessment tool for before–after (pre–post) studies with no control group. Available from: https://www.nhlbi.nih.gov/health-topics/study-quality-assessment-tools Accessed 25 March 2025.
[28] Higgins JPT, Thomas J, Chandler J, et al., editors. Cochrane handbook for systematic reviews of interventions. 2nd ed. Wiley: Hoboken: Cochrane Collaboration; 2019.
[29] Michie S, Fixsen D, Grimshaw JM, et al. Specifying and reporting complex behaviour change interventions: the need for a scientific method. Implement Sci 2009;4:40.
[30] Ashley K, Tang MY, Flynn D, et al. Identifying the active ingredients of training interventions for healthcare professionals to promote and support increased levels of physical activity in adults with heart failure: a systematic review. Health Psychol Rev 2024;18:319–340.
[31] Moore SA, Hrisos N, Flynn D, et al. How should long-term free-living physical activity be targeted after stroke? A systematic review and narrative synthesis. Int J Behav Nutr Phys Act 2018;15:1–19.
[32] Axley P, Kodali S, Kuo Y-F, et al. Text messaging approach improves weight loss in patients with nonalcoholic fatty liver disease: a randomized study. Liver Int 2018;38:924–931.
[33] Björnsdottir S, Ulfsdottir H, Gudmundsson EF, et al. User engagement, acceptability, and clinical markers in a digital health program for nonalcoholic fatty liver disease: prospective, single-arm feasibility study. JMIR Cardio 2024;8:e52576.
[34] **Cho E**, **Kim S**, Kim S, et al. The effect of mobile lifestyle intervention combined with high-protein meal replacement on liver function in patients with metabolic dysfunction-associated steatotic liver disease: a pilot randomized controlled trial. Nutrients 2024;16:2254.
[35] Kaewdech A, Assawasuwannakit S, Churuangsuk C, et al. Effect of smartphone-assisted lifestyle intervention in MASLD patients: a randomized controlled trial. Sci Rep 2024;14:13961.
[36] Kwon OY, Lee MK, Lee HW, et al. Mobile app-based lifestyle coaching intervention for patients with nonalcoholic fatty liver disease: randomized controlled trial. J Med Internet Res 2024;26:e49839.
[37] Lim SL, Johal J, Ong KW, et al. Lifestyle intervention enabled by mobile technology on weight loss in patients with nonalcoholic fatty liver disease: randomized controlled trial. JMIR Mhealth Uhealth 2020;8:e14802.
[38] Mazzotti A, Caletti MT, Brodosi L, et al. An internet-based approach for lifestyle changes in patients with NAFLD: two-year effects on weight loss and surrogate markers. J Hepatol 2018;69:1155–1163.
[39] Motz V, Faust A, Dahmus J, et al. Utilization of a directly supervised telehealth-based exercise training program in patients with nonalcoholic steatohepatitis: feasibility study. JMIR Form Res 2021;5:e30239.
[40] Sato M, Akamatsu M, Shima T, et al. Impact of a novel digital therapeutics system on nonalcoholic steatohepatitis: the NASH App clinical trial. Am J Gastroenterol 2023;118:1365–1372.
[41] Stine JG, Rivas G, Hummer B, et al. Mobile health lifestyle intervention program leads to clinically significant loss of body weight in patients with NASH. Hepatol Commun 2023;7:e0052.
[42] Tincopa MA, Lyden A, Wong J, et al. Impact of a pilot structured mobile technology based lifestyle intervention for patients with nonalcoholic fatty liver disease. Dig Dis Sci 2022;67:481–491.
[43] Gardner B, Smith L, Lorencatto F, et al. How to reduce sitting time? A review of behaviour change strategies used in sedentary behaviour reduction interventions among adults. Health Psychol Rev 2015;10:89–112.
[44] Suls J, Mogavero JN, Falzon L, et al. Health behaviour change in cardiovascular disease prevention and management: meta-review of behaviour change techniques to affect self-regulation. Health Psychol Rev 2020;14:43–65.
[45] Michie S, Johnston M. Theories and techniques of behaviour change: developing a cumulative science of behaviour change. Health Psychol Rev 2012;6:1–6.
[46] Knittle K, Nurmi J, Crutzen R, et al. How can interventions increase motivation for physical activity? A systematic review and meta-analysis. Health Psychol Rev 2018;12:211–230.
[47] Williams GC, McGregor H, Sharp D, et al. A self-determination multiple risk intervention trial to improve smokers' health. J Gen Intern Med 2006;21:1288–1294.
[48] Skivington K, Matthews L, Simpson SA, et al. A new framework for developing and evaluating complex interventions: update of Medical Research Council guidance. BMJ 2021;374:n2061.
[49] Pingree S, Hawkins R, Baker T, et al. The value of theory for enhancing and understanding e-health interventions. Am J Prev Med 2010;38:103–109.
[50] Stockwell S, Schofield P, Fisher A, et al. Digital behavior change interventions to promote physical activity and/or reduce sedentary behavior in older adults: a systematic review and meta-analysis. Exp Gerontol 2019;120:68–87.
[51] Van Rhoon L, Byrne M, Morrissey E, et al. A systematic review of the behaviour change techniques and digital features in technology-driven type 2 diabetes prevention interventions. Digit Health 2020;6.

Keywords: Steatotic liver disease; NAFLD; MASH; Diet; Physical activity.
Received 7 November 2024; received in revised form 23 June 2025; accepted 26 June 2025; Available online 2 July 2025

Journal of Hepatology, Volume *7*

Supplemental information

Effectiveness and active ingredients of digital behaviour change interventions for MASLD: A systematic review and meta-analysis

Hollie Smith, Rebecca Livingston, Kirsten Ashley, Matthew Cooper, Stuart McPherson, Alison Innerd, Kate Hallsworth, and Leah Avery

Effectiveness and active ingredients of digital behaviour change interventions for patients with MASLD: A systematic review and meta-analysis

Hollie Smith, Rebecca Livingston, Kirsten Ashley, Matthew Cooper, Stuart McPherson, Alison Innerd, Kate Hallsworth, Leah Avery

Table of contents

S1. PRISMA checklists……………………………………………………….…….....2

S2. Search strategies………………………………………………………………….5

S3. Study selection form……………………………………………………………7

S4. Data extraction form……………………………………………………………8

S5. SWIM checklist………………………………………………………………..22

S6. Outcome metrics……………………………………………………………...24

S7. Risk of bias assessments………………………………………………….....27

Supplementary file 1. PRISMA checklists.

Section and Topic	Item #	Checklist item	Location where item is reported
**TITLE**			
Title	1	Identify the report as a systematic review.	Title
**INTRODUCTION**			
Rationale	3	Describe the rationale for the review in the context of existing knowledge.	Intro (p6-8)
Objectives	4	Provide an explicit statement of the objective(s) or question(s) the review addresses.	Intro (p8)
**METHODS**			
Eligibility criteria	5	Specify the inclusion and exclusion criteria for the review and how studies were grouped for the syntheses.	Eligibility criteria (p9); data synthesis (p11)
Information sources	6	Specify all databases, registers, websites, organisations, reference lists and other sources searched or consulted to identify studies. Specify the date when each source was last searched or consulted.	Search strategy (p8-9)
Search strategy	7	Present the full search strategies for all databases, registers and websites, including any filters and limits used.	S2
Selection process	8	Specify the methods used to decide whether a study met the inclusion criteria of the review, including how many reviewers screened each record and each report retrieved, whether they worked independently, and if applicable, details of automation tools used in the process.	Study selection (p8-9)
Data collection process	9	Specify the methods used to collect data from reports, including how many reviewers collected data from each report, whether they worked independently, any processes for obtaining or confirming data from study investigators, and if applicable, details of automation tools used in the process.	Data extraction (p10)
Data items	10a	List and define all outcomes for which data were sought. Specify whether all results that were compatible with each outcome domain in each study were sought (e.g. for all measures, time points, analyses), and if not, the methods used to decide which results to collect.	Eligibility criteria (p9-10)
	10b	List and define all other variables for which data were sought (e.g. participant and intervention characteristics, funding sources). Describe any assumptions made about any missing or unclear information.	Data extraction (p10)
Study risk of bias assessment	11	Specify the methods used to assess risk of bias in the included studies, including details of the tool(s) used, how many reviewers assessed each study and whether they worked independently, and if applicable, details of automation tools used in the process.	Methodological quality assessment (p11)
Effect measures	12	Specify for each outcome the effect measure(s) (e.g. risk ratio, mean difference) used in the synthesis or presentation of results.	Data synthesis (p11)
Synthesis methods	13a	Describe the processes used to decide which studies were eligible for each synthesis (e.g. tabulating the study intervention characteristics and comparing against the planned groups for each synthesis (item #5)).	Data synthesis (p11)
	13b	Describe any methods required to prepare the data for presentation or synthesis, such as handling of missing summary statistics, or data conversions.	N/A

Section and Topic	Item #	Checklist item	Location where item is reported
	13c	Describe any methods used to tabulate or visually display results of individual studies and syntheses.	Table 1
	13d	Describe any methods used to synthesize results and provide a rationale for the choice(s). If meta-analysis was performed, describe the model(s), method(s) to identify the presence and extent of statistical heterogeneity, and software package(s) used.	Data synthesis (p11)
	13e	Describe any methods used to explore possible causes of heterogeneity among study results (e.g. subgroup analysis, meta-regression).	Data synthesis (p11)
	13f	Describe any sensitivity analyses conducted to assess robustness of the synthesized results.	N/A
Reporting bias assessment	14	Describe any methods used to assess risk of bias due to missing results in a synthesis (arising from reporting biases).	Methodological quality assessment (p11)
Certainty assessment	15	Describe any methods used to assess certainty (or confidence) in the body of evidence for an outcome.	Promise of intervention content and features (p11-12)
**RESULTS**			
Study selection	16a	Describe the results of the search and selection process, from the number of records identified in the search to the number of studies included in the review, ideally using a flow diagram.	Figure 1
	16b	Cite studies that might appear to meet the inclusion criteria, but which were excluded, and explain why they were excluded.	Figure 1
Study characteristics	17	Cite each included study and present its characteristics.	Table 1
Risk of bias in studies	18	Present assessments of risk of bias for each included study.	Methodological quality assessment (p21)
Results of individual studies	19	For all outcomes, present, for each study: (a) summary statistics for each group (where appropriate) and (b) an effect estimate and its precision (e.g. confidence/credible interval), ideally using structured tables or plots.	Figure 2 & Figure 3
Results of syntheses	20a	For each synthesis, briefly summarise the characteristics and risk of bias among contributing studies.	Figure 2 & Figure 3
	20b	Present results of all statistical syntheses conducted. If meta-analysis was done, present for each the summary estimate and its precision (e.g. confidence/credible interval) and measures of statistical heterogeneity. If comparing groups, describe the direction of the effect.	Weight (p13), ALT (p16), AST (p16)
	20c	Present results of all investigations of possible causes of heterogeneity among study results.	N/A
	20d	Present results of all sensitivity analyses conducted to assess the robustness of the synthesized results.	N/A
Reporting biases	21	Present assessments of risk of bias due to missing results (arising from reporting biases) for each synthesis assessed.	Methodological quality assessment

Section and Topic	Item #	Checklist item	Location where item is reported
			(p11)
Certainty of evidence	22	Present assessments of certainty (or confidence) in the body of evidence for each outcome assessed.	Active intervention ingredients (p18-21)
**DISCUSSION**			
Discussion	23a	Provide a general interpretation of the results in the context of other evidence.	Discussion (p21-25)
	23b	Discuss any limitations of the evidence included in the review.	Strengths and limitations (p25)
	23c	Discuss any limitations of the review processes used.	Strengths and limitations (p25)
	23d	Discuss implications of the results for practice, policy, and future research.	Conclusions (p25-26)
**OTHER INFORMATION**			
Registration and protocol	24a	Provide registration information for the review, including register name and registration number, or state that the review was not registered.	Search strategy (p8-9)
	24b	Indicate where the review protocol can be accessed, or state that a protocol was not prepared.	Search strategy (p8-9)
	24c	Describe and explain any amendments to information provided at registration or in the protocol.	Search strategy (p8-9)
Support	25	Describe sources of financial or non-financial support for the review, and the role of the funders or sponsors in the review.	Title page
Competing interests	26	Declare any competing interests of review authors.	Title page
Availability of data, code and other materials	27	Report which of the following are publicly available and where they can be found: template data collection forms; data extracted from included studies; data used for all analyses; analytic code; any other materials used in the review.	Supplementary files

From: Page MJ, McKenzie JE, Bossuyt PM, Boutron I, Hoffmann TC, Mulrow CD, et al. The PRISMA 2020 statement: an updated guideline for reporting systematic reviews. BMJ 2021;372:n71. doi: 10.1136/bmj.n71

For more information, visit: http://www.prisma-statement.org/

Supplementary file 2. Search strategies.

MEDLINE (EBSCO):

("non alcoholic fatty liver disease"[MeSH] OR “metabolic dysfunction-associated steatotic liver disease”[tiab] OR MASLD[tiab] OR NAFLD[tiab] OR MASH[tiab] OR NASH[tiab] OR “metabolic dysfunction-associated steatohepatitis”[tiab] OR "non alcoholic steatohepatitis"[tiab])

AND

("life style"[MeSH] OR "healthy eating"[tiab] OR diet[MeSH] OR "diet therapy"[MeSH] OR Exercise[MeSH] OR "Exercise Therapy"[MeSH] OR "physical activity"[tiab] OR "endurance training"[tiab] OR "strength training"[tiab] OR “resistance training”[tiab] OR "weight loss"[MeSH] OR "weight reduction"[tiab])

AND

(“telehealth” OR "tele health" OR “telemedicine” OR "tele medicine" OR "mobile health"[Title/Abstract:~3] OR “mhealth” OR "m health" OR “ehealth” OR "e health" OR "digital health"[Title/Abstract:~3] OR "digital therapeutics"[Title/Abstract:~3] OR "mobile phone" OR “smartphone” OR “wearable*”[tiab] OR "remote monitor"[Title/Abstract:~3] OR "remote monitoring"[Title/Abstract:~3] OR "mobile app"[Title/Abstract:~3] OR "mobile application"[Title/Abstract:~3] OR “web” OR “web-based” OR “text message” OR “self-management”)

CINAHL (EBSCO):

((MH "Nonalcoholic Fatty Liver Disease") OR (MH "Liver Diseases") OR (MH "Fatty Liver") OR "MASLD" OR (MH "Metabolic Diseases") OR "metabolic dysfunction-associated steatotic liver disease" OR "steatosis" OR "NASH") AND (“digital” OR “digital technology” OR “telehealth” OR “e−health” OR “ehealth” OR “tele−health” OR “mhealth” OR “mobile health units” OR “tele medicine” OR “telemedicine” OR “tele-medicine” OR “virtual medicine” OR “virtual” OR “app” OR “mobile” OR “mobile app” OR “mobile application” OR “mobile apps” OR “portable software app” OR “portable software application” OR “portable software applications” OR “portable software apps” OR “tablet application” OR “remote delivery” OR “text” OR “text message*” OR “SMS” OR “instant messag*” OR “phone” OR “cell” OR “cellphone” OR “cell-phone” OR “smartphone” OR “tablet” OR “tablet computer” OR “computer” OR “laptop” OR “PC” OR “email” OR “e-mail”) AND (“intervention” OR “digital N3 intervention*” OR “web-based intervention” OR “web intervention” OR “online intervention” OR “virtual intervention” OR “internet−based intervention” OR “internet−intervention” OR “online−based intervention” OR “online−intervention” OR “web intervention” OR “lifestyle intervention” OR “lifestyle modification” OR “lifestyle change”)

PsycINFO:

(DE “Liver Disorders” OR DE “Liver” OR “NAFLD” OR “NAFLD nonalcoholic fatty liver disease” OR “non alcoholic fatty liver disease” OR “non alcoholic hepato−steatosis” OR “non alcoholic hepatosteatosis” OR “non alcoholic liver steatosis” OR “non−alcoholic fatty liver” OR “nonalcoholic fatty liver” OR “nonalcoholic liver steatosis” OR “fatty liver disease” OR “hepatic steatosis” OR “hepatosteatosis” OR “liver steatosis”) AND (“digital” OR “digital technology” OR “telehealth” OR “e−health” OR “ehealth” OR “tele−health” OR “mhealth” OR “mobile health units” OR “tele medicine” OR “telemedicine” OR “tele-medicine” OR “virtual medicine” OR “virtual” OR “app” OR “mobile” OR “mobile app” OR “mobile application” OR “mobile apps” OR “portable software app” OR “portable software application” OR “portable software applications” OR “portable software

apps” OR “tablet application” OR “remote delivery” OR “text” OR “text message*” OR “SMS” OR “instant messag*” OR “phone” OR “cell” OR “cellphone” OR “cell-phone” OR “smartphone” OR “tablet” OR “tablet computer” OR “computer” OR “laptop” OR “PC” OR “email” OR “e-mail”) AND (“intervention” OR “digital N3 intervention*” OR “web-based intervention” OR “web intervention” OR “online intervention” OR “virtual intervention” OR “internet−based intervention” OR “internet−intervention” OR “online−based intervention” OR “online−intervention” OR “web intervention” OR “lifestyle intervention” OR “lifestyle modification” OR “lifestyle change”)

Web of Science:

((ALL=(NAFLD)) OR ALL=(fatty liver)) OR ALL=(steatosis)

AND

((((((((((((((((((ALL=(digital)) OR ALL=(app)) OR ALL=(web)) OR ALL=(ehealth)) OR ALL=(telehealth)) OR ALL=(virtual)) OR ALL=(phone)) OR ALL=(digital tech*)) OR ALL=(e-health)) OR ALL=(mhealth)) OR ALL=(telemedicine)) OR ALL=(tele medicine)) OR ALL=(tele-medicine)) OR ALL=(mobile)) OR ALL=(portable software)) OR ALL=(remote deliver*)) OR ALL=(text)) OR ALL=(internet)) OR ALL=(computer)) OR ALL=(email)

AND

(((ALL=(intervention)) OR ALL=(digital intervention)) OR ALL=(lifestyle intervention)) OR ALL=(lifestyle change)

Scopus:

(("fatty liver") OR ("steatosis")) AND (("digital") OR ("app") OR ("web") OR ("ehealth") OR ("telehealth") OR (“telemedicine”) OR (“tele-medicine”) OR ("virtual") OR ("phone") OR ("digital tech*") OR ("e-health") OR ("mhealth") OR ("telemedicine") OR ("mobile") OR ("portable software") OR ("remote deliver*") OR ("text") OR ("internet") OR ("computer") OR ("email")) AND (("intervention") OR ("digital intervention") OR ("lifestyle intervention") OR ("lifestyle change"))

Supplementary file 3. Study selection form.

Study selection form for assessing eligibility of full text papers.

**Study ID:**	
**Reviewer initials:**	
**Date:**	

**1. Participants**	**Yes/No/Unsure**
• Age 18+<br>• Diagnosis of MASLD/MASH with no other cause of liver disease (e.g. secondary causes of steatosis)	
**2. Intervention**	**Yes/No/U**
• Digital (e.g. web, app, SMS) lifestyle intervention program (e.g. diet, physical activity) for patients with MASLD<br>• NOT remote delivery of standard care i.e. a telephone consultation without a behavioural intervention component	
**3. Comparator/control**	**Yes/No/Unsure**
• No comparator/control<br>OR<br>• Usual/standard care in-person delivered behavioural interventions	
**4. Study design**	**Yes/No/Unsure**
• Any primary study design (e.g. RCT, pilot RCT, feasibility study) that includes pre- and post-intervention data OR intervention vs control data for the main outcomes	
**5. Main outcomes**	**Yes/No/Unsure**
Baseline and follow-up data concerning both:<br>• Metabolic outcomes (i.e. body weight, HbA1c)<br>• Liver outcomes (i.e. liver stiffness, histology)	

**Decision* (Include/Exclude/Discuss)**	
**1st reason**	

*To include: 1 and 2 and 3 and 4 and 5 must = YES

Supplementary file 4. Data extraction form.

Data Extraction Form

1. General Information

**Study ID**	
**Data extracted by:**	
**Date:**	
**Full bibliographic details:**	
**References of linked publications (e.g. protocol, intervention development, feasibility testing):**	
**Corresponding author contact details (where provided):**	

2. Study Characteristics

**Country of origin**	
**Aims and objectives**	
**Study design (Please circle)**	Definitive RCT<br>Pilot RCT<br>Qualitative study<br>Non-randomised controlled trial<br>Controlled before-and-after study

<table>
<tr><td></td><td colspan="3">Cohort Study
Case Control Study
Cross-sectional study
Mixed methods – please describe:
Other – please describe:</td></tr>
<tr><td>Number of study arms</td><td colspan="3"></td></tr>
<tr><td>Inclusion and exclusion criteria</td><td colspan="3">Inclusion:
Exclusion:</td></tr>
<tr><td>Sampling method (Please circle)</td><td colspan="3">Convenience/Opportunistic
Volunteer
Snowball
Random
Stratified
Other – please describe:</td></tr>
<tr><td>Sample size (Number of patients consented and randomised)</td><td>Total sample size (n=)</td><td>Intervention group (n=)</td><td>Control group (n=)</td></tr>
<tr><td>Sample size based on power analysis/sample size estimation?</td><td colspan="3">Yes/No</td></tr>
<tr><td>Sample size (as indicated by power analysis/sample size</td><td colspan="3">Yes/No</td></tr>
</table>

**estimation) achieved at final follow up?**	
**Intention to treat**	Yes/No
**Follow up time periods (e.g. baseline, 3 months etc.)**	
**Loss to follow up**<br><br>**(n, %) at each time period**	
**Conflicts of interest recorded**	

3. Participant Characteristics

	**Intervention group**	**Control group**
**Age**<br><br>(Mean/median/SD/IQR/range/not reported)		
**Gender**<br><br>(Frequency/percentage/not reported)		
**Ethnicity**<br><br>(Frequency/percentage/not reported)		

**BMI**<br>(Mean/median/SD/IQR/range/not reported)		
**Comorbidities**<br>(Mean/median/SD/IQR/range/not reported)		

4. Intervention Characteristics

**Name of intervention (e.g. VITALISE)**	
**Digital format of intervention (Please circle)**	Web-based<br>App-based<br>Text-messaging<br>Email<br>Video call<br>Other – Please describe:
**Location of delivery (Please circle)**	Primary care<br>Secondary care<br>Community/voluntary organisation<br>Other – Please describe:
**Who delivered the intervention?**	Primary care (GP)<br>Primary care (other HCP)<br>Secondary care (Consultant)

	Secondary care (other HCP)<br><br>Other – Please describe:
**Intervention frequency and duration (Duration of actual intervention delivery e.g. one 20 minute session; continuous access to intervention over 6 month period etc.)**	
**Intervention content (e.g. information with associated health coaching)**	
**Control content**	

5. Outcomes Assessed

	**Included?**<br><br>**(Yes/No)**	**Description**<br><br>**(Including details of data collection methods and/or scales used)**
Metabolic outcomes<br><br>(i.e. body weight, HbA1c, lipid profile measures)		
Liver outcomes (i.e. stiffness, histology)		
Lifestyle behaviour change (i.e. physical activity, dietary behaviours)		

6. Relevant Outcomes (Results/Findings)

Relevant outcome<br>(Please state)	Group	Baseline	Other follow up points	Final follow-up	Statistically significant difference between baseline and any follow up	Statistically significant difference between intervention and usual care groups at baseline and any follow-up
	Intervention					
	Control					
	Intervention					
	Control					
	Intervention					
	Control					
	Intervention					
	Control					

7. Development process

**Evidence of a systematic development process?**	**Was a framework used to inform intervention development?**	Yes/No/Not reported<br><br>Intervention Mapping<br>MRC Framework<br>Other – Please describe

	**Informed by evidence?**	Yes/No/Not reported
	**Informed by needs assessment with patients/relatives/stakeholders?**	Yes/No/Not reported
	**Evidence of 'testing' in patients/families/facilitators etc?**	Yes/No/Not reported
**Development underpinned by a theory/model of behaviour change, or by various constructs of a theory/model of behaviour change?**	Yes/No/Not reported<br><br>If yes, please describe:	
**Evidence of co-design/coproduction**	Yes/No/Not reported<br><br>If yes, please describe:	

8. Behaviour Change Techniques Used (BCTT v1) above and beyond control group

		Page(s) number where present	Comments
**1. Goals and planning**<br>1.1. Goal setting (behavior)<br>1.2. Problem solving<br>1.3. Goal setting (outcome)<br>1.4. Action planning<br>1.5. Review behavior goal(s)	Yes/No/Unsure		

1.6. Discrepancy between current behavior and goal<br>1.7. Review outcome goal(s)<br>1.8. Behavioral contract<br>1.9. Commitment			
**2. Feedback and monitoring**<br>2.1. Monitoring of behavior by others without feedback<br>2.2. Feedback on behaviour<br>2.3. Self-monitoring of behaviour<br>2.4. Self-monitoring of outcome(s) of behaviour<br>2.5. Monitoring of outcome(s) of behavior without feedback<br>2.6. Biofeedback<br>2.7. Feedback on outcome(s) of behavior	Yes/No/Unsure		
**3. Social support**<br>3.1. Social support (unspecified)<br>3.2. Social support (practical)<br>3.3. Social support (emotional)	Yes/No/Unsure		
**4. Shaping knowledge**	Yes/No/Unsure		

4.1. Instruction on how to perform the behavior<br>4.2. Information about antecedents<br>4.3. Re-attribution<br>4.4. Behavioral experiments			
**5. Natural consequences**<br>5.1. Information about health consequences<br>5.2. Salience of consequences<br>5.3. Information about social and environmental consequences<br>5.4. Monitoring of emotional consequences<br>5.5. Anticipated regret<br>5.6. Information about emotional consequences	Yes/No/Unsure		
**6. Comparison of behaviour**<br>6.1. Demonstration of the behavior<br>6.2. Social comparison<br>6.3. Information about others' approval	Yes/No/Unsure		
**7. Associations**	Yes/No/Unsure		

7.1. Prompts/cues<br>7.2. Cue signalling reward<br>7.3. Reduce prompts/cues<br>7.4. Remove access to the reward<br>7.5. Remove aversive stimulus<br>7.6. Satiation<br>7.7. Exposure<br>7.8. Associative learning			
**8. Repetition and substitution**<br>8.1. Behavioral practice/rehearsal<br>8.2. Behavior substitution<br>8.3. Habit formation<br>8.4. Habit reversal<br>8.5. Overcorrection<br>8.6. Generalisation of target behavior<br>8.7. Graded tasks	Yes/No/Unsure		
**9. Comparison of outcomes**<br>9.1. Credible source<br>9.2. Pros and cons<br>9.3. Comparative imagining of<br>future outcomes	Yes/No/Unsure		

**10. Reward and threat**<br><br>10.1. Material incentive (behavior)<br><br>10.2. Material reward (behavior)<br><br>10.3. Non-specific reward<br>10.4. Social reward<br><br>10.5. Social incentive<br>10.6. Non-specific incentive<br><br>10.7. Self-incentive<br>10.8. Incentive (outcome)<br><br>10.9. Self-reward<br>10.10. Reward (outcome)<br><br>10.11. Future punishment	Yes/No/Unsure		
**11. Regulation**<br><br>11.1. Pharmacological support<br><br>11.2. Reduce negative emotions<br><br>11.3. Conserving mental resources<br><br>11.4. Paradoxical instructions	Yes/No/Unsure		
**12. Antecedents**<br><br>12.1. Restructuring the physical environment<br><br>12.2. Restructuring the social environment	Yes/No/Unsure		

12.3. Avoidance/reducing exposure to cues for the behavior<br><br>12.4. Distraction<br><br>12.5. Adding objects to the environment<br><br>12.6. Body changes			
**13. Identity**<br><br>13.1. Identification of self as role model<br><br>13.2. Framing/reframing<br><br>13.3. Incompatible beliefs<br><br>13.4. Valued self-identify<br><br>13.5. Identity associated with changed behavior	Yes/No/Unsure		
**14. Scheduled consequences**<br><br>14.1. Behavior cost<br>14.2. Punishment<br>14.3. Remove reward<br>14.4. Reward approximation<br>14.5. Rewarding completion<br>14.6. Situation-specific reward<br>14.7. Reward incompatible behavior<br><br>14.8. Reward alternative behavior<br><br>14.9. Reduce reward frequency	Yes/No/Unsure		

14.10. Remove punishment			
**15. Self-belief**<br>15.1. Verbal persuasion about capability<br>15.2. Mental rehearsal of successful performance<br>15.3. Focus on past success<br>15.4. Self-talk	Yes/No/Unsure		
**16. Covert learning**<br>16.1. Imaginary punishment<br>16.2. Imaginary reward<br>16.3. Vicarious consequences	Yes/No/Unsure		

Supplementary file 5. SWIM checklist.

The citation for the Synthesis Without Meta-analysis explanation and elaboration article is: Campbell M, McKenzie JE, Sowden A, Katikireddi SV, Brennan SE, Ellis S, Hartmann-Boyce J, Ryan R, Shepperd S, Thomas J, Welch V, Thomson H. Synthesis without meta-analysis (SWiM) in systematic reviews: reporting guideline BMJ 2020;368:l6890 http://dx.doi.org/10.1136/bmj.l6890

**SWiM is intended to complement and be used as an extension to PRISMA**			
**SWiM reporting item**	**Item description**	**Page in manuscript where item is reported**	**Other***
Methods			
**1** Grouping studies for synthesis	1a) Provide a description of, and rationale for, the groups used in the synthesis (e.g., groupings of populations, interventions, outcomes, study design)	11	
	1b) Detail and provide rationale for any changes made subsequent to the protocol in the groups used in the synthesis	N/A	
**2** Describe the standardised metric and transformation methods used	Describe the standardised metric for each outcome. Explain why the metric(s) was chosen, and describe any methods used to transform the intervention effects, as reported in the study, to the standardised metric, citing any methodological guidance consulted	9-10	
**3** Describe the synthesis methods	Describe and justify the methods used to synthesise the effects for each outcome when it was not possible to undertake a meta-analysis of effect estimates	11	
**4** Criteria used to prioritise results for summary and synthesis	Where applicable, provide the criteria used, with supporting justification, to select the particular studies, or a particular study, for the main synthesis or to draw conclusions from the synthesis (e.g., based on study design, risk of bias assessments, directness in relation to the review question)	S3	

SWiM reporting item	Item description	Page in manuscript where item is reported	Other*
**5** Investigation of heterogeneity in reported effects	State the method(s) used to examine heterogeneity in reported effects when it was not possible to undertake a meta-analysis of effect estimates and its extensions to investigate heterogeneity	N/A	
**6** Certainty of evidence	Describe the methods used to assess certainty of the synthesis findings	P11	
**7** Data presentation methods	Describe the graphical and tabular methods used to present the effects (e.g., tables, forest plots, harvest plots).<br>Specify key study characteristics (e.g., study design, risk of bias) used to order the studies, in the text and any tables or graphs, clearly referencing the studies included	Table 1, Table 2 & Table 3	
Results			
**8** Reporting results	For each comparison and outcome, provide a description of the synthesised findings, and the certainty of the findings. Describe the result in language that is consistent with the question the synthesis addresses, and indicate which studies contribute to the synthesis	Table 1; p18-21	
Discussion			
**9** Limitations of the synthesis	Report the limitations of the synthesis methods used and/or the groupings used in the synthesis, and how these affect the conclusions that can be drawn in relation to the original review question	P25	

PRISMA=Preferred Reporting Items for Systematic Reviews and Meta-Analyses.
*If the information is not provided in the systematic review, give details of where this information is available (e.g., protocol, other published papers (provide citation details), or website (provide the URL)).

Supplementary file 6. Outcome metrics.

Study	Outcome measured	Metric of measurement
Axley et al., 2018	Weight	Lbs
	HDL	mg/dL
	TGs	g/dL
	ALT	IU/L
	AST	IU/L
Björnsdottir et al., 2024	Weight	Kgs
	HbA1c	mmol/L
	HDL	mmol/L
	LDL	mmol/L
	TGs	mmol/L
	Total cholesterol	mmol/L
	ALT	IU/L
	AST	IU/L
	Fat % (MRI-PDFF)	%
	Fibrosis (LSM)	kPa
	Steatosis (CAP)	dB/m
Cho et al., 2024	Weight	Kgs
	HDL	mmol/L
	LDL	mmol/L
	TGs	mmol/L
	Total cholesterol	mmol/L
	ALT	IU/L
	AST	IU/L
	ALP	IU/L
	GGT	IU/L
	Albumin	IU/L
Kaewdech et al., 2024	Weight	Kgs
	ALT	IU/L

	Fibrosis (LSM)<br>Steatosis (CAP)	kPa<br>dB/m
Kwon et al., 2024	Weight<br>ALT<br>AST<br>GGT	Kgs<br>IU/L<br>IU/L<br>IU/L
Lim et al., 2020	Weight<br>ALT<br>AST	Kgs<br>IU/L<br>IU/L
Mazzotti et al., 2018	Weight<br>TGs<br>ALT<br>GGT	%<br>mg/dl<br>IU/L<br>IU/L
Motz et al., 2021	Weight<br>HbA1c<br>ALT<br>AST<br>Fat % (MRI-PDFF)	%<br>%<br>IU/L<br>IU/L<br>%
Sato et al., 2023	Weight<br>HbA1c<br>LDL<br>TGs<br>ALT<br>AST<br>ALP<br>GGT	Kgs<br>%<br>mg/dl<br>mg/dl<br>IU/L<br>IU/L<br>IU/L<br>IU/L
Stine et al., 2023	Weight<br>ALT<br>AST<br>ALP	Kgs<br>IU/L<br>IU/L<br>IU/L
Tincopa et al., 2022	Weight<br>HbA1c	Lbs<br>mmol/L

	HDL	mg/dl
	LDL	mg/dl
	TGs	mg/dl
	ALT	IU/L
	Fibrosis (LSM)	kPa
	Steatosis (CAP)	dB/m

Supplementary file 7. Risk of bias assessments.

Summary of risk of bias (RoB 2 tool)

Summary of risk of bias (ROBINS-I tool)

Summary of risk of bias (NHLBI pre-post tool)

Study	Quality score	Quality rating
Björnsdottir et al., 2024	8/11 (72.7%)	Fair
Motz et al., 2021	5/11 (45.5%)	Poor
Sato et al., 2023	9/11 (81.8%)	Fair
Tincopa et al., 2022	7/11 (63.3%)	Fair
